# 3,5-Dialkoxypyridine analogues of bedaquiline are potent antituberculosis agents with minimal inhibition of the hERG channel

**DOI:** 10.1016/j.bmc.2019.02.026

**Published:** 2019-04-01

**Authors:** Hamish S. Sutherland, Amy S.T. Tong, Peter J. Choi, Adrian Blaser, Daniel Conole, Scott G. Franzblau, Manisha U. Lotlikar, Christopher B. Cooper, Anna M. Upton, William A. Denny, Brian D. Palmer

**Affiliations:** a Auckland Cancer Society Research Centre, School of Medical Sciences, New Zealand; b Maurice Wilkins Centre, University of Auckland, Private Bag 92019, Auckland 1142, New Zealand; c Institute for Tuberculosis Research, College of Pharmacy, University of Illinois at Chicago, 833 South Wood Street, Chicago, IL 60612, USA; d Global Alliance for TB Drug Development, 40 Wall St, NY 10005, USA

**Keywords:** CFU, colony-forming units, hERG (human Ether-a-go-go Related Gene), HPLC, high-performance liquid chromatography, LDA, lithium diisopropylamide, LiTMP, lithium tetramethylpiperidide, LORA, low oxygen recovery assay, MABA, microplate alamar blue assay, MDR, multidrug-resistant, MIC90, minimum concentration for 90% inhibition of growth, M. tb, mycobacterium tuberculosis, TB, tuberculosis

## Abstract

Bedaquiline is a new drug of the diarylquinoline class that has proven to be clinically effective against drug-resistant tuberculosis, but has a cardiac liability (prolongation of the QT interval) due to its potent inhibition of the cardiac potassium channel protein hERG. Bedaquiline is highly lipophilic and has an extremely long terminal half-life, so has the potential for more-than-desired accumulation in tissues during the relatively long treatment durations required to cure TB. The present work is part of a program that seeks to identify a diarylquinoline that is as potent as bedaquiline against *Mycobacterium tuberculosis*, with lower lipophilicity, higher clearance, and lower risk for QT prolongation. Previous work led to the identification of compounds with greatly-reduced lipophilicity compounds that retain good anti-tubercular activity *in vitro* and in mouse models of TB, but has not addressed the hERG blockade. We now present compounds where the C-unit naphthalene is replaced by a 3,5-dialkoxy-4-pyridyl, demonstrate more potent *in vitro* and *in vivo* anti-tubercular activity, with greatly attenuated hERG blockade. Two examples of this series are in preclinical development.

## Graphical abstract

**Figure:**
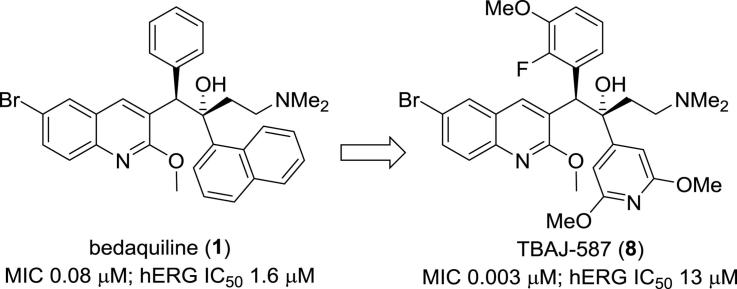

## 1 Introduction

Bedaquiline (TMC207, Sirturo, Janssen Pharmaceuticals; **1**; Table 2), a diarylquinoline, is the first example of a new class of drug that has proven clinically effective against drug-resistant tuberculosis (TB).^1^ It has a novel mechanism of action, selectively inhibiting the mycobacterial ATP synthase enzyme.^2^ In attempts to develop improved second-generation analogues of **1**, we have focused on two areas. One is to lower its very high lipophilicity (clogP 7.25), which may contribute to its long terminal elimination half-life and tissue accumulation at high doses.^3^ The other aim is to attenuate its inhibition (IC_50_ 1.6 µM) of the cardiac potassium channel protein coded by the human ether-a-go-go-related gene (hERG).^4^ This forms the pore-forming subunit of the rapidly activating delayed rectifier potassium channel (IKr), which is important for cardiac repolarization. Dysfunction of hERG causes long QT syndrome and can increase the risk of sudden death in patients with cardiac ischemia - a potential safety issue that all drug candidates seeking regulatory approval must currently address. In our attempts to develop analogues of lower lipophilicity, we have reported on the utility of replacing the 6-Br substituent on the A-unit quinoline with a more polar cyano group,^5^ replacing the B-unit phenyl with heterocycles,^6^ and replacing the C-unit naphthalene with either bicyclic heterocycles^7^ or substituted pyridines.^8^ These studies provided a diverse range of potent diarylquinolines with much lower lipophilicity, and defined structure-activity relationships between antitubercular activity and lipophilicity. While they were, in the main, less successful in identifying active compounds with significantly attenuated potency against the hERG channel, we recently^8^ reported encouraging results (hERG IC_50_ values around or >10 µM) in a small number of compounds containing a 4-pyridyl-3,5-dialkoxy C-unit, suggesting this substitution is a promising one for mitigation of hERG potency. In the present paper, we follow up on that observation with a more extensive structure-activity study of this motif, which has allowed selection of two candidate compounds for preclinical development.

## 2 Results and discussion

### 2.1 Chemistry

**Scheme 1:**
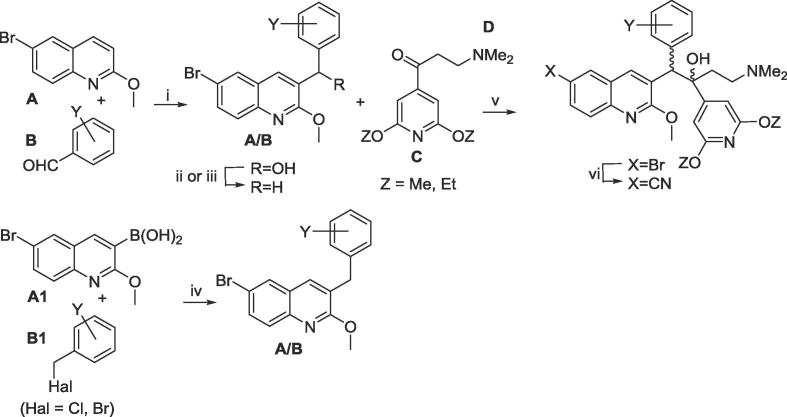
Synthesis of substituted analogues of 1. *Reagents and conditions*: (i) LiTMP, THF, −75 °C, 1.5 h then the appropriate aldehyde **B**, −75 °C, 4 h; (ii) Et_3_SiH, TFA, DCM; (iii) MsCl, Et_3_N, DMF, then NaBH_4_; (iv) Cs_2_CO_3_, Pd(PPh_3_)_4_, PhMe/DMF, 110 °C (sealed tube), 5 h; (v) LDA, THF, −75 °C, 1.5 h then the appropriate ketone **C/D**, then HOAc; (vi) Zn/Zn(CN)_2_, Pd_2_(dba)_3_/P(o-tol)_3_, DMF, 50 °C, then separation of the diastereomers by SFC HPLC.

The bedaquiline analogues of Table 2 were prepared as previously described,^5^, ^6^, ^7^, ^8^, ^9^ by LDA-mediated coupling of the appropriate benzylquinoline A/B-units and 1-(2,6-dialkoxypyridin-4-yl)-3-(dimethylamino)propan-1-one (Mannich base) C/d-units (Scheme 1). The 6-cyano derivatives were prepared by palladium-catalyzed cyanation of the corresponding bromo analogues. The resulting diarylquinolines were formed as a racemic mixture of four diastereomers, and the desired 1*R,*2*S* diastereomer (depicted) was isolated by super-critical fluid HPLC at BioDuro LLC (Beijing).

**Scheme 2:**
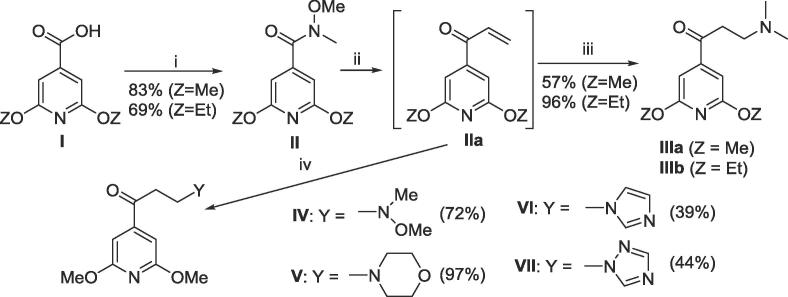
Synthesis of dialkoxypyridyl Mannich bases (III-VII) *Reagents and conditions*: (i) COCl_2_, DMF, DCM, then MeNH(OMe).HCl, pyridine; (ii) vinylmagnesium bromide, THF; (iii) dimethylamine, water; (iv) for **IV**, water; **V**, morpholine, water; **VI** and **VII**, isolation of crude **IIa** then imidazole or triazole.

Having previously established the utility of a range of substituted pyridyl B- and/or C-units^6,8^ in lowering overall clogP values, in the present SAR study we report 3,5-dialkoxy-4-pyridyl C-unit analogues bearing a wide range of different B-unit substitutions, with the focus on mitigation of hERG potency. Thirty-four different A/B-units were used, apart from the unsubstituted parent; syntheses of many of these (for compounds **7**, **9**, **10**, **13**, **14**, **16, 17**, **23**–**26**, **34**, **40**–**42**, **48**, **50** of Table 2) have already been reported.^5^, ^6^, ^7^, ^8^ The remainder were prepared as outlined in Scheme 1 and Table 1.

**Table 1:** Synthesis of new A/B units.

Y	Name	Steps	Overall yield (%)^a^	For Table 3 compounds
2,3-(CH_2_)_3_	AB-1	3	84	**2,3**
2,3-(CH_2_)_4_-	AB-2	3	41	**4**
2-F, 3-Me	AB-3	1	76	**5**–**7**
3-F, 4-OMe	AB-4	3	37	**11**
2,3-OCH_2_O-	AB-5	1	76	**15, 16**
2,3-OCH=CH–	AB-6	3	52	**21**
3-aza, 4-NEt_2_	AB-7	2	43	**24**–**26**
3-aza, 2-OMe, 5-O^i^Pr	AB-8	4	20	**29, 30**
3-aza, 2,4,5-triOMe	AB-9	5	40	**31, 32**
4-aza, 2,5-diOMe	AB-10	5	16	**34**
4-aza, 3,5-diSMe	AB-11	3	21	**36**
4-aza, 3,5-diSEt	AB-12	4	37	**37**
4-aza, 2-OMe, 5-O^i^Pr	AB-13	9	11	**38, 39**
4-aza, 3-OMe, 5-O^cy^Bu	AB-14	5	55	**43**
4-aza, 3-OEt, 5-O^i^Pr	AB-15	5	58	**44, 45**
4-aza, 2,3,5-triOMe	AB-16	8	13	**46**
4-aza, 3-OMe, 5-NMe_2_	AB-17	2	51	**47, 48**
4-aza, 3-OEt, 5-NMe_2_	AB-18	3	42	**49, 50**
4-aza, 3-SEt, 5-NMe_2_	AB-19	4	37	**51**
4-aza, 2-F, 3-OMe	AB-20	5	12	**52**

a The yield quoted is for the mixture of four stereoisomers formed in the condensation reaction.

**Table 2:** Inhibitory properties of 3,5-dialkoxy-4-pyridyl analogues of bedaquiline.

**Figure:**
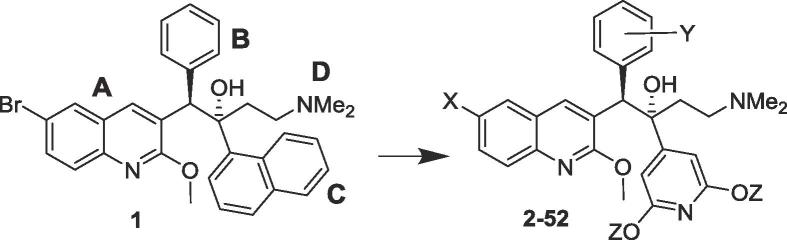

No	X	Y	Z	Yld	MIC_90_ (µg/mL)^b^		clogP^c^
				(%)^a^	MABA	LORA	
**1**					0.04	0.08	7.25
**2**	Br	2,3-(CH_2_)_3_	Me	24	0.01	0.02	6.81
**3**	CN	2,3-(CH_2_)_3_-	Me	71^d^	0.02	0.02	5.45
**4**	Br	2,3-(CH_2_)_4_-	Me	13	<0.004	0.01	7.38
**5**	Br	2-F, 3-Me	Me	61	<0.01	<0.01	6.44
**6**	Br	2-F, 3-Me	Et	67	<0.02	<0.02	7.50
**7**	CN	2-F, 3-Me	Et	74^d^	<0.02	<0.02	6.14
**8**	Br	2-F, 3-OMe	Me	52	0.006	<0.02	5.80
**9**	CN	2-F, 3-OMe	Me	80^d^	0.09	0.09	4.44
**10**	Br	2-F, 3-OMe	Et	70	0.02	0.08	6.86
**11**	Br	3-F, 4-OMe	Me	38	0.01	0.01	5.80
**12**	Br	2, 3-diOMe	Me	63	<0.01	<0.01	5.06
**13**	CN	2, 3-diOMe	Me	80^d^	0.12	0.14	3.70
**14**	Br	2,3-diOMe	Et	64	<0.02	<0.02	6.11
**15**	Br	2,3-OCH_2_O-	Me	66	<0.02	0.06	5.76
**16**	CN	2,3-OCH_2_O-	Me	85^d^	0.06	0.07	4.41
**17**	Br	2,3-O(CH_2_)_2_O–	Me	72	0.02	0.02	5.72
**18**	CN	2,3-O(CH_2_)_2_O–	Me	76^d^	0.02	0.02	4.90
**19**	Br	2,3-O(CH_2_)_2_O–	Et	52	0.07	0.13	6.78
**20**	CN	2,3-O(CH_2_)_2_O–	Et	62^d^	0.03	0.03	5.42
**21**	Br	2,3-OCH = CH–	Me	16	<0.004	0.01	6.36
**22**	Br	3-aza, 2-OMe	Me	65	<0.02	<0.02	4.72
**23**	CN	3-aza, 2-OMe	Me	68^d^	0.04	0.25	3.36
**24**	Br	3-aza, 4-NEt_2_	Et	26	<0.02	<0.02	7.13
**25**	Br	3-aza, 4-NEt_2_	Me	46	0.003	0.008	6.07
**26**	CN	3-aza, 4-NEt_2_	Me	68^d^	<0.01	<0.02	4.71
**27**	Br	3-aza, 2,5-diOMe	Me	38	<0.02	<0.02	5.12
**28**	CN	3-aza, 2,5-diOMe	Me	44^d^	0.02	0.04	3.76
**29**	Br	3-aza, 2-OMe, 5-O^i^Pr	Me	38	<0.004	0.016	5.96
**30**	CN	3-aza, 2-OMe, 5-O^i^Pr	Me	56	<0.004	<0.004	4.60
**31**	Br	3-aza, 2,4,5-triOMe	Me	38	<0.01	<0.01	5.15
**32**	CN	3-aza, 2,4,5-triOMe	Me	39^d^	<0.01	<0.01	3.79
**33**	Br	4-aza, 2,3-diOMe	Me	62	<0.02	0.07	4.77
**34**	Br	4-aza, 2,5-diOMe	Me	77	<0.01	0.04	5.12
**35**	Br	4-aza, 2,3-diOMe	Et	32	<0.02	<0.02	5.83
**36**	Br	4-aza, 3,5-diSMe	Me	26	<0.01	<0.01	6.18
**37**	Br	4-aza, 3,5-diSEt	Me	28	<0.02	<0.02	7.24
**38**	Br	4-aza, 2-OMe, 5-O^i^Pr	Me	54	<0.004	0.008	5.96
**39**	CN	4-aza, 2-OMe, 5-O^i^Pr	Me	39^d^	0.05	0.09	4.60
**40**	Br	4-aza, 3-OMe, 5-O^i^Pr	Me	57	<0.01	<0.01	6.36
**41**	CN	4-aza, 3-OMe, 5-O^i^Pr	Me	57 ^d^	0.01	0.01	5.00
**42**	Br	4-aza, 3-OMe, 5-O*^n^*Pr	Me	52	0.01	0.01	6.58
**43**	Br	4-aza, 3-OMe, 5-O^cy^Bu	Me	16	0.01	0.12	6.43
**44**	Br	4-aza, 3-OEt, 5-O^i^Pr	Me	61	<0.01	<0.01	6.88
**45**	CN	4-aza, 3-OEt, 5-O^i^Pr	Me	80^d^	0.01	0.01	5.53
**46**	Br	4-aza, 2,3,5-triOMe	Me	47	0.004	0.006	5.15
**47**	Br	4-aza, 3-OMe, 5-NMe_2_	Me	21	0.01	0.03	5.83
**48**	CN	4-aza, 3-OMe, 5-NMe_2_	Me	71 ^d^	0.03	0.09	4.48
**49**	Br	4-aza, 3-OEt, 5-NMe_2_	Me	58	>0.004	0.03	6.36
**50**	CN	4-aza, 3-OEt, 5-NMe_2_	Me	90^d^	0.03	0.05	5.01
**51**	Br	4-aza, 3-SEt, 5-NMe_2_	Me	52	0.01	0.01	6.68
**52**	Br	4-aza, 2-F, 3-OMe	Me	54	<0.01	<0.05	5.10

a Yields (Yld) in the AB/CD coupling step to give bedaquiline analogues (as racemic mixtures). The desired 1*R*, 2*S* diastereomer was then isolated by SFC HPLC at BioDuro LLC, Beijing. ^b^MIC_90_ (µg/mL); minimum inhibitory concentration for 90% inhibition of growth of *M.tb* strain H37Rv, determined under aerobic (replicating; MABA) (ref. 10) or non-replicating (LORA) (ref. 11) conditions, determined at the Institute for Tuberculosis Research, University of Illinois at Chicago. ^c^clogP calculated by ChemDraw Ultra v12.0.2. (CambridgeSoft). ^d^Yields for the Br/CN conversion.

The acid chlorides derived from **I** were reacted with *N*,*O*-dimethylhydroxylamine to give the *N*-methoxyacetamides (Weinreb amides) (**II**), which were converted to the required Mannich bases (**III**) in high yields by reaction with vinyl magnesium bromide to generate an intermediate (**IIa**), followed by reaction with dimethylamine. Mannich bases **IV** to **VII** were prepared to evaluate the role of varying D unit pKa on hERG inhibition (see Table 4). In the absence of an additional dialkylamine source, the *N*,*O*-dimethylhydroxylamide liberated in the formation of **IIa** itself reacts with **IIa** to give **IV**. The imidazole (**VI**) and triazole (**VII**) derivatives required the isolation of crude **IIa** before subsequent reaction with imidazole or triazole, this avoided the competitive reaction between **IIa** and the more nucleophilic *N*,*O*-dimethylhydroxylamine.

### 2.2 Structure-activity relationships

In previous work,^8^ we demonstrated the ability of a range of analogues with substituted pyridyl C-units to significantly lower lipophilicity (clogP values between about 5.5–4.0) while producing compounds only slightly less effective than **1** against both replicating and non-replicating cultures of *M.tb in vitro*, and in a mouse TB infection model. While these compounds, as a class, did not show useful reductions in potency in hERG channel blockade, the 3,5-dimethoxy-4-pyridyl unit did appear to have some utility, prompting the present study of 3,5-dimethoxy-4-pyridyl and 3,5-diethoxy-4-pyridyl analogues with a wide range of B-units.

Table 2 provides data on analogues of **1** (**2**–**52**), where the naphthalene C-unit has been replaced by either a 3,5-dimethoxy-4-pyridyl or a 3,5-diethoxy-4-pyridyl unit. The compounds were evaluated for bacterial growth inhibition (MIC_90_ values) in cultures of *M.tb* (strain H37Rv) under both replicating (MABA assay^10^) and non-replicating (LORA assay^11^) conditions.

The new bedaquiline analogues characterized in the present work have calculated clogP values between 7.50 and 3.36, with the majority between 6.25 and 4.25 (Table 2). Nearly all have MIC_90_ values significantly superior (many by>10-fold) to **1** against both replicating and non-replicating cultures of *M.tb*. As shown previously,^7^, ^8^ the more lipophilic compounds appear more potent, but as so many had indeterminate endpoint values (below the testing range of the assay), this could not be quantified. We have previously shown^8^ that different B-units have little specific effect on inhibitory activity (MIC_90_s) apart from their contribution to overall lipophilicity. This appears to also be the case for the analogues tested here.

Representative compounds in the series were evaluated *in vitro* for a range of ADME and toxicological properties (Table 3). Compounds were tested for cytotoxicity in Vero green monkey-derived epithelial kidney cells,^12^ and for inhibition of CYP 3A4, the major metabolising enzyme for **1**.^12^ All tested compounds had IC_50_s > 10 µg/mL in the Vero assay, except for compound **9**, which had a value of 7.3, and compounds **28** and **34**, where this was not measured. In comparison, the value for **1** in repeat assays was between 4 and 16 µg/mL. Where tested, compounds had IC_50_s > 10 µM for CYP3A4 inhibition (bedaquiline IC_50_ > 40 µM), however, compounds **3**–**7**, **11**, **19**–**21**, **24**, **27**–**28**, **30**–**31**, **33**, **36**, **41**–**42**, **45**–**49**, **51**–**52** were not tested in this assay.

**Table 3:** Additional biological data on selected compounds of Table 2.

No	hERG^a^	Aq. Sol	Microsomal stability		IV Cl	Vz	AUC_inf_	F	Log redn. in mouse lung CFU		clogP^i^
	IC_50_ (µM)	pH 7.4 µM	HCl_int_^b^	MCl_int_^b^	mL/min/kg	L/kg	µg*hr/mL^d^	%^e^	Test compd^g^	Beda-quiline^h^	
**No**	hERG^a^	Aq. Sol.	HCl_int_^b^	MCl_int_^b^	IV Cl^c^	Vz^d^	AUC_inf_^e^	F^f^			
**1**	1.6	<0.06	3	7	7	22	20.9	56		4.5–6.2	7.25
**3**	4.5	0.05	4	8	23	51	5.54	75	>5.0	5.0	5.45
**4**	>10	<0.06	6	8	9.3	29	7.06	39	>5.0	5.0	7.38
**5**	>10	<0.04	4	6	10	34	12.8	69	>5.0	4.6	6.44
**6**	>10	<0.02	3	6	8.2	22	9.11	39	4.0	4.6	7.50
**7**	>10	<0.06	0.7	1	3.2	15	28.8	54	>5.0	4.5	6.14
**8**	13	<0.06	2	18	48	95	1.72	48	5.2	6.1	5.80
**10**	10	<0.06	3	10	19	53	4.95	53	4.4	4.9	6.86
**11**	>10	<0.02	6	7	7.0	32	21.7	94	5.5	>5	5.80
**12**	2.2	0.42	14	15	23	30	2.39	26	1.2	5.7	5.06
**14**	5.5	<0.02	1	7	10	29	7.74	45	4.0	5.6	6.11
**16**	1.6	0.70	6	36	41	27	1.18	29	2.6	5.3	4.41
**18**	4.4	2.2	7	27	44	22	1.08	28	4.8	5.6	4.90
**19**	13.3	<0.06	4	2	5.2	16	20.0	56	4.4	4.6	6.78
**20**	4.5	0.96	1	2	13	18	6.11	47	>5.0	5.0	5.42
**21**	>10	<0.06	3	10	8.8	24	14.2	66	>5.0	5.0	6.36
**22**	1.7	0.38	6	33	28	37	1.58	26	4.0	5.3	4.72
**24**	9.9	<0.02	65	15	14	67	4.74	39	4.6	5.6	7.13
**27**	2.2	0.38	6	20	25	26	3.12	46	>5.0	5.0	5.12
**28**	3.1	12.5	6	51	41	94	0.96	23	4.2	6.1	3.76
**30**	2.4	0.28	2	16	52	47	1.39	43	4.8	4.9	4.60
**31**	5.9	0.03	9	57	25	69	2.69	53	>5.0	5.0	5.15
**33**	2.6	0.40	8	24	25	42	5.33	79	4.8	5.3	4.77
**36**	>10	<0.04	stable	6	6.2	27	10.9	57	5.0	5.5	6.18
**41**	10.6	<0.02	2	29	21	73	2.44	22	4.9	5.6	5.00
**42**	>30	<0.02	stable	4	4.1	12	20.6	46	5.5	5.5	6.58
**45**	>30	<0.02	2	21	13	38	4.21	32	>5.0	4.6	5.53
**46**	>30	<0.02	2	22	13	31	5.61	44	>5.5	>5.5	5.15
**47**	>10	<0.02	9	9	3.6	49	12.3	41	4.8	5.0	5.83
**48**	9.4	0.59	13	25	24	38	3.31	45	2.5	5.0	4.48
**50**	>10	0.57	4	58	20	54	1.70	20	3.8	4.9	5.01
**51**	>10	<0.02	4	11	7.0	30	5.33	28	4.7	5.5	6.68
**52**	>10	<0.2	4	6	6.8	20	22.7	84	>5.5	>5	5.10

a Inhibition of hERG (IC_50_ in µM); ^b^Clearance of compound by human or mouse liver microsomes (μL/min/mg protein); ^c^ IV clearance, mouse (mL/min/kg); ^d^IV apparent volume of distribution during terminal phase, mouse (L/kg); ^e^Oral exposure, area under the curve 0 to infinity, in mice (µg*h/mL); ^f^Oral bioavailability in mice; ^g^Log reduction in lung colony-forming units (CFU) compared to vehicle of analogues or ^h^**1** when dosed in the same assay at 20 mg/kg daily in mice for 12 days, beginning 10 days after *M.tb* inoculation via the aerosol route; ^i^clogP calculated by ChemDraw Ultra v12.0.2. (CambridgeSoft).

**Table 4:** Exploration of alternative C/d-units.

**Figure:**
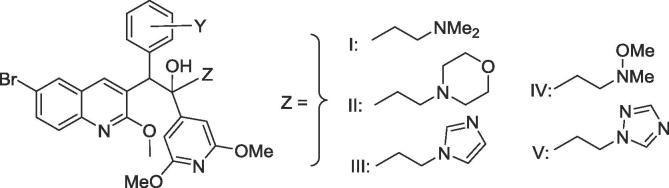

No	Y	Z	Yld	MIC_90_ (µg/mL)^a^		hERG^b^	clogP^c^	pKa^d^
				MABA	LORA			
**12**	2,3-diOMe	I	13	<0.01	<0.01	2.2	5.06	9.05
**53**	2,3-diOMe	II	53	>5	>5		4.99	6.84
**54**	2,3-diOMe	III	20	0.39	2.37	11	4.65	6.85
**55**	2,3-diOMe	IV	71	>5	2.47		5.04	2.85
**8**	2-F, 3-OMe	I	80	0.003	<0.02	13	5.80	9.04
**56**	2-F, 3-OMe	II	92	>5	>5		5.74	6.83
**57**	2-F, 3-OMe	III	51	2.42	2.2		5.39	6.85
**58**	2-F, 3-OMe	IV	51	2.54	>5		5.78	2.84
**59**	2-F, 3-OMe	V	65	2.3	>5		4.32	2.80
**33**	4-aza, 2,3-diOMe	I	47	<0.02	0.07	2.6	4.77	8.98
**60**	4-aza, 2,3-diOMe	II	70	4.2	5.0		4.71	6.76
**61**	4-aza, 2,3-diOMe	III	32	0.07	0.46	5.8	4.36	6.83
**62**	4-aza, 2,3-diOMe	V	45	2.02	2.37		3.29	2.77

^a-c^As for Table 1. ^d^pKa values for the d-unit side chain calculated using ACD/PhysChem Suite v12: ACD Labs, Toronto, Canada.

The most interesting result of this study was the very positive effect of the 3,5-dialkoxy-4-pyridyl C-unit substitution on hERG potassium channel inhibition. Of the 33 compounds evaluated, 19 had IC_50_ values of **≥** 10 µM, in strong contrast with previous studies of different sets of analogues,^6^, ^7^, ^8^ where no compound had IC_50_ values even approaching 10 µM. The data suggest that, for this series, potent hERG inhibition is broadly related to higher compound lipophilicity; the 14 analogues with hERG values<10 µM had an average clogP of 4.57, whereas the 19 analogues with hERG values > 10 µM had an average clogP of 6.17 (Table 2). Put another way, 15/17 compounds with clogP > 5.5 had hERG IC_50_s > 10 µM, compared to only 4/16 with clogP < 5.5.

Table 3 also provides data on the *in vitro* clearance of the analogues from human and mouse liver microsomes. The very slow clearance (CL_int_ 3 μL/min/mg) and concomitantly long half-life (231 min) of **1** in human liver microsomes is improved upon 2-fold by many of the analogues but a 3-fold or more increase in human microsomal clearance was seen for a handful of analogues only. The mouse liver microsome data did not always reflect the corresponding human data. For example, for several compounds (**16, 18, 22, 28, 41, 45, 46** and **50**), the Cl_int_ in human microsomes is similar to, or 2-fold higher than, that of **1**, but in mouse microsomes, they show at least 3-fold higher clearance than **1**. This may be due to differing relative contributions to metabolism of these compounds versus **1** for CYP isoforms in human versus mouse microsomes. The human microsome data may better predict the human clearance for these compounds, if microsomal metabolism is representative of metabolism overall, for these compounds. Studies of pharmacokinetics in several species, followed by modelling work, will be needed to provide a better prediction of human PK.

The pharmacokinetics in mice were evaluated after administration of a single oral dose and a single intravenous injection. Clearance *in vivo* ranged from low (similar to that of **1**) to moderate, around 50 mL/min/kg, and did not always reflect the *in vitro* Cl_int_ values. In particular, a significantly higher *in vitro* Cl_int_ than **1** did not always lead to much higher *in vivo* clearance (eg compounds **31** and **41**). Although all compounds shown demonstrated high volumes of distribution (12 L/kg and higher), compounds such as **31** and **41** showed particularly high volume of distribution values, and higher tissue levels in these cases may have contributed to lower *in vivo* clearance than predicted by *in vitro* Cl_int_.

Bioavailability (F) ranged from moderate (20% for compound **50**) to very high (94% for compound **11**) and, after oral administration, a range of AUC values was seen. The AUC values correlated broadly with clearance values, suggesting that for the large part, plasma exposures varied depending on relative clearance. Regarding bioavailability, for the compounds with F of 30% or lower, while they showed clearance in mice of 20 mL/min/kg and higher (except compound **51**, which showed lower clearance), they did not necessarily show the highest *in vivo* clearance values overall. Of note, compounds with F of 30% and below had clogP values of 5.53 and below, except for compound **51**, suggesting perhaps lower clogP was one factor associated with lower %F for this series. All compounds tested demonstrated human plasma protein binding of >99.9%, with the exceptions of compounds **18**, **28**, and **30**, which were 99.85%, 99.78%, and 99.86% bound, respectively (data not shown). Assuming mouse plasma protein binding is similar, relative PPB presumably does not play a role in relative PK for these compounds.

Evaluation of the compounds for *in vivo* efficacy used a mouse model of TB. Female BALB/c mice (n = 7 per group) were dosed at 20 mg/kg daily, by oral gavage, for 12 days, beginning 10 days after *M.tb* H37Rv inoculation by the aerosol route. Compounds were formulated in hydroxy-beta-cyclodextrin 20%. The relative *in vivo* activity was assessed by the log reduction in colony-forming units (CFU) from lung homogenates, an indicator of bacterial burden in the lungs, following treatment with a test compound compared to that effected by vehicle administration only, and to that demonstrated by bedaquiline, dosed at 20 mg/kg in the same assay run. In this mouse model of TB, the CFU reach approximately 5–6 log in the lungs in the vehicle-treated control group. Hence a reduction in lung CFU of 5 of more log units implies complete clearance of the recoverable lung CFU following 12 days of treatment. Therefore, for compounds in Table 3 where a reduction of approximately 5 log CFU is shown, complete clearance was achieved, and it is not possible to further distinguish the relative efficacy between these compounds. Reductions of 4 log CFU and higher mean only very few bacteria were remaining in the lungs of mice and these compounds can therefore be considered to show similar efficacy to bedaquiline within the range of these experiments.

Gratifyingly, and in contrast to a previous study of bedaquiline analogues with differently-configured substituted C-unit pyridyl substituents,^8^ most of the compounds showed *in vivo* activities similar to that of **1**, at the same dose, with only compounds **12**, **14** and **48** being less active, showing CFU reductions of <4 log units (Table 3). It was interesting to note that the compounds demonstrating smaller CFU reductions *in vivo* were not the compounds with the lowest AUC values and, indeed, several compounds demonstrated CFU reductions that were at least similar to that effected by bedaquiline (within the limits of the assay), with substantially lower AUC values. For example, compounds **8, 30, 31, and 41** demonstrated CFU reductions within one log unit of the CFU reduction effected by bedaquiline, with AUC values of 2.69 µg*hr/mL or below, compared to 20.9 µg*hr/ml for **1**. In general, for the tested compounds, where similar efficacy was achieved with lower AUCs, this is likely due to the lower MICs for these compounds than for bedaquiline, and consistent with previous work that has demonstrated AUC/MIC as the driver of efficacy against murine TB for bedaquiline. It may also be due to formation of metabolites that are active against *M.tb*, as is seen for bedaquiline.^13^ One exception was compound **10**, where efficacy in the range of that of bedaquiline was seen, with a 4-fold lower AUC, and a similar MIC. In this case, it is possible that some other factor such as tissue distribution, contributed to the CFU reduction seen at the lower AUC.

Several compounds presented here, namely **3**, **8, 10, 18, 22, 24, 27, 28, 30, 31, 33, 41 45, 46, 50** and **51** demonstrated 4 log unit CFU reductions in mice, at an AUC at least approximately 4-fold lower than that of bedaquiline. This suggests these analogues may have potential to demonstrate efficacy against TB in patients at lower plasma levels than bedaquiline. Of these, compounds **8**, **10**, **24**, **41**, **45**, **46**, **50** and **51** exhibited hERG IC_50_s of 9.9 µM or higher, compared to 1.6 µM for bedaquiline. Taken together, a higher IC_50_ against hERG along with a lower efficacious exposure may predict a lower risk of QTc prolongation in patients for these analogues compared to bedaquiline. Although not all of these compounds showed higher human microsome Cl_int_ than bedaquiline, the possible lower risk of QTc prolongation along with potential for lower overall adverse effects based on a lower efficacious exposure, resulted in selection of these compounds, as well as others in Table 3 that demonstrated similar efficacy to bedaquiline with lower potency against hERG, for further evaluation. Electrocardiography studies in animals and ultimately in human subjects will be needed to determine whether the decreases in potency against hERG seen here, along with the decreased plasma levels needed for efficacy for some of these compounds, translates into an absence of QTc prolongation at a therapeutic dose and exposure.

Finally, the overall good activity profile of this series of 3,5-dialkoxy-4-pyridyl analogues was utilized to explore the results of changes in the pKa values of the d-unit side chain. This was explored previously by Guillemont et al.^9^ in their original paper, where they concluded that a sidechain with a pKa of >8 was needed to retain activity against the related mycobacterium, *M. smegmatis*. We were thus motivated by the idea that utilising a much weaker base in the side chain in analogue series with intrinsically higher potency might further attenuate hERG blockade while retaining good *M.tb* potency. The results in Table 4 suggest that a weaker base did lead to reduced hERG inhibition (compare hERG data for **12** and **54**, **33** and **61**), but there was also a large increase in the MICs for inhibition of *M.tb*.

## 3 Conclusions

In previous publications in this series, we have shown that less lipophilic analogues of **1** (clogP 7.25) retain substantial *in vitro* and *in vivo* activity against *M.tb*, down to compounds with a clogP of about 4; this shows that one of the potential drawbacks of **1** can be ameliorated. In the present paper, we show that substitution of the C-unit naphthalene of **1** with a 3,5-dialkoxy-4-pyridyl group provide analogues with not only higher potency and substantially lower clogP than **1**, but also with a greatly reduced potential cardiotoxicity risk profile, due to significantly reduced blockade of the hERG potassium channel. This work has recently allowed the selection of analogues **8** (TBAJ-587)^14^, ^15^ and **46** (TBAJ-876)^16^ as compounds for pre-clinical development. Based on the preliminary data presented in this paper, several compounds (including **8** and **46**), with activity against murine TB similar to bedaquiline at the same dose, with lower clogP, higher IC_50_ against hERG, and, in most cases, higher Cl values in human microsomes, were selected to progress to further studies. Those advanced studies evaluated the PK in rodents and non-rodents, MICs against clinical isolates of *M. tb*, efficacy against a model of chronic murine TB, and toxicity in a preliminary rat toxicity and toxicokinetics study. Based on that data, compounds **8** and **46** were selected due to their lower MICs than bedaquiline against clinical isolates of *M.tb*, efficacy demonstrated against murine TB at lower exposures than bedaquiline, lower potency against hERG, predicted higher human clearance, and an acceptable safety margin, based on the safe exposure in rats compared to the efficacious exposure in mice. Further reports on the development of these two compounds will be published in due course.

## 4 Experimental

### 4.1 Chemistry

Final products were analysed by reverse-phase HPLC (Alltima C18 5 µm column, 150 × 3.2 mm; Alltech Associated, Inc., Deerfield, IL) using an Agilent HP1100 equipped with a diode-array detector. Mobile phases were gradients of 80% CH_3_CN/20% H_2_O (v/v) in 45 mM NH_4_HCO_2_ at pH 3.5 and 0.5 mL/min. Purity was determined by monitoring at 330 ± 50 nm and was ≥ 95% for all final products. Melting points were determined on an Electrothermal 9100 melting point apparatus. NMR spectra were obtained on a Bruker Avance 400 spectrometer at 400 MHz for ^1^H. Low-resolution atmospheric pressure chemical ionization (APCI) mass spectra were measured for organic solutions on a ThermoFinnigan Surveyor MSQ mass spectrometer, connected to a Gilson autosampler.

#### 4.1.1 Scheme 1. New A/B units

##### 4.1.1.1 6-Bromo-3-((2,3-dihydro-1H-inden-4-yl)methyl)-2-methoxyquinoline (**AB-1**)

To a solution of 2,3-dihydro-1*H*-indene-4-carboxylic acid (6.12 g, 28.4 mmol) in THF (100 mL, dist. Na) at 0 °C was added lithium aluminium hydride (8.09 mL, 85.3 mmol) in small portions. The reaction mixture was stirred at 0 °C for 30 min and then at 20 °C for a further 24 h. The reaction mixture was washed with water (100 mL) and extracted with EtOAc (3 × 50 mL). The organic phase was dried with Na_2_SO_4_ and concentrated under reduced pressure to obtain 2,3-dihydro-1*H*-inden-4-yl)methanol (2.45 g, 99%) as a yellow oil. ^1^H NMR (CDCl_3_) *δ* 7.22–7.12 (m, 3H), 4.67 (s, 2H), 2.93 (t, *J* = 7.6 Hz, 2H), 2.91 (t, *J* = 7.4 Hz, 2H), 2.09 (p, *J* = 7.6 Hz, 2H). Found: [M + H-18] = 131.5.

To a solution of 2,3-dihydro-1*H*-inden-4-yl)methanol (4.33 g, 36.4 mmol) in DCM (50 mL) at 0 °C was added thionyl chloride (2.45 g, 16.5 mmol). The reaction mixture was stirred at 20 °C for 24 h and solvent was removed under reduced pressure. The residue was diluted with DCM (100 mL) and quenched with ice-water (100 mL). The organic phase was washed with sat. aq. NaHCO_3_, dried with Na_2_SO_4_ and concentrated to give a yellow residue. Purification by flash column chromatography using hexanes:EtOAc (1:1) gave 4-(chloromethyl)-2,3-dihydro-1*H*-indene (2.36 g, 86%) as a colourless oil. ^1^H NMR (CDCl_3_) *δ* 7.22–7.13 (m, 3H), 4.59 (s, 2H), 2.99 (t, *J* = 7.5 Hz, 2H), 2.94 (t, *J* = 7.5 Hz, 2H), 2.11 (p, *J* = 7.6 Hz, 2H). Found: [M + H] = 167.5.

A mixture of (6-bromo-2-methoxyquinolin-3-yl)boronic acid (3.69 g, 12.9 mmol), 4-(chloromethyl)-2,3-dihydro-1*H*-indene (2.36 g, 14.2 mmol) and Cs_2_CO_3_ (9.67 g, 29.7 mmol) in toluene:DMF (60 mL, 2:1) was degassed under N_2_, then Pd(PPh_3_)_4_ (0.745 g, 0.645 mmol), was added and the mixture was heated at 90 °C for 3 h. The reaction mixture was cooled to 20 °C, filtered through a plug of Celite, water (150 mL) was added the mixture was extracted with EtOAc (3 × 100 mL). The combined organic layers were washed with brine (100 mL), dried over Na_2_SO_4_, filtered and concentrated under reduced pressure to obtain a yellow residue. Purification by flash column chromatography using hexanes:EtOAc (9:1) gave 6-bromo-3-((2,3-dihydro-1H-inden-4-yl)methyl)-2-methoxyquinoline (**AB-1**) (4.70 g, 99%) as a yellow oil. ^1^H NMR (CDCl_3_) *δ* 7.71 (d, *J* = 2.2 Hz, 1H), 7.68 (d, *J* = 8.9 Hz, 1H), 7.60 (dd, *J* = 8.9, 2.2 Hz, 1H), 7.33 (s, 1H), 7.18–7.11 (m, 2H), 6.95 (d, *J* = 7.2 Hz, 1H), 4.11 (s, 3H), 3.98 (s, 2H), 2.96 (t, *J* = 7.5 Hz, 2H), 2.79 (t, *J* = 7.4 Hz, 2H), 2.04 (p, *J* = 7.6 Hz, 2H). Found: [M + H] = 368.5.

##### 4.1.1.2 6-Bromo-2-methoxy-3-((5,6,7,8-tetrahydronaphthalen-1-yl)methyl)quinolone (**AB-2**)

To a solution of 1-tetrahydronaphthoic acid (5.70 g, 32.3 mmol) in THF (100 mL, dist. Na) at 0 °C was added lithium aluminium hydride (2.46 g, 64.7 mmol) in small portions. The reaction mixture was stirred at 0 °C for 30 min and then stirred for further 24 h at 20 °C. The reaction mixture was washed with water (100 mL) and extracted with EtOAc (3 × 50 mL). The organic phase was dried with Na_2_SO_4_ and concentrated under reduced pressure to obtain (5,6,7,8-tetrahydronaphthalen-1-yl)methanol (5.23 g, 99%) as colourless oil. ^1^H NMR (CDCl_3_) *δ* 7.18 (d, *J* = 7.4 Hz, 1H), 7.11 (d, *J* = 7.5 Hz, 1H), 7.05 (d, *J* = 7.6 Hz, 1H), 4.67 (s, 2H), 2.80 (t, *J* = 6.2 Hz, 2H), 2.76 (t, *J* = 6.4 Hz, 2H), 1.88–1.77 (m, 4H). Found: [M + H-18] = 145.5.

To a solution of (5,6,7,8-tetrahydronaphthalen-1-yl)methanol (5.24 g, 32.3 mmol) in DCM (200 mL) at 0 °C was added thionyl chloride (8.45 g, 71.1 mmol). The reaction mixture was stirred at 20 °C for 24 h, then solvent was removed under reduced pressure. The residue was diluted with DCM (100 mL) and quenched with ice-water (100 mL). The organic phase was washed with sat. aq. NaHCO_3_, dried with Na_2_SO_4_ and concentrated to give 5-(chloromethyl)-1,2,3,4-tetrahydronaphthalene (3.25 g, 56%) as a brown oil. ^1^H NMR (CDCl_3_) *δ* 7.16–7.05 (m, 3H), 4.59 (s, 2H), 2.86 (t, *J* = 6.3 Hz, 2H), 2.79 (t, *J* = 6.4 Hz, 2H), 1.88–1.77 (m, 4H). Found: [M + H] = 181.6.

A mixture of (6-bromo-2-methoxyquinolin-3-yl)boronic acid (4.69 g, 16.4 mmol), 5-(chloromethyl)-1,2,3,4-tetrahydronaphthalene (3.25 g, 18.0 mmol) and Cs_2_CO_3_ (12.29 g, 37.7 mmol) in toluene:DMF (60 mL, 2:1) was degassed under N_2_, then Pd(PPh_3_)_4_ (0.948 g, 0.82 mmol) was added and the mixture was heated at 90 °C for 3 h. The reaction mixture was cooled to 20 °C, filtered through a plug of Celite, water (150 mL) was added and the mixture was extracted with EtOAc (3 × 100 mL). The combined organic layers were washed with brine (100 mL), dried over Na_2_SO_4_, filtered and concentrated under reduced pressure to obtain a yellow residue. Purification by flash column chromatography using hexanes:EtOAc (9:1) gave 6-bromo-2-methoxy-3-((5,6,7,8-tetrahydronaphthalen-1-yl)methyl)quinolone (**AB-2**) (4.64 g, 74%) as a white solid. ^1^H NMR (CDCl_3_) *δ* 7.71–7.69 (m, 2H), 7.60 (dd, *J* = 8.9, 2.2 Hz, 1H), 7.23 (s, 1H), 7.12–7.04 (m, 2H), 6.92 (d, *J* = 6.9 Hz, 1H), 4.11 (s, 3H), 3.96 (s, 2H), 2.83 (bs, 2H), 2.57 (bs, 2H), 1.76 (p, *J* = 3.5 Hz, 4H). Found: [M + H] = 382.1.

##### 4.1.1.3 6-Bromo-3-(2-fluoro-3-methylbenzyl)-2-methoxyquinoline (**AB-3**)

A mixture of (6-bromo-2-methoxyquinolin-3-yl)boronic acid (3.00 g, 10.5 mmol), 1-(bromomethyl)-2-fluoro-3-methylbenzene (**S7**) (4.24 g, 20.9 mmol) and Cs_2_CO_3_ (7.87 g, 24.2 mmol) in toluene:DMF (60 mL, 2:1) was degassed under N_2_, then Pd(PPh_3_)_4_ (0.607 g, 0.525 mmol) was added, and the mixture was heated at 90 °C for 2 h. The reaction mixture was cooled to 20 °C, filtered through a plug of Celite, water (150 mL) was added and the mixture was extracted with EtOAc (3 × 100 mL). The organic layer was washed with brine (100 mL), dried over Na_2_SO_4_, filtered and concentrated under reduced pressure to obtain a yellow residue. Purification by flash column chromatography using hexanes:EtOAc (9:1) gave *6-*bromo-3-(2-fluoro-3-methylbenzyl)-2-methoxyquinoline (**AB-3**) (2.87 g, 76%) as a white solid. ^1^H NMR (CDCl_3_) *δ* 7.75 (d, *J* = 2.2 Hz, 1H), 7.67 (d, *J* = 8.8 Hz, 1H), 7.60 (dd, *J* = 8.9, 2.2 Hz, 1H), 7.50 (s, 1H), 7.11–6.96 (m, 3H), 4.09 (s, 3H), 4.03 (s, 2H), 2.28 (d, *J* = 2.1 Hz, 3H). Found: [M + H] = 360.6.

##### 4.1.1.4 6-Bromo-3-(3-fluoro-4-methoxybenzyl)-2-methoxyquinoline (**AB-4**)

Borane − dimethylsulfide complex (2.79 mL, 29.40 mmol) and trimethyl borate (3.34 mL, 29.40 mmol) were added to a solution of 3-fluoro-4-methoxybenzoic acid (2.50 g, 14.69 mmol) in THF (80 mL, dist. Na) at 0 °C, and the solution warmed to 20 °C and stirred overnight. The mixture was then cooled to 0 °C, and quenched with MeOH (10 mL). The solvent was then evaporated and the residue was partitioned between EtOAc and water. The organic layer was then dried and evaporated to afford (3-fluoro-4-methoxyphenyl)methanol, (2.35 g, 100%). ^1^H NMR (CDCl_3_) *δ* 7.11 (1H, dd, *J* = 2.0, 11.9 Hz), 7.06 (1H, ddd, *J* = 0.9, 2.0, 9.1 Hz), 6.94 (1H, dd, *J* = 8.4, 8.4 Hz), 4.61 (2H, s), 3.89 (3H, s), 1.73 (1H, s). Found: [M−OH] = 139.7.

To a solution of (3-fluoro-4-methoxyphenyl)methanol (2.35 g, 15.05 mmol) and triethylamine (3.15 mL, 22.58 mmol) in DCM (50 mL, anhydrous) at 20 °C was added mesyl chloride (1.414 mL, 18.06 mmol) dropwise. After 15 min, the reaction was diluted with DCM (50 mL) and the organic layer washed with sat. aq. NaHCO_3_, dried and evaporated. The residue was dissolved in acetone (100 mL, anhydrous), lithium bromide (excess) added, and the mixture heated at reflux for 30 min. The solution was then cooled and the solvent evaporated, and the residue partitioned between EtOAc and water. The aqueous layer was extracted twice with EtOAc and the organic layer was dried and evaporated to afford 4-(bromomethyl)-2-fluoro-1-methoxybenzene (3.00 g, 91%). ^1^H NMR (CDCl_3_) *δ* 7.16–7.07 (2H, m), 6.90 (1H, dd, *J* = 8.3, 8.4 Hz), 4.45 (2H, s), 3.89 (3H, s).

A mixture of (6-bromo-2-methoxyquinolin-3-yl)boronic acid (3.51 g, 12.45 mmol), 4-(bromomethyl)-2-fluoro-1-methoxybenzene (3.00 g, 13.69 mmol) and cesium carbonate (8.93 g, 27.40 mmol) in toluene (60 mL, anhydrous) and DMF (30 mL, anhydrous) was purged with nitrogen. Pd(PPh_3_)_4_ (0.58 g, 0.50 mmol) was then added, the mixture purged with nitrogen then heated to 80 °C under nitrogen for 5 h. The reaction was partitioned between EtOAc and water and the organic fraction was dried and evaporated. Column chromatography (19:1 hexanes/EtOAc) gave 6-bromo-3-(3-fluoro-4-methoxybenzyl)-2-methoxyquinoline (**AB-4**) (2.10 g, 41%). ^1^H NMR (CDCl_3_) *δ* 7.76 (1H, d, *J* = 2.2 Hz), 7.69 (1H, d, *J* = 12.3 Hz), 7.62 (1H, dd, *J* = 2.2, 8.9 Hz), 7.49 (1H, s), 7.00–6.86 (3H, m), 4.07 (3H, s), 3.95 (2H, s), 3.88 (3H, s). Found: [M + H] = 376.0

##### 4.1.1.5 3-(Benzo[d][1,3]dioxol-4-ylmethyl)-6-bromo-2-methoxyquinoline (**AB-5**)

A mixture of **(**6-bromo-2-methoxyquinolin-3-yl)boronic acid (1.5 g, 5.32 mmol), 4-(bromomethyl)benzo[d][1,3]dioxole (1.2 g, 5.59 mmol) and 2 M Na_2_CO_3_ (5 mL) in DME (25 mL) was purged with nitrogen. Pd(PPh_3_)_4_ (0.31 g, 0.27 mmol) was added, the mixture was purged with nitrogen then heated to 95 °C under nitrogen for 4 h. The reaction was partitioned between EtOAc and water and the organic fraction was dried and evaporated. Column chromatography with hexanes:EtOAc (100:0 to 95:5) gave 3-(benzo[d][1,3]dioxol-4-ylmethyl)-6-bromo-2-methoxyquinoline (**AB-5**) (1.52 g, 76%). ^1^H NMR (CDCl_3_) *δ* 7.77 (d, *J* = 2.2 Hz, 1H), 7.69 (d, *J* = 8.9 Hz, 1H), 7.61 (dd, *J* = 8.9, 2.2 Hz, 1H), 7.53 (s, 1H) , 6.82–6.73 (m, 2H), 6.70 (dd, *J* = 7.4, 1.7 Hz, 1H), 5.94 (s, 2H), 4.09 (s, 3H), 3.98 (s, 2H). Found: [M + H] = 373.1

##### 4.1.1.6 3-(Benzofuran-7-ylmethyl)-6-bromo-2-methoxyquinoline (**AB-6**)

A solution of methyl benzofuran-7-carboxylate(3.59 g, 20.4 mmol) in Et_2_O (100 mL, dist. Na) at 0 °C was treated with LiAlH_4_ (1.54 g, 40.6 mmol) then stirred at 20 °C for 3 h and quenched with ice. The mixture was partitioned between Et_2_O and sat. aq. sodium potassium tartrate and then filtered through Celite. The aqueous layer was extracted with Et_2_O and the organic phases were combined and dried. Column chromatography (0–5% EtOAc:DCM) gave benzofuran-7-ylmethanol (2.60 g, 86%). ^1^H NMR (CDCl_3_) *δ* 7.65 (d, *J* = 2.2 Hz, 1H), 7.56 (dd, *J* = 7.7, 1.2 Hz, 1H), 7.31 (dd, *J* = 7.3, 0.6 Hz, 1H), 7.23 (t, *J* = 7.5 Hz, 1H), 6.80 (d, *J* = 2.2 Hz, 1H), 5.02 (d, *J* = 6.2 Hz, 2H), 1.93 (t, *J* = 6.2 Hz, 1H).

A solution of benzofuran-7-ylmethanol (4.72 g, 31.8 mmol) in DCM (100 mL, anhydrous) at 0 °C was treated sequentially with triethylamine (8.9 mL, 63.9 mmol) then mesyl chloride (3.70 mL, 47.8 mmol), the mixture was stirred at 0 °C for 1 h then partitioned between DCM and water. The organic fraction was dried and evaporated and the residue was dissolved in acetone (200 mL), LiBr (27.6 g, 318 mmol) was added and the mixture was refluxed for 0.5 h and then evaporated. The residue was partitioned between DCM and water; the organic fraction was dried and evaporated. Column chromatography (DCM) gave 7-(bromomethyl)benzofuran (6.08 g, 90%). ^1^H NMR (CDCl_3_) *δ* 7.69 (d, *J* = 2.2 Hz, 1H), 7.57 (dd, *J* = 7.7, 1.2 Hz, 1H), 7.32 (dd, *J* = 7.4, 0.7 Hz, 1H), 7.22 (t, *J* = 7.6 Hz, 1H), 6.80 (d, *J* = 2.2 Hz, 1H), 4.81 (s, 2H).

A mixture of **(**6-bromo-2-methoxyquinolin-3-yl)boronic acid (8.00 g, 28.4 mmol), 7-(bromomethyl)benzofuran (5.99 g, 28.4 mmol) and Cs_2_CO_3_ (18.5 g, 56.7 mmol) in toluene (100 mL) and DMF (50 mL) was purged with nitrogen. Pd(PPh_3_)_4_ (0.66 g, 0.57 mmol) was added, the mixture was purged with nitrogen then heated to 80 °C under nitrogen for 3 h. The reaction was partitioned between EtOAc and water and the organic fraction was dried and evaporated. Column chromatography with 3:1 hexanes:DCM eluted impurities, then elution with 1:1 hexanes:DCM then DCM gave 3-(benzofuran-7-ylmethyl)-6-bromo-2-methoxyquinoline (**AB-6**) (6.95 g, 67%). ^1^H NMR (CDCl_3_) *δ* 7.71 (d, *J* = 2.2 Hz, 1H), 7.68 (d, *J* = 8.8 Hz, 1H), 7.58–7.62 (m, 2H), 7.50–7.54 (m, 2H) , 7.20 (t, *J* = 7.4 Hz, 1H), 7.13 (dd, *J* = 7.4, 0.6 Hz, 1H), 6.79 (d, *J* = 2.2 Hz, 1H), 4.32 (s, 2H), 4.10 (s, 3H). Found: [M + H] = 368.8

##### 4.1.1.7 5-((6-Bromo-2-methoxyquinolin-3-yl)methyl)-N,*N*-diethylpyridin-2-amine (**AB-7**)

To a solution of freshly distilled *N*,*N*,*N*,*N*,-tetramethylpiperidine (1.60 mL, 9.56 mmol) in freshly distilled THF (13 mL) was added at −30 °C under nitrogen , *n*-BuLi (4.40 mL, 8.76 mmol) dropwise. The mixture was maintained at about −30 °C for 15 min, then cooled to −78 °C. A solution of 6-bromo-2-methoxyquinoline (1.90 g, 7.96 mmol) in dry THF (15 mL) was added dropwise at −78 °C. The resultant organic mixture was stirred at the same temperature for 75 min. A solution of 6-(diethylamino)nicotinaldehyde (1.42 g, 7.96 mmol) in dry THF (6 mL) was added dropwise at −78 °C, the reaction mixture remained orange brown, stirred at −78 °C for 2.5 h. The mixture was quenched with acetic acid (0.68 mL) at −65 °C. Water was added, the aqueous mixture was extracted with ethyl acetate (2x), and the combined extract was washed with brine, dried (MgSO_4_) and concentrated in *vacuo* to give the crude product as a yellow solid. Flash chromatography of the crude product using 10–100% ethyl acetate in hexane as eluent afforded product (6-bromo-2-methoxyquinolin-3-yl)(6-(diethylamino)pyridin-3-yl)methanol (1.90 g, 57%) as an off-white solid. ^1^H NMR (CDCl_3_) *δ* 8.15 (d, *J* = 2.4 Hz, 1H), 8.01 (s, 1H), 7.88 (d, *J* = 2.1 Hz, 1H), 7.70–7.64 (m, 2H), 7.39 (dd, *J* = 8.9, 2.5 Hz, 1H), 6.43 (d, *J* = 8.9 Hz, 1H), 5.94 (d, *J* = 3.7 Hz, 1H), 4.04 (s, 3H), 3.53–3.47 (m, 4H), 2.65 (d, *J* = 3.9 Hz, 1H), 1.17 (t, *J* = 7.0 Hz, 6H).

To a sparingly soluble solution of 2-methoxyquinolin-3-yl)(6-(diethylamino)pyridin-3-yl)methanol (1.90 g, 4.57 mmol) in freshly distilled THF (19 mL) was added at 2 °C under nitrogen sodium borohydride (0.86 g, 23.0 mmol) in 3 portions over 10 min. The mixture was stirred at 2–4 °C for 1 h. The mixture was cooled to 2 °C again, aluminium chloride (1.83 g, 13.7 mmol) was added in 4 batches over 15 min. The mixture was stirred at 2 °C for 10 min, then refluxed for 2 h. The mixture was then quenched with water cautiously at 2 °C, until gas evolution ceased. The white slurry was filtered through celite. The milky white filtrate was diluted in water, and the organic phase was collected. The aqueous phase was extracted with ethyl acetate (3x). The organic extract was washed with brine, dried (MgSO_4_) and concentrated in *vacuo* to furnish the crude product as a brownish residue. Flash chromatography of the crude product using 10% ethyl acetate in hexane as eluent afforded product 5-((6-bromo-2-methoxyquinolin-3-yl)methyl)-*N*,*N*-diethylpyridin-2-amine (**AB-7**) (1.39 g, 76%) as a white solid. ^1^H NMR (CDCl_3_) *δ* 8.06 (d, *J* = 2.1 Hz, 1H), 7.76 (d, *J* = 2.2 Hz, 1H), 7.67 (d, *J* = 8.8 Hz, 1H), 7.60 (dd, *J* = 8.8, 2.2 Hz, 1H), 7.52 (s, 1H), 7.28 (dd, *J* = 8.7, 2.4 Hz, 1H), 6.42 (dd, *J* = 8.7, 0.3 Hz, 1H), 4.09 (s, 3H), 3.85 (s, 2H), 3.50 (q, *J* = 7.0 Hz, 4H), 1.18 (t, *J* = 7.0 Hz, 6H).

##### 4.1.1.8 6-Bromo-3-((5-isopropoxy-2-methoxypyridin-3-yl)methyl)-2-methoxyquinoline (**AB-8**)

A mixture of 5-hydroxy-2-methoxynicotinaldehyde (1.00 g, 6.53 mmol) and potassium carbonate (1.35 g, 9.80 mmol) in DMF (30 mL, anhydrous) was heated at 50 °C for 10 min. Isopropyl iodide (0.78 mL, 7.84 mmol) was then added and the mixture stirred at this temperature for 2 h. The resultant solution was diluted with EtOAc and washed with brine three times. The organic layer was dried and evaporated to afford 5-isopropoxy-2-methoxynicotinaldehyde (0.90 g, 71%). ^1^H NMR (CDCl_3_) *δ* 10.34 (1H, s), 8.07 (1H, d, *J* = 3.2 Hz), 7.66 (1H, d, *J* = 3.2 Hz), 4.48 (1H, sp, *J* = 6.1 Hz), 4.03 (3H, s), 1.33 (6H, d, *J* = 4.8 Hz).

A mixture of isopropoxy-2-methoxynicotinaldehyde (0.90 g, 4.61 mmol) and sodium borohydride (0.35 g, 9.22 mmol) in MeOH (15 mL, anhydrous) was stirred at 20 °C for 1 h. The solvent was then removed and the residue partitioned between EtOAc and water. The organic layer was dried and evaporated. Column chromatography with 9:1 hexanes/EtOAc afforded (5-isopropoxy-2-methoxypyridin-3-yl)methanol (0.68 g, 75%). ^1^H NMR (CDCl_3_) *δ* 7.72 (1H, s), 7.25 (1H, d, *J* = 3 Hz), 4.61 (2H, s), 4.42 (1H, sp), 3.93 (3H, s), 2.92–2.19 (1H, br s), 1.32 (6H, d, *J* = 6.1 Hz).

To a solution of (5-isopropoxy-2-methoxypyridin-3-yl)methanol (0.68 g, 3.45 mmol) and triethylamine (0.72 mL, 5.18 mmol) in DCM (10 mL, anhydrous) at 20 °C was added mesyl chloride (0.32 mL, 4.14 mmol) dropwise. After 15 min, the reaction was diluted with DCM (10 mL) and the organic layer washed with sat. NaHCO_3_, dried and evaporated. The residue was redissolved in acetone (20 mL, anhydrous), lithium bromide (excess) added, and the mixture heated at reflux for 30 min. The solution was then cooled and the solvent evaporated, and the residue partitioned between EtOAc and water. The aqueous layer was extracted twice with EtOAc and the organic layer was dried and evaporated to afford 3-(bromomethyl)-5-isopropoxy-2-methoxypyridine (0.70 g, 78%). ^1^H NMR (CDCl_3_) *δ* 7.76 (1H, dd, *J* = 2.6, 3.4 Hz), 7.25 (1H, dd, *J* = 2.4, 2.4 Hz), 4.5 (2H, d, *J* = 2.2 Hz), 4.42 (1H, sp, *J* = 2.6, 6.0 Hz), 3.96 (3H, d, *J* = 2.9 Hz), 1.32 (6H, dd, *J* = 3.0, 6.1 Hz).

A mixture of (6-bromo-2-methoxyquinolin-3-yl)boronic acid (0.80 g, 2.82 mmol), 3-(bromomethyl)-5-isopropoxy-2-methoxypyridine (0.70 g, 2.69 mmol) and cesium carbonate (1.75 g, 5.38 mmol) in toluene (10 mL, anhydrous) and DMF (5 mL, anhydrous) was purged with nitrogen. Pd(PPh_3_)_4_ (0.12 g, 0.11 mmol) was then added, and the mixture then heated to 80 °C under nitrogen for 4 h. The reaction was partitioned between EtOAc and water and the organic fraction was dried and evaporated. Column chromatography (19:1 hexanes/EtOAc) gave 6-bromo-3-((5-isopropoxy-2-methoxypyridin-3-yl)methyl)-2-methoxyquinoline (**AB-8**) (0.57 g, 48%). ^1^H NMR (CDCl_3_) *δ* 7.77 (1H, d, *J* = 2.2 Hz), 7.71 (1H, d, *J* = 2.9 Hz), 7.69 (1H, d, *J* = 8.9 Hz), 7.62 (1H, dd, *J* = 2.2, 8.9 Hz), 7.56 (1H, s), 7.02 (1H, d, *J* = 2.9 Hz), 4.38 (1H, sp, *J* = 6.0 Hz), 4.08 (3H, s), 3.92 (2H, s), 3.90 (3H, s), 1.29 (6H, d, *J* = 6.1 Hz).

##### 4.1.1.9 6-Bromo-2-methoxy-3-((2,4,5-trimethoxypyridin-3-yl)methyl)quinolone (**AB-9**)

To a mixture of 2,6-dimethoxypyridin-3-ol (8.05 g, 51.9 mmol) and imidazole (7.42 g, 108.99 mmol) in DMF (130 mL) at 20 °C was added triisopropylsilyl chloride (13.33 mL, 62.23 mmol), and the resultant mixture stirred at 20 °C for 2 h. The solution was then partitioned between EtOAc and water, and the aqueous layer extracted three times. The combined organic layers were washed with brine three times, dried and evaporated. Column chromatography with 19:1 hexanes/EtOAc afforded the product 2,6-dimethoxy-3-((triisopropylsilyl)oxy)pyridine (15.59 g, 96%). ^1^H NMR (CDCl_3_) *δ* 7.07 (1H, d, *J* = 8.2 Hz), 6.13 (1H, d, *J* = 8.2 Hz), 3.92 (3H, s), 3.86 (3H, s), 1.27–1.18 (3H, m), 1.08 (18H, d, *J* = 7.1 Hz). Found: [M + H] = 312.8.

To a solution of 2,6-dimethoxy-3-((triisopropylsilyl)oxy)pyridine (8.00 g, 25.69 mmol) and *N*,*N*-diisopropylamine (0.18 mL, 1.28 mmol) in THF (100 mL, dist. Na) at −40 °C under nitrogen was added *n*-BuLi (15.41 mL, 30.83 mmol) dropwise. The resultant solution was stirred at −40 °C for 5 min, and then warmed to 0 °C and stirred at this temperature for a further 3 h. The solution was then again cooled to −40 °C, and formylpiperidine (4.28 mL, 38.54 mmol) was added dropwise, and the mixture stirred at 20 °C for another 1 h. Acetic acid (8 mL) was added and the solvent was removed in *vacuo*. The resultant mixture was partitioned between EtOAc and water, and the organic fraction dried and evaporated. Column chromatography with 49:1 hexanes/EtOAc afforded the product 2,6-dimethoxy-5-((triisopropylsilyl)oxy)nicotinaldehyde (7.55 g, 87%). ^1^H NMR (CDCl_3_) *δ* 10.17 (1H, s), 7.51 (1H, s), 4.02 (3H, s), 4.01 (3H, s), 1.30–1.19 (3H, m), 1.08 (18H, d, *J* = 7.3 Hz). Found: [M−CHO]^+^= 312.8.

Tetrabutylammonium fluoride in THF (1 N, 33.36 mL, 33.36 mmol) was added to a solution of 2,6-dimethoxy-5-((triisopropylsilyl)oxy)nicotinaldehyde (7.55 g, 22.24 mmol) in THF (35 mL, dist. Na) at 0 °C. The reaction was then warmed to 20 °C and stirred for 4 h. The solvent was removed and the residue partitioned between EtOAc and water. The aqueous layer was extracted with EtOAc three times, and the organic layer dried and evaporated. Column chromatography with DCM followed by 3:1 DCM/EtOAc afforded the product 5-hydroxy-2,6-dimethoxynicotinaldehyde (3.15 g, 77%). ^1^H NMR (CDCl_3_) *δ* 10.20 (1H, s), 7.59 (1H, s), 5.15–4.80 (1H, br s), 4.10 (3H, s), 4.01 (3H, s).

A mixture of 5-hydroxy-2,6-dimethoxynicotinaldehyde (3.15 g, 17.20 mmol) and potassium carbonate (3.57 g, 25.80 mmol) in DMF (80 mL, anhydrous) was heated at 50 °C for 10 min. Methyl iodide (1.29 mL, 20.64 mmol) was then added and the mixture stirred at this temperature for 2 h. The resultant solution was diluted with EtOAc and washed with brine three times. The organic layer was dried and evaporated to afford the product 2,5,6-trimethoxynicotinaldehyde (3.39 g, 100%). ^1^H NMR (CDCl_3_) *δ* 10.21 (1H, s), 7.53 (1H, s), 4.10 (3H, s), 4.02 (3H, s), 3.87 (3H, s).

A solution of *N*,*N*,*N*,*N*-tetramethylpiperidine (3.59 mL, 21.03 mmol) in THF (40 mL, dist. Na) was cooled to −40 °C, *n*-BuLi (10.52 mL, 21.03 mmol) was added and the solution was stirred at −40 °C for 15 min, then cooled to −78 °C. A solution of 6-bromo-2-methoxyquinoline (17.53 mmol) in THF (40 mL, dist. Na) was added dropwise, the orange solution was stirred at −78 °C for 1.5 h, then a solution of 2,5,6-trimethoxynicotinaldehyde (3.42 g, 17.53 mmol) in THF (40 mL, dist. Na) was added. The mixture was stirred at −78 °C for 2 h, then acetic acid (2.5 mL) was added and the solution was allowed to warm to 20 °C. The solvent was removed and the residue partitioned between EtOAc and water, and the organic fraction was dried and evaporated. Column chromatography with 9:1 hexanes/EtOAc followed by 4:1 hexanes/EtOAc gave the product (6-bromo-2-methoxyquinolin-3-yl)(2,5,6-trimethoxypyridin-3-yl)methanol (5.50 g, 72%) as a white solid. ^1^H NMR (CDCl_3_) *δ* 7.85 (1H, d, *J* = 2.1 Hz), 7.79 (1H, s), 7.70 (1H, d, *J* = 8.9 Hz), 7.65 (1H, dd, *J* = 2.1, 8.9 Hz), 7.15 (1H, s), 6.14 (1H, d, *J* = 5.2 Hz), 4.08 (3H, s), 4.02 (3H, s), 3.90 (3H, s), 3.78 (3H, s), 3.48 (1H, d, *J* = 5.4 Hz). Found: [M + H] = 436.1

Trifluoroacetic acid (11.30 mL, 148.2 mmol) and triethylsilane (17.76 mL, 111.2 mmol) were added sequentially to a solution of (6-bromo-2-methoxyquinolin-3-yl)(2,5,6-trimethoxypyridin-3-yl)methanol (5.35 g, 12.35 mmol) in DCM (125 mL) and the solution was stirred for 1 h at 20 °C, then ice water was added. The solution was partitioned between sat. aq. NaHCO_3_ and DCM and the aqueous fraction was extracted with DCM. The organic fractions were combined, dried and evaporated. Column chromatography with 9:1 hexanes/EtOAc gave 6-bromo-2-methoxy-3-((2,4,5-trimethoxypyridin-3-yl)methyl)quinolone (**AB-9**) (4.45 g, 86%) as a white solid. ^1^H NMR (CDCl_3_) *δ* 7.76 (1H, d, *J* = 2.2 Hz), 7.68 (1H, d, *J* = 8.9 Hz), 7.60 (1H, dd, *J* = 2.2, 8.4 Hz), 7.49 (1H, s), 7.05 (1H, s), 4.09 (3H, s), 4.01 (3H, s), 3.89 (5H, s), 3.79 (3H, s). Found: [M + H] = 419.0

##### 4.1.1.10 6-Bromo-3-((2,5-dimethoxypyridin-4-yl)methyl)-2-methoxyquinoline (**AB-10**)

To a solution of 6-methoxypyridin-3-ol (3.20 g, 25.57 mmol) in E (50 mL, anhydrous) at 0 °C was added sodium hydride (60% in mineral dispersion, 1.23 g, 30.69 mmol) in portions. The mixture was warmed to 20 °C and stirred for 1 h. Chloromethyl ethyl ether (2.73 mL, 29.41 mmol) was then added, and the resultant mixture stirred at 20 °C for a further 2 h. The reaction was diluted with water and extracted with EtOAc (x3). The organic layer was washed with brine (x3), dried and evaporated. Column chromatography with 9:1 X4:EtOAc afforded 5-(ethoxymethoxy)-2-methoxypyridine (4.09 g, 87%). ^1^H NMR (CDCl_3_) *δ* 7.95 (d, *J* = 3.0 Hz, 1H), 7.34–7.31 (m, 1H), 6.67 (d, *J* = 9.0 Hz, 1H), 5.13 (s, 2H), 3.88 (s, 3H), 3.72 (q, *J* = 7.1 Hz, 2H), 1.22 (t, *J* = 7.0 Hz, 3H). Found: [M + H] = 184.4.

To a solution of 5-(ethoxymethoxy)-2-methoxypyridine (6.20 g, 33.84 mmol) and diisopropylamine (0.24 mL, 1.69 mmol) in THF (100 mL, dist. Na) at −40 °C under nitrogen was added *n*-BuLi (25.4 mL, 50.76 mmol) dropwise. The resultant solution was stirred at −40 °C for 5 min, and then warmed to 0 °C and stirred at this temperature for a further 3 h. The solution was then again cooled to −40 °C, and *n*-formylpiperidine (6.76 mL, 60.91 mmol) was added dropwise, and the mixture stirred at 20 °C for another 1 h. Acetic acid (15 mL) was added and the solvent was removed in *vacuo*. The resultant mixture was partitioned between EtOAc and water, and the organic fraction dried and evaporated. Column chromatography with 9:1 X4:EtOAc afforded 5-(ethoxymethoxy)-2-methoxyisonicotinaldehyde (4.00 g, 56%). ^1^H NMR (CDCl_3_) *δ* 10.43 (s, 1H), 8.27 (s, 1H), 7.07 (d, *J* = 0.4 Hz, 1H), 5.30 (s, 2H), 3.92 (s, 3H), 3.78 (q, *J* = 7.1 Hz, 2H), 1.26 (t, *J* = 7.1 Hz, 3H).

A solution of 5-(ethoxymethoxy)-2-methoxyisonicotinaldehyde (4.00 g, 18.85 mmol) and 3 M HCl (60 mL) in THF (40 mL, dist. Na) was heated at 40 °C for 3 h. The solution was then cooled, diluted with water, and the pH adjusted to 7 using potassium carbonate. The aqueous layer was then extracted with EtOAc three times, and the organic layer dried and evaporated. Column chromatography with 9:1 X4:EtOAc afforded 5-hydroxy-2-methoxyisonicotinaldehyde (2.50 g, 87%). ^1^H NMR (CDCl_3_) *δ* 9.97 (d, *J* = 0.7 Hz, 1H), 9.46 (s, 1H), 8.08 (s, 1H), 6.93 (d, *J* = 0.6 Hz, 1H), 3.94 (s, 3H).

A mixture of 5-hydroxy-2-methoxyisonicotinaldehyde (2.50 g, 16.33 mmol) and potassium carbonate (3.39 g, 24.45 mmol) in DMF (80 mL, anhydrous) was heated at 50 °C for 10 min. Methyl iodide (1.22 mL, 19.59 mmol) was then added and the mixture stirred at this temperature for 2 h. The resultant solution was diluted with EtOAc and washed with brine three times. The organic layer was dried and evaporated to afford 2,5-dimethoxyisonicotinaldehyde (2.25 g, 82%). ^1^H NMR (CDCl_3_) *δ* 10.4 (s, 1H), 8.01 (s, 1H), 7.07 (s, 1H), 3.97 (s, 3H), 3.91 (s, 3H). Found [M + MeOH] = 200.4

A mixture of 2,5-dimethoxyisonicotinaldehyde (2.25 g, 13.46 mmol) and sodium borohydride (1.02 g, 26.92 mmol) in MeOH (50 mL, anhydrous) was stirred at 20 °C for 1 h. The solvent was then removed and the residue partitioned between EtOAc and water. The organic layer was dried and evaporated to afford (2,5-dimethoxypyridin-4-yl)methanol (2.17 g, 95%). ^1^H NMR (CDCl_3_) *δ* 7.70 (s, 1H), 6.77 (s, 1H), 4.66 (d, *J* = 6.0 Hz, 2H), 3.89 (s, 3H), 3.87 (s, 3H), 2.23 (t, *J* = 6.4 Hz, 1H).

To a solution of (2,5-dimethoxypyridin-4-yl)methanol (2.05 g, 12.12 mmol) and triehylamine (2.53 mL, 18.18 mmol) in DCM (35 mL, anhydrous) at 20 °C was added methanesulfonyl chloride (1.13 mL, 14.50 mmol) dropwise. After 15 min, the reaction was diluted with DCM (20 mL) and the organic layer washed with sat. sodium hydrogen carbonate, dried and evaporated. The residue was redissolved in acetone (70 mL, anhydrous), Lithium bromide (10 g, excess) added, and the mixture heated at reflux for 30 min. The solution was then cooled and the solvent evaporated, and the residue partitioned between EtOAc and water. The aqueous layer was extracted twice with EtOAC and the organic layer was dried and evaporated to afford 4-(bromomethyl)-2,5-dimethoxypyridine (2.54 g, 90%). ^1^H NMR (CDCl_3_) *δ* 7.75 (s, 1H), 6.75 (s, 1H), 4.40 (s, 2H), 3.91 (s, 3H), 3.88 (s, 3H).

A mixture of (6-bromo-2-methoxyquinolin-3-yl)boronic acid (2.80 g, 9.95 mmol), 4-(bromomethyl)-2,5-dimethoxypyridine (2.54 g, 10.94 mmol) and cesium carbonate (6.50 g, 19.9 mmol) in toluene (50 mL, anhydrous) and DMF (25 mL, anhydrous) was purged with nitrogen. Pd(PPh_3_)_4_ (0.046 g, 0.40 mmol) was then added, the mixture purged with nitrogen then heated to 80 °C under nitrogen for 4 h. The reaction was partitioned between EtOAc and water and the organic fraction was dried and evaporated. Column chromatography (19:1 x4:EtOAc) gave 6-bromo-3-((2,5-dimethoxypyridin-4-yl)methyl)-2-methoxyquinoline (**AB-10**) (2.20 g, 51%). ^1^H NMR (CDCl_3_) *δ* 7.77 (d, *J* = 2.2 Hz, 1H), 7.75 (s, 1H), 7.69 (d, *J* = 8.9 Hz, 1H), 7.62 (dd, *J* = 8.9, 2.2 Hz, 1H), 7.53 (s, 1H), 6.48 (s, 1H), 4.07 (s, 3H), 3.97 (s, 2H), 3.86 (s, 3H), 3.83 (s, 3H). Found: [M + H] = 389.7.

##### 4.1.1.11 3-((2,6-Bis(methylthio)pyridin-4-yl)methyl)-6-bromo-2-methoxyquinoline (**AB-11**)

2,6-Dichloroisonicotinic acid (4.00 g, 20.8 mmol) in DMF (40 mL) was added sodium thiomethoxide (4.38 g, 65.5 mmol) at 0 °C in batches. Mixture was stirred at 150 °C for 18 h. Water (40 mL) was added to the resultant solution and pH was adjusted to 3 using 2 M HCl solution. Extracted with EtOAc (3x), dried with MgSO_4_, filtered and the solvent was evaporated to give 2,6-bis(methylthio)isonicotinic acid (4.01 g, 90%) as an orange solid. The solid was recrystallized from methanol and was used without further purification for the next step. ^1^H NMR (CDCl_3_) *δ* 7.44 (s, 2H), 2.61 (s, 6H). Found: [M + H] = 216.5.

Borane dimethyl sulfide complex (8.09 mL, 85.3 mmol) and trimethyl borate (9.68 mL, 85.3 mmol) were added to a solution of 2,6-bis(methylthio)isonicotinic acid (6.12 g, 28.4 mmol) in THF (100 mL, dist. Na) at 0 °C. The solution was then warmed to room temperature and was stirred for 21 h at 20 °C. The reaction mixture was cooled to 0 °C, MeOH (20 mL) was added to quench the reaction and the solvent was evaporated in the fume hood. The residue was partitioned between EtOAc and water, extracted with EtOAc (3x), dried with MgSO_4_, filtered and the solvent was evaporated. Column chromatography (4:1 hexanes:EtOAc) gave (2,6-bis(methylthio)pyridin-4-yl)methanol (4.56 g, 80%) as a white solid. M.p. 88 – 90 °C. ^1^H NMR (CDCl_3_) *δ* 6.87 (s, 2H), 4.61 (s, 2H), 2.58 (s, 6H). Found: [M + H] = 202.5.

Phosphorus tribromide (2.97 mL, 31.4 mmol) was added to a solution of (2,6-bis(methylthio)pyridin-4-yl)methanol (5.26 g, 26.1 mmol) in DCM (300 mL) at 0 °C. The reaction mixture was stirred at 20 °C for 18 h then solvent was evaporated. The residue was diluted with DCM (100 mL) and quenched with ice, the organic layer was washed with sat. aq. NaHCO_3_, dried with MgSO_4_, filtered and the solvent was evaporated. Column chromatography (6:1 hexanes:EtOAc) gave 4-(bromomethyl)-2,6-bis(methylthio)pyridine (3.44 g, 50%) as a white solid. M.p. 100 – 102 °C. ^1^H NMR (CDCl_3_) *δ* 6.86 (s, 2H), 4.23 (s, 2H), 2.58 (s, 6H). Found: [M + H] = 266.4.

A mixture of 4-(bromomethyl)-2,6-bis(methylthio)pyridine (3.44 g, 15.0 mmol), (6-bromo-2-methoxyquinolin-3-yl)boronic acid (4.31 g, 15.0 mmol), and Cs_2_CO_3_ (11.24 g, 34.5 mmol) in toluene-DMF (60 mL, 2:1) was degassed under N_2_, then added Pd(PPh_3_)_4_ (0.867 g, 0.750 mmol), and heated at 90 °C for 2 h. Reaction mixture was cooled to 20 °C, filtered through a plug of celite, added water and extracted with EtOAc (x4). Organic layer washed with brine, dried with Na_2_SO_4_, filtered and the solvent was evaporated to give a yellow residue. Purification by flash column chromatography with silica using hexane:EtOAc (9:1) to gave 3-((2,6-bis(methylthio)pyridin-4-yl)methyl)-6-bromo-2-methoxyquinoline (**AB-11**) (2.90 g, 46%) as a white solid. M.p. 129 – 131 °C. ^1^H NMR (CDCl_3_) *δ* 7.80 (d, *J* = 2.1 Hz, 1H), 7.70 (d, *J* = 8.9 Hz, 1H), 7.65 (dd, *J* = 8.9, 2.2 Hz), 7.57 (s, 1H), 6.72 (s, 2H), 4.05 (s, 3H), 3.87 (s, 2H), 2.57 (s, 6H). Found: [M + H] = 421.8.

##### 4.1.1.12 3-((2,6-Bis(ethylthio)pyridin-4-yl)methyl)-6-bromo-2-methoxyquinoline (**AB-12**)

Sodium hydride (60% w/w, 1.44 g, 36.1 mmol) in DMF (25 mL) at 0 °C was added ethanethiol (2.70 mL, 36.1 mmol) dropwise. The mixture foamed up as hydrogen gas was released. The mixture was stirred at 0 °C for 15 min, then a solution of 2,6-dichloroisonicotinic acid (2.05 g, 11.6 mmol) in anhydrous DMF (9 mL) was added dropwise at 0 °C. The mixture was then stirred at 150 °C for 17 h. The mixture was diluted in water, dissolving the white salts. The aqueous mixture was purged with air to remove most of the stench. 2 M hydrochloric acid was added until pH reached ∼ 3, when white solids crashed out. The solids were collected by filtration, washed with ice-cold water, dried to yield 2,6-bis(ethylthio)isonicotinic acid (2.82 g, 100%) as light yellow solid which was used directly in the next step. ^1^H NMR (CDCl_3_) *δ* 7.40 (s, 2H), 3.19 (q, *J* = 7.3 Hz, 4H), 1.39 (t, *J* = 7.3 Hz, 6H). Found: [M + H] = 244.5.

Borane dimethyl sulfide complex (3.30 mL, 34.8 mmol) and trimethyl borate (3.90 mL, 34.8 mmol) were added to a solution of 2,6-bis(ethylthio)isonicotinic acid (2.82 g, 11.6 mmol) in THF (100 mL, dist. Na) at 0 °C. The solution was then warmed to 20 °C and was stirred for 18 h at 20 °C. The reaction mixture was cooled to 0 °C, MeOH (20 mL) was added to quench the reaction and the solvent was evaporated in the fume hood. The residue was partitioned between EtOAc and water, extracted with EtOAc (3x), dried with MgSO_4_, filtered and the solvent was evaporated. Column chromatography (4:1 hexanes:EtOAc) gave (2,6-bis(ethylthio)pyridin-4-yl)methanol (2.50 g, 94%) as a white solid. ^1^H NMR (CDCl_3_) *δ* 6.87 (s, 2H), 4.61 (s, 2H), 2.58 (s, 6H). Found: [M + H] = 202.5.

To a solution of (2,6-bis(ethylthio)pyridin-4-yl)methanol (2.48 g, 10.8 mmol) in anhydrous DCM (42 mL) was added triethylamine (2.20 mL, 16.2 mmol) dropwise, followed by methanesulfonyl chloride (1.10 mL, 14.2 mmol). The mixture was stirred at 0 °C for 10 min, then allowed to stir at 20 °C for 1 h. The mixture was quenched with saturated sodium bicarbonate solution. The aqueous mixture was extracted with dichloromethane (2x). The combined extract was washed with brine, dried with MgSO_4_ and concentrated to afford the crude product as a yellow oil. The crude intermediate was diluted in acetone (84 mL). Lithium chloride (1.85 g, 43.64 mmol) was added. The suspension was stirred at room temperature overnight. The mixture was concentrated in *vacuo* and adsorbed onto silica. Flash chromatography using a mixture of 98:2 hexane/ethyl acetate gave 4-(chloromethyl)-2,6-bis(ethylthio)pyridine (2.26 g, 84%) as a light yellow oil. ^1^H NMR (CDCl_3_) *δ* 6.87 (s, 2H), 4.61 (s, 2H), 2.58 (s, 6H). Found: [M + H] = 202.5.

A mixture of (6-bromo-2-methoxyquinolin-3-yl)boronic acid (2.56 g, 9.10 mmol), 4-(chloromethyl)-2,6-bis(ethylthio)pyridine (2.25 g, 9.10 mmol) and Cs_2_CO_3_ (5.93 g, 18.19 mmol) in toluene-DMF (60 mL, 2:1) was degassed under N_2_, then added Pd(PPh_3_)_4_ (0.52 g, 0.45 mmol), and heated at 90 °C for 1.5 h. Reaction mixture was cooled to 20 °C, filtered through a plug of celite, added water and extracted with EtOAc (x4). Organic layer washed with brine, dried with Na_2_SO_4_, filtered and the solvent was evaporated to give a yellow residue. Purification by flash column chromatography with silica using hexane:EtOAc (9:1) gave 3-((2,6-bis(ethylthio)pyridin-4-yl)methyl)-6-bromo-2-methoxyquinoline (**AB-12**) (1.80 g, 47%) as a white solid. ^1^H NMR (CDCl_3_) *δ* 7.80 (d, *J* = 2.1 Hz, 1H), 7.70 (d, *J* = 8.9 Hz, 1H), 7.65 (dd, *J* = 8.9, 2.2 Hz), 7.57 (s, 1H), 6.72 (s, 2H), 4.05 (s, 3H), 3.87 (s, 2H), 2.57 (s, 6H). Found: [M + H] = 421.8.

##### 4.1.1.13 6-Bromo-3-((2-isopropoxy-5-methoxypyridin-4-yl)methyl)-2-methoxyquinoline (**AB-13**)

Sodium metal (2.04 g, 85.0 mmol) was added to a solution of isopropanol (150 mL) and the mixture stirred at reflux for 3 h. The solution was then cooled to room temperature and 5-bromo-2-fluoropyridine (10.0 g, 56.67 mmol) was added, and the reaction heated at 80 °C for 0.5 h. The solvent was then removed and the residue partitioned between EtOAc and water, and the organic extract was dried and evaporated to afford 5-bromo-2-isopropoxypyridine (10.9 g, 89%). ^1^H NMR (CDCl_3_) *δ* 8.16 (d, *J* = 2.5 Hz, 1H), 7.60 (dd, *J* = 8.8, 2.6 Hz, 1H), 6.58 (d, *J* = 8.8 Hz, 1H), 5.23 (sep, *J* = 6.2 Hz, 1H), 1.34 (s, 3H), 1.32 (s, 3H). Found: [M + H] = 215.5.

To a solution of 5-bromo-2-isopropoxypyridine (9.85 g, 45.6 mmol) in THF (200 mL, dist. Na) at −78 °C was added *n*-BuLi (2.0 M in cyclohexane, 36.48 mL, 72.96 mmol) dropwise over 10 min. The reaction was stirred for 20 min and then trimethyl borate (13.24 mL, 72.96 mmol) was added dropwise over 5 min. The resulting mixture was stirred for 2 h at −78 °C, then peracetic acid solution (32 wt% in dilute acetic acid, 25.69 mL, 72.96 mmol) was added. After 10 min at −78 °C, the reaction was warmed to 0 °C and stirred for 1 h. The reaction was then quenched with aqueous sodium bisulfite and stirred for 15 min at 0 °C. The solvent was then concentrated, sodium bicarbonate added and the aqueous layer extracted with EtOAc. The combined organic extracts were dried and evaporated. Column chromatography with 4:1 x4:EtOAc afforded 6-isopropoxypyridin-3-ol (5.40 g, 65%). ^1^H NMR (CDCl_3_) *δ* 7.76 (dd, *J* = 3.1, 0.4 Hz, 1H), 7.17 (dd, *J* = 8.9, 3.1 Hz, 1H), 6.61 (d, *J* = 8.9 Hz, 1H), 6.16 (br s, 1H), 5.09 (sep, *J* = 6.1 Hz, 1H), 1.34 (s, 3H), 1.32 (s, 3H).

To a solution of 6-isopropoxypyridin-3-ol (6.00 g, 32.97 mmol) in DMF (80 mL, anhydrous) at 0 °C was added sodium hydride (60% in mineral dispersion, 1.57 g, 39.54 mmol) in portions. The mixture was warmed to 20 °C and stirred for 1 h. Chloromethyl ethyl ether (3.50 mL, 37.71 mmol) was then added, and the resultant mixture stirred at 20 °C for a further 2 h. The reaction was diluted with brine (100 mL) and extracted with EtOAc (x3) times. The organic layer was washed with brine (x3), dried and evaporated. Column chromatography with 19:1 x4:EtOAc afforded 5-(ethoxymethoxy)-2-isopropoxypyridine (6.50 g, 82%). ^1^H NMR (CDCl_3_) *δ* 7.94 (d, *J* = 3.0 Hz, 1H), 7.33–7.30 (m, 1H), 6.61 (d, *J* = 8.9 Hz, 1H), 5.20 (sep, *J* = 6.2 Hz, 1H), 5.14 (s, 2H), 3.73 (q, *J* = 7.0 Hz, 2H), 1.33 (s, 3H), 1.32 (s, 3H), 1.23 (t, *J* = 7.1 Hz, 3H). Found: [M + H] = 211.5.

To a solution of 5-(ethoxymethoxy)-2-isopropoxypyridine (6.50 g, 26.97 mmol) and diisopropylamine (0.19 mL, 1.35 mmol) in THF (80 mL, dist. Na) at −40 °C under nitrogen was added *n*-BuLi (2.0 M in cyclohexane, 16.18 mL, 32.36 mmol) dropwise. The resultant solution was stirred at −40 °C for 5 min, and then warmed to 0 °C and stirred at this temperature for a further 3 h. The solution was then again cooled to −40 °C, and *n*-formylpiperidine (4.49 mL, 40.46 mmol) was added dropwise, and the mixture stirred at 20 °C for another 1 h. Acetic acid (12 mL) was added and the solvent was removed in *vacuo*. The resultant mixture was partitioned between EtOAc and water, and the organic fraction dried and evaporated. Column chromatography with 19:1 x4:EtOAc afforded 5-(ethoxymethoxy)-2-isopropoxyisonicotinaldehyde (5.51 g, 76%). ^1^H NMR (CDCl_3_) *δ* 10.41 (s, 1H), 8.25 (s, 1H), 7.01 (d, *J* = 0.5 Hz, 1H), 5.29 (s, 2H), 5.23–5.16 (m, 1H), 3.79 (q, *J* = 7.1 Hz, 2H), 1.34 (s, 3H), 1.32 (s, 3H), 1.26 (t, *J* = 7.1 Hz, 3H).

A solution of 5-(ethoxymethoxy)-2-isopropoxyisonicotinaldehyde (5.51 g, 20.48 mmol) and 3 M HCl (65 mL) in THF (45 mL, dist. Na) was heated at 40 °C for 1.5 h. The solution was then cooled, diluted with water, and the pH adjusted to 7 using sodium hydrogen carbonate. The aqueous layer was then extracted with EtOAc (x3), and the organic layer dried and evaporated to afford 5-hydroxy-2-isopropoxyisonicotinaldehyde (3.71 g, 100%). ^1^H NMR (CDCl_3_) *δ* 9.96 (s, 1H), 8.06 (s, 1H), 6.87 (s, 1H), 5.21 (sep, *J* = 6.2 Hz, 1H), 1.34 (s, 3H), 1.32 (s, 3H).

A mixture of 5-hydroxy-2-isopropoxyisonicotinaldehyde (3.80 g, 20.99 mmol) and potassium carbonate (4.35 g, 31.49 mmol) in DMF (100 mL, anhydrous) was heated at 50 °C for 10 min. Methyl iodide (1.57 mL, 25.19 mmol) was then added and the mixture stirred at this temperature for 2 h. The resultant solution was diluted with EtOAc and washed with brine (x3). The organic layer was dried and evaporated. Column chromatography with 19:1 x4:EtOAc afforded 2-isopropoxy-5-methoxyisonicotinaldehyde (2.60 g, 63%). ^1^H NMR (CDCl_3_) *δ* 10.42 (s, 1H), 7.99 (s, 1H), 7.02 (d, *J* = 0.5 Hz, 1H), 5.17 (sep, *J* = 6.2 Hz, 1H), 1.34 (s, 3H), 1.32 (s, 3H).

A mixture of 2-isopropoxy-5-methoxyisonicotinaldehyde (2.60 g, 13.32 mmol) and sodium borohydride (1.01 g, 26.65 mmol) in methanol (50 mL, anhydrous) was stirred at 20 °C for 1 h. The solvent was then removed and the residue partitioned between EtOAc and water. The organic layer was dried and evaporated to afford (2-isopropoxy-5-methoxypyridin-4-yl)methanol (2.59 g, 99%). ^1^H NMR (CDCl_3_) *δ* 7.68 (s, 1H), 6.71 (s, 1H), 5.17 (sep, *J* = 6.2 Hz, 1H), 4.64 (d, *J* = 5.7 Hz, 2H), 3.85 (s, 3H), 2.40 (t, *J* = 6.1 Hz, 1H), 1.33 (s, 3H), 1.31 (s, 3H).

To a solution of (2-isopropoxy-5-methoxypyridin-4-yl)methanol (2.59 g, 13.14 mmol) and triethylamine (2.74 mL, 19.71 mmol) in DCM (40 mL, anhydrous) at 20 °C was added methanesulfonyl chloride (1.23 mL, 15.77 mmol) dropwise. After 15 min, the reaction was diluted with DCM (40 mL) and the organic layer washed with sat. sodium hydrogen carbonate, dried and evaporated. The residue was redissolved in acetone (80 mL, anhydrous), Lithium bromide (10 g, excess) added, and the mixture heated at reflux for 30 min. The solution was then cooled and the solvent evaporated, and the residue partitioned between EtOAc and water. The aqueous layer was extracted with EtOAC (x2) and the organic layer was dried and evaporated to afford 4-(bromomethyl)-2-isopropoxy-5-methoxypyridine (3.42 g, 100%). ^1^H NMR (CDCl_3_) *δ* 7.73 (s, 1H), 6.69 (s, 1H), 5.17 (sep, *J* = 6.2 Hz, 1H), 4.38 (s, 2H), 3.90 (s, 3H),1.33 (s, 3H), 1.31 (s, 3H).

A mixture of (6-bromo-2-methoxyquinolin-3-yl)boronic acid (3.92 g, 13.92 mmmol), 4-(bromomethyl)-2-isopropoxy-5-methoxypyridine (3.45 g, 13.26 mmol) and cesium carbonate (8.64 g, 26.52 mmol) in toluene (50 mL, anhydrous) and DMF (25 mL, anhydrous) was purged with nitrogen. Pd(PPh_3_)_4_ (0.61 g, 0.53 mmol) was then added, the mixture purged with nitrogen then heated to 80 °C under nitrogen for 4 h. The reaction was partitioned between EtOAc and water and the organic fraction was dried and evaporated. Column chromatography (19:1 x4:EtOAc) gave 6-bromo-3-((2-isopropoxy-5-methoxypyridin-4-yl)methyl)-2-methoxyquinoline (**AB-13**) (2.85 g, 49%). ^1^H NMR (CDCl_3_) *δ* 7.77 (d, *J* = 2.2 Hz, 1H), 7.72 (s, 1H), 7.70 (d, *J* = 8.9 Hz, 1H), 7.62 (dd, *J* = 8.9, 2.2 Hz, 1H), 7.54 (s, 1H), 6.38 (s, 1H), 5.17 (sep, *J* = 6.2 Hz, 1H), 4.07 (s, 3H), 3.96 (s, 2H), 3.83 (s, 3H), 1.31 (s, 3H), 1.29 (s, 3H).

##### 4.1.1.14 6-Bromo-3-((2-cyclobutoxy-6-methoxypyridin-4-yl)methyl)-2-methoxyquinoline (**AB-14**)

A solution of methyl 2-hydroxy-6-methoxyisonicotinate (3.00 g, 16.4 mmol) in DMF (50 mL, anhydrous) was treated with K_2_CO_3_ (4.52 g, 32.7 mmol) and bromocyclobutane (2.00 mL, 25.0 mmol). The mixture was stirred at 20 °C for 48 h, partitioned between EtOAc and water and the aqueous layer was extracted with EtOAc. The combined organic fractions were washed with water, dried and evaporated. Column chromatography (DCM) gave methyl 2-cyclobutoxy-6-methoxyisonicotinate (2.215 g, 57%) as a colourless oil. ^1^H NMR (CDCl_3_) *δ* 6.84 (d, *J* = 1.0 Hz, 1H), 6.79 (d, *J* = 1.0 Hz, 1H), 5.08 (pd, *J* = 7.4, 0.8 Hz, 1H), 3.91 (s, 3H), 3.90 (s, 3H), 2.42–2.52 (m, 2H), 2.12–2.24 (m, 2H), 1.80–1.90 (m, 1H), 1.62–1.75 (m, 1H). Found: [M + H] = 238.2.

A solution of LiOH (0.71 g, 29.6 mmol) in water (20 mL) was added to a solution of methyl 2-cyclobutoxy-6-methoxyisonicotinate (2.205 g, 9.29 mmol) in MeOH (20 mL) and THF (20 mL); the solution was stirred at 20 °C for 18 h and then evaporated. The residue was dissolved in water (80 mL) and acidified to pH 3 with 2 M HCl. The resulting precipitate was filtered and dried to give 2-cyclobutoxy-6-methoxyisonicotinic acid (2.02 g, 97%) as a white solid. M.p. 170–171 °C. ^1^H NMR (DMSO‑*d*_6_) *δ* 13.56 (bs, 1H), 6.74 (d, *J* = 1.0 Hz, 1H), 6.67 (d, *J* = 1.0 Hz, 1H), 5.07 (pd, *J* = 7.1, 0.7 Hz, 1H), 3.85 (s, 3H), 2.37–2.46 (m, 2H), 2.14–2.22 (m, 2H), 1.74–1.83 (m, 1H), 1.59–1.72 (m, 1H). Found: [M + H] = 224.2.

Trimethyl borate (1.03 mL, 9.0 mmol) and borane dimethyl sulphide complex were added sequentially to a solution of 2-cyclobutoxy-6-methoxyisonicotinic acid (1.01 g, 4.52 mmol) in anhydrous THF (20 mL) at 0 °C. The solution was stirred at 20 °C for 18 h and then quenched with MeOH. Removal of the solvent gave an oil, chromatography (3:1 hexanes:EtOAc) of the crude product gave (2-cyclobutoxy-6-methoxypyridin-4-yl)methanol (0.91 g, 96%) as a colourless oil. ^1^H NMR (CDCl_3_) *δ* 6.29 (d, *J* = 0.9 Hz, 1H), 6.23 (d, *J* = 0.9 Hz, 1H), 5.06 (pd, *J* = 7.2, 0.9 Hz, 1H), 4.62 (d, *J* = 6.2 Hz, 2H), 3.88 (s, 3H), 2.41–2.49 (m, 2H), 2.11–2.22 (m, 2H), 1.79–1.89 (m, 1H), 1.63–1.75 (m, 2H). Found: [M + H] = 210.2

A solution of (2-cyclobutoxy-6-methoxypyridin-4-yl)methanol 0.842 g, 4.04 mmol) in DCM (25 mL) at 0 °C was treated sequentially with Et_3_N (2.25 mL, 16.1 mmol) and mesyl chloride (0.47 mL, 6.1 mmol), the solution was stirred for 1 h at 0 °C and then partitioned with water, the organic fraction was dried and evaporated. The residue was dissolved in acetone (50 mL), LiBr (3.50 g, 40.3 mmol) was added and the mixture was refluxed for 0.5 h and then evaporated. The residue was partitioned between DCM and water and the organic fraction was dried and evaporated. Column chromatography (DCM) gave 4-(bromomethyl)-2-cyclobutoxy-6-methoxypyridine (0.994 g, 90%) as a colourless oil. ^1^H NMR (CDCl_3_) *δ* 6.30 (d, *J* = 1.0 Hz, 1H), 6.25 (d, *J* = 1.0 Hz, 1H), 5.07 (pd, *J* = 7.1, 0.9 Hz, 1H), 4.27 (s, 2H), 3.88 (s, 3H), 2.41–2.49 (m, 2H), 2.11–2.22 (m, 2H), 1.79–1.89 (m, 1H), 1.61–1.75 (m, 1H). Found: [M + H] = 272, 274.

A mixture of (6-bromo-2-methoxyquinolin-3-yl)boronic acid (1.010 g, 3.58 mmol), 4-(bromomethyl)-2-cyclobutoxy-6-methoxypyridine (0.975 g, 3.58 mmol) and Cs_2_CO_3_ (2.33 g, 7.2 mmol) in toluene/DMF (2:1, 50 mL) was purged with nitrogen. Pd(PPh_3_)_4_ (0.083 g, 0.072 mmol) was added and the mixture was heated to 80 °C for 3 h under an atmosphere of nitrogen. The mixture was partitioned between EtOAc and water, the organic fraction was dried and evaporated. Column chromatography using a gradient of 3:1 hexanes:DCM to DCM gave 6-bromo-3-((2-cyclobutoxy-6-methoxypyridin-4-yl)methyl)-2-methoxyquinoline (**AB-14)** (1.195 g, 78%) as a white solid. M.p. 101–102 °C. ^1^H NMR (CDCl_3_) *δ* 7.77 (d, *J* = 2.2 Hz, 1H), 7.69 (d, *J* = 8.9 Hz, 1H), 7.62 (dd, *J* = 8.9, 2.2 Hz, 1H), 7.52 (s, 1H), 6.16 (d, *J* = 0.3 Hz, 1H), 6.10 (d, *J* = 0.3 Hz, 1H), 5.06 (pd, *J* = 7.9, 0.9 Hz, 1H), 4.06 (s, 3H), 3.90 (s, 2H), 3.87 (s, 3H), 2.38–2.48 (m, 2H), 2.09–2.21 (m, 2H), 1.77–1.87 (m, 1H), 1.62–1.72 (m, 1H). Found: [M + H] = 429.1, 431.1.

##### 4.1.1.15 6-Bromo-3-((2-ethoxy-6-isopropoxypyridin-4-yl)methyl)-2-methoxyquinoline (**AB-15**)

Potassium carbonate (8.65 g, 125 mmol) and 2-iodopropane (12.8 mL, 128 mmol) were added to a solution of ethyl 2-ethoxy-6-hydroxyisonicotinate (10.82 g, 51.2 mmol) in anhydrous DMF (125 mL) and the mixture was stirred at 20 °C for 48 h. 2-Iodopropane (12.8 mL, 128 mmol) and potassium carbonate (8.65 g, 125 mmol) were added and the mixture was stirred for a further 24 h and then partitioned between DCM and water. The organic fraction was dried and evaporated, chromatography using DCM as an eluent gave ethyl 2-ethoxy-6-isopropoxyisonicotinate (11.557 g, 89%) as a colourless oil. ^1^H NMR (CDCl_3_) *δ* 6.81 (s, 2H), 5.23 (sp, *J* = 6.2 Hz, 1H), 4.36 (t, *J* = 7.2 Hz, 2H), 4.32 (t, *J* = 7.1 Hz, 2H), 1.33–1.42 (m, 12H). Found: [M + H] = 254.2.

A solution of LiOH (3.25 g, 136 mmol) in water (60 mL) was added to a solution of ethyl 2-ethoxy-6-isopropoxyisonicotinate (11.424 g, 45.1 mmol) in THF (60 mL) and MeOH (60 mL), the solution was stirred at 20 °C for 60 h then evaporated. The residue was dissolved in water (200 mL) and the solution was adjusted to pH 6 with 2 M HCl. The oily solid was extracted with EtOAc, the organic fractions were washed with water, dried and evaporated to give 2-ethoxy-6-isopropoxyisonicotinic acid (10.034 g, 99%) as a white solid. M.p. > 300 °C. ^1^H NMR (DMSO‑*d*_6_) *δ* 13.48 (bs, 1H), 6.66 (d, *J* = 1.0 Hz, 1H), 6.64 (d, *J* = 1.0 Hz, 1H), 5.17 (sp, *J* = 6.2 Hz, 1H), 4.50 (q, *J* = 7.0 Hz, 2H), 1.28–1.34 (m, 9H). No diagnostic peak in the mass spectrum.

Trimethylborate (3.03 mL, 26.7 mmol) and then borane-dimethylsulfide (2.53 mL, 26.7 mmol) were added to a solution of 2-ethoxy-6-isopropoxyisonicotinic acid (3.00 g, 13.3 mmol) in THF (50 mL, dist. Na) at 0 °C and the mixture was stirred at 20 °C for 18 hr. The solution was cooled to 0 °C and methanol was cautiously added to quench the reaction. Removal of the solvent gave a solid, this was partitioned between EtOAc and water, and the organic fraction was dried and evaporated. Column chromatography (3:1 hexanes:EtOAc) gave (2-ethoxy-6-isopropoxypyridin-4-yl)methanol (2.72 g, 97%) as a colourless oil. ^1^H NMR (CDCl_3_) *δ* 6.24 (s, 1H), 6.23 (s, 1H), 5.21 (sp, *J* = 6.2 Hz, 1H), 4.60 (d, *J* = 6.2 Hz, 2H), 4.30 (q, *J* = 7.0 Hz, 2H), 1.70 (t, *J* = 6.2 Hz, 1H), 1.38 (t, *J* = 7.0 Hz, 3H), 1.34 (d, *J* = 6.2 Hz, 6H). Found: [M + H] = 212.2.

A solution of (2-ethoxy-6-isopropoxypyridin-4-yl)methanol (2.60 g, 12.3 mmol) in DCM (50 mL, anhydrous) at 0 °C was treated sequentially with triethylamine (3.43 mL, 24.6 mmol) then mesyl chloride (1.43 mL, 18.5 mmol), the mixture was stirred at 0 °C for 1 h then partitioned between DCM and water. The organic fraction was dried and evaporated and the residue was dissolved in acetone (100 mL), LiBr (10.7 g, 123 mmol) was added and the mixture was refluxed for 1 h and then evaporated. The residue was partitioned between DCM and water; the organic fraction was dried and evaporated. Column chromatography (DCM) gave 4-(bromomethyl)-2-ethoxy-6-isopropoxypyridine (3.04 g, 100%) as a colourless oil. ^1^H NMR (CDCl_3_) *δ* 6.26 (s, 1H), 6.25 (s, 1H), 5.20 (sp, *J* = 6.2 Hz, 1H), 4.26–4.33 (m, 4H), 1.38 (t, *J* = 7.0 Hz, 3H), 1.34 (d, *J* = 6.2 Hz, 6H). Found: [M + H] = 274.1.

A mixture of (6-bromo-2-methoxyquinolin-3-yl)boronic acid (3.27 g, 11.6 mmol), (4-(bromomethyl)-2-ethoxy-6-isopropoxypyridine (3.18 g, 11.6 mmol) and Cs_2_CO_3_ (7.56 g, 23.2 mmol) in toluene (66 mL) and DMF (33 mL) was purged with nitrogen. Pd(PPh_3_)_4_ (0.27 g, 0.23 mmol) was added, the mixture was purged with nitrogen then heated to 80 °C under nitrogen for 3 h. The reaction was partitioned between EtOAc and water and the organic fraction was dried and evaporated. Column chromatography with 3:1 hexanes:DCM eluted impurities, then elution with DCM gave 6-bromo-3-((2-ethoxy-6-isopropoxypyridin-4-yl)methyl)-2-methoxyquinoline (**AB-15**) (3.42 g, 68%) as white solid. M.p. 78–80 °C. ^1^H NMR (CDCl_3_) *δ* 7.77 (d, *J* = 2.1 Hz, 1H), 7.68 (d, *J* = 8.8 Hz, 1H), 7.62 (dd, *J* = 8.8, 2.2 Hz, 1H), 7.58 (s, 1H), 6.11 (s, 1H), 6.10 (s, 1H), 5.20 (sp, *J* = 6.2 Hz, 1H), 4.28 (q, *J* = 7.1 Hz, 2H), 4.06 (s, 3H), 3.89 (s, 2H), 1.36 (t, *J* = 7.1 Hz, 3H), 1.32 (d, *J* = 6.2 Hz, 6H). Found: [M + H] = 431.1.

##### 4.1.1.16 6-Bromo-2-methoxy-3-((2,3,5-trimethoxypyridin-4-yl)methyl)quinolone (**AB-16**)

To a solution of 2,6-dimethoxypyridine (10 g, 71.84 mmol) and *N*,*N*-diisopropylamine (0.50 mL, 3.59 mmol) in THF (200 mL, dist. Na) at −40 °C under nitrogen was added *n*-BuLi (43.10 mL, 86.21 mmol) dropwise. The resultant solution was stirred at −40 °C for 5 min, and then warmed to 0 °C and stirred at this temperature for a further 3 h. The solution was then again cooled to −40 °C, and triisopropylborate (24.87 mL, 107.76 mmol) was added dropwise, and the mixture stirred at 20 °C for another 1 h. Water (50 mL) was added and the solvent was removed in *vacuo*. To the residue was added 1 M NaOH (100 mL) and the aqueous layer washed with EtOAc (2 × 100 mL). The aqueous layer was then acidified to pH 3 and a solid precipitated. This solid was filtered and dried to afford 2,6-dimethoxypyridin-3-yl)boronic acid (8.10 g, 61%). ^1^H NMR (DMSO‑*d*_6_) *δ* 7.87 (1H, d, *J* = 7.9 Hz), 6.36 (1H, d, *J* = 7.9 Hz), 3.90 (3H, s), 3.87 (3H, s).

To a solution of 2,6-dimethoxypyridin-3-yl)boronic acid (8.00 g, 43.49 mmol) in THF (150 mL, dist. Na) at 0 °C was added dropwise 32% peracetic acid in acetic acid (21.53 mL, 86.98 mmol) over 10 min. The resultant solution was stirred at 20 °C. for 2 h. A 10% solution of sodium sulfite (75 mL) was then added and the mixture stirred at 20 °C for 0.5 h. The solvent was evaporated and the residue partitioned between EtOAc and water. The aqueous layer was extracted twice and the organic layer dried and evaporated. Column chromatography with 9:1 hexanes/EtOAc afforded 2,6-dimethoxypyridin-3-ol (6.05 g, 90%). ^1^H NMR (CDCl_3_) *δ* 7.12 (1H, d, *J* = 8.3 Hz), 6.21 (1H, d, *J* = 8.2 Hz), 4.90 (1H, s), 7.00 (3H, s), 3.86 (3H, s). Found: [M + H] = 156.7.

To a solution of 2,6-dimethoxypyridin-3-ol (6.45 g, 40.97 mmol) in DMF (70 mL, anhydrous) at 0 °C was added 60% sodium hydride in mineral oil (41.97 g, 9.16 mmol) in portions. The mixture was warmed to 20 °C and stirred for 1 h. 1-Chloro-2-methoxyethane (4.37 mL, 47.11 mmol) was then added, and the resultant mixture stirred at 20 °C for a further 2 h. The reaction was diluted with brine (100 mL) and extracted with EtOAc three times. The organic layer was washed with brine three times, dried and evaporated. Column chromatography with 19:1 hexanes/EtOAc afforded 3-(ethoxymethoxy)-2,6-dimethoxypyridine (8.14 g, 93%). ^1^H NMR (CDCl_3_) *δ* 7.41–7.33 (1H, m), 6.26–6.17 (1H, m), 5.15 (2H, d, *J* = 1.9 Hz), 3.98 (3H, d, *J* = 1.8 Hz), 3.87 (3H, d, *J* = 2.0 Hz), 3.77 (2H, dq, *J* = 1.8, 7.1 Hz), 1.22 (3H, dt, *J* = 2.9, 7.0 Hz).

To a solution of 3-(ethoxymethoxy)-2,6-dimethoxypyridine (4.00 g, 18.78 mmol) and *N*,*N*-diisopropylamine (0.13 mL, 0.94 mmol) in THF (60 mL, dist. Na) at −40 °C under nitrogen was added *n*-BuLi (2.0 M in cyclohexane, 14.09 mL, 28.17 mmol) dropwise. The resultant solution was stirred at −40 °C for 5 min, and then warmed to 0 °C and stirred at this temperature for a further 3 h. The solution was then again cooled to −40 °C, and 1-formylpiperidine (3.75 mL, 33.80 mmol) was added dropwise, and the mixture stirred at 20 °C for another 1 h. Acetic acid (7.5 mL) was added and the solvent was removed in *vacuo*. The resultant mixture was partitioned between EtOAc and water, and the organic fraction dried and evaporated. Column chromatography with 19:1 hexanes/EtOAc afforded 3-(ethoxymethoxy)-2,6-dimethoxyisonicotinaldehyde (2.30 g, 51%). ^1^H NMR (CDCl_3_) *δ* 10.39 (1H, s), 6.61 (1H, s), 6.19 (2H, s), 4.02 (3H, s), 3.88 (3H, s), 3.78 (2H, q, *J* = 10.1 Hz), 1.21 (3H, t, *J* = 7.1 Hz).

A solution of 3-(ethoxymethoxy)-2,6-dimethoxyisonicotinaldehyde (2.30 g, 9.54 mmol) and 3 M hydrochloric acid (60 mL) in THF (30 mL, dist. Na) was heated at 40 °C for 1.5 h. The solution was then cooled, diluted with water, and the pH adjusted to 7 using NaHCO_3_. The aqueous layer was then extracted with EtOAc three times, and the organic layer dried and evaporated. Column chromatography with 19:1 hexanes/EtOAc afforded 3-hydroxy-2,6-dimethoxyisonicotinaldehyde (1.36 g, 78%). ^1^H NMR (CDCl_3_) *δ* 9.96 (1H, s), 9.61 (1H, s), 6.46 (1H, s), 4.06 (3H, s), 3.91 (3H, s).

A mixture of 3-hydroxy-2,6-dimethoxyisonicotinaldehyde (1.35 g, 7.38 mmol) and potassium carbonate (1.53 g, 11.07 mmol) in DMF (40 mL, anhydrous) was heated at 50 °C for 10 min. Methyl iodide (0.56 mL, 8.86 mmol) was then added and the mixture stirred at this temperature for 2 h. The resultant solution was diluted with EtOAc and washed with brine three times. The organic layer was dried and evaporated to afford the product 2,3,6-trimethoxyisonicotinaldehyde (1.40 g, 96%). ^1^H NMR (CDCl_3_) *δ* 10.40 (1H, s), 6.58 (1H, s), 4.04 (3H, s), 3.93 (3H, s), 3.91 (3H, s).

A mixture of 2,3,6-trimethoxyisonicotinaldehyde (1.40 g, 7.11 mmol) and sodium borohydride (0.54 g, 14.21 mmol) in MeOH (30 mL, anhydrous) was stirred at 20 °C for 1 h. The solvent was then removed and the residue partitioned between EtOAc and water. The organic layer was dried and evaporated to afford the product (2,3,6-trimethoxypyridin-4-yl)methanol (1.35 g, 95%). ^1^H NMR (CDCl_3_) *δ* 6.30 (1H, s), 4.68 (2H, d, *J* = 5.6 Hz), 3.99 (3H, s), 3.88 (3H, s), 3.79 (3H, s), 2.21 (1H, t, *J* = 5.9 Hz).

To a solution of (2,3,6-trimethoxypyridin-4-yl)methanol (1.35 g, 6.78 mmol) and triethylamine (1.42 mL, 10.78 mmol) in DCM (20 mL, anhydrous) at 20 °C was added mesyl chloride (0.63 mL, 8.14 mmol) dropwise. After 15 min, the reaction was diluted with DCM (20 mL) and the organic layer washed with sat. aq. NaHCO_3_, dried and evaporated. The residue was dissolved in acetone (40 mL, anhydrous), lithium bromide (excess) added, and the mixture heated at reflux for 30 min. The solution was then cooled and the solvent evaporated, and the residue partitioned between EtOAc and water. The aqueous layer was extracted twice with EtOAc and the organic layer was dried and evaporated to give the product 4-(bromomethyl)-2,3,6-trimethoxypyridine (1.69 g, 95%). ^1^H NMR (CDCl_3_) *δ* 6.27 (1H, s), 4.40 (2H, s), 3.98 (3H, s), 3.87 (3H, s), 3.87 (3H, s). Found: [M + H] = 262.5

A mixture of (6-bromo-2-methoxyquinolin-3-yl)boronic acid (1.89 g, 6.69 mmol), 4-(bromomethyl)-2,3,6-trimethoxypyridine (1.67 g, 6.37 mmol) and cesium carbonate (4.15 g, 12.74 mmol) in toluene (40 mL, anhydrous) and DMF (20 mL, anhydrous) was purged with nitrogen. Pd(PPh_3_)_4_ (0.29 g, 0.26 mmol) was then added, the mixture purged with nitrogen then heated to 80 °C under nitrogen for 4 h. The reaction was partitioned between EtOAc and water and the organic fraction was dried and evaporated. Column chromatography (19:1 hexanes/EtOAc) gave the product 6-bromo-2-methoxy-3-((2,3,5-trimethoxypyridin-4-yl)methyl)quinoline (**AB-16**) (1.44 g, 54%). ^1^H NMR (CDCl_3_) *δ* 7.76 (1H, d, *J* = 2.2 Hz), 7.68 (1H, d, *J* = 8.9 Hz), 7.61 (1H, dd, *J* = 2.2, 8.8 Hz), 7.54 (1H, s), 6.04 (1H, s), 4.07 (3H, s), 4.00 (3H, s), 4.39 (2H, s), 3.85 (3H, s), 3.72 (3H, s). Found: [M + H] = 420.0

##### 4.1.1.17 4-((6-Bromo-2-methoxyquinolin-3-yl)methyl)-6-methoxy-N,*N*-dimethylpyridin-2-amine (**AB-17**)

To a solution of (2-(dimethylamino)-6-methoxypyridin-4-yl)methanol (3.30 g, 18.1 mmol) in anhydrous DCM (54 mL) was added at 2 °C under nitrogen triethylamine (3.8 mL, 27.2 mmol) dropwise, followed by mesyl chloride (1.7 mL, 21.7 mmol). The mixture was stirred from 2 °C for 10 min, then at 20 °C. for 2 h. The mixture was quenched with sat. aq. NaHCO_3_ solution. The aqueous mixture was extracted with DCM (2x) and the combined extract was washed with brine, dried (MgSO_4_) and concentrated to afford the crude product as a beige solid. The crude intermediate was dissolved in acetone (60 mL). Lithium bromide (1.53 g, 36.2 mmol) was added and the suspension was refluxed for 2 h. Flash chromatography using a mixture of 3–5% Et_2_O in hexanes as eluent gave 4-(bromomethyl)-6-methoxy-*N*,*N*-dimethylpyridin-2-amine (3.78 g, 85%) as a mobile yellow oil. ^1^H NMR (CDCl_3_) *δ* 6.02 (1H, d, *J* = 0.8 Hz), 6.00 (1H, d, *J* = 0.4 Hz), 4.27 (2H, s), 3.88 (3H, s), 3.06 (6H, s).

A mixture of (6-bromo-2-methoxyquinolin-3-yl)boronic acid (4.34 g, 15.4 mmol), 4-(bromomethyl)-6-methoxy-*N*,*N*-dimethylpyridin-2-amine (3.77 g, 115.4 mmol) and cesium carbonate (10.03 g, 30.8 mmol) in a mixture of toluene (40 mL) and DMF (20 mL) was purged with nitrogen. Pd(PPh_3_)_4_ (0.890 g, 0.774 mmol) was added and the mixture was purged again with nitrogen and heated at 85 °C under nitrogen for 3.5 h. The mixture was partitioned between water and EtOAc and the mixture was extracted with EtOAc (2x). The extract was washed with water (2x), brine, dried (MgSO_4_) and concentrated to afford the crude product as a brown solid which was chromatographed using 3–10% Et_2_O in hexanes as eluent to yield the product 4-((6-bromo-2-methoxyquinolin-3-yl)methyl)-6-methoxy-*N*,*N*-dimethylpyridin-2-amine (**AB-17**) (3.69 g, 60%) as a light yellow solid. ^1^H NMR (CDCl_3_) *δ* 7.76 (1H, d, *J* = 2 Hz), 7.68 (1H, d, *J* = 8.8 Hz), 7.61 (1H, dd, *J* = 2, 8.8 Hz), 7.55 (1H, s), 5.92 (1H, s), 5.86 (1H, s), 4.07 (3H, s), 3.87 (5H, s), 3.04 (6H, s). Found: [M + H] = 402.0

##### 4.1.1.18 4-((6-Bromo-2-methoxyquinolin-3-yl)methyl)-6-ethoxy-*N*,*N*-dimethylpyridin-2-amine (**AB-18**)

To a glass tube was charged ethyl 2-chloro-6-ethoxyisonicotinate (WO 2010/080864) (1.00 g, 4.37 mmol), diphenylphosphino-1,1′-binaphthol (0.44 g, 0.70 mmol) and cesium carbonate (1.99 g, 6.12 mmol) under continuous nitrogen flow. Anhydrous toluene (24 mL) was added. The mixture was purged with nitrogen 5 min. Palladium acetate (0.079 g, 0.35 mmol) was added, the mixture was purged again with nitrogen. Dimethylamine in THF (2 N, 2.6 mL, 5.246 mmol) was added and the mixture was sealed in the tube and heated at 80 °C overnight. The mixture was filtered through Celite, washing with EtOAc and the filtrate was concentrated in *vacuo* to yield the crude product as a dark red liquid. Flash chromatography using 2–4% Et_2_O in hexanes gave ethyl 2-(dimethylamino)-6-ethoxyisonicotinate (0.85 g, 82%) as a light yellow oil. ^1^H NMR (CDCl_3_) *δ* 6.60 (1H, d, *J* = 0.8 Hz), 6.51 (1H, d, *J* = 0.8 Hz), 4.35 (2H, q, *J* = 7.0 Hz), 4.33 (2H, q, *J* = 7.1 Hz), 3.09 (6H, s), 1.38 (3H, t, *J* = 7.2 Hz), 1.37 (3H, t, *J* = 7.2 Hz).

To a solution of ethyl 2-(dimethylamino)-6-ethoxyisonicotinate (4.40 g, 18.50 mmol) in freshly distilled THF (90 mL) was added at −78 °C under nitrogen lithium aluminium hydride (0.91 g, 24.0 mmol) in 3 batches. The mixture was stirred at −78 °C for 15 min then at 20 °C for 1 h. The mixture was quenched cautiously with water at 2 °C until gas evolution ceased. 1 M NaOH (32 mL) was added and the mixture was stirred for 1 h, then the aqueous mixture was diluted with water and extracted with EtOAc (3x). The combined organic extract was washed with brine, dried (Na_2_SO_4_) and concentrated to give the product, which was purified via flash chromatography eluting with mixtures of 6:1 then 4:1 hexanes/EtOAc to give the alcohol intermediate (3.27 g, 90%) as a light yellow oil. The material was used directly in the next step without further characterisation.

To a solution of the alcohol intermediate (3.27 g, 16.7 mmol) in anhydrous DCM (50 mL) was added at 2 °C under nitrogen triethylamine (3.5 mL, 25.0 mmol) dropwise, followed by mesyl chloride (1.6 mL, 20.0 mmol). The mixture was stirred from 2 °C for 10 min, then at 20 °C for 0.5 h. The mixture was quenched with sat. aq. NaHCO_3_ solution. The aqueous mixture was extracted with DCM (3x) and the combined extract was washed with brine, dried and concentrated to afford the crude product as a brown oil. This was diluted in acetone (60 mL), lithium bromide (1.42 g) was added. And the suspension was refluxed for 2 h. Flash chromatography using a mixture of 2–3% Et_2_O in hexanes as eluent gave 4-(bromomethyl)-6-ethoxy-*N*,*N*-dimethylpyridin-2-amine (3.84 g, 89%) as a mobile yellow oil. ^1^H NMR (CDCl_3_) *δ* 6.011 (1H, s), 5.99 (1H, s), 4.30 (2H, q, *J* = 6.8 Hz), 4.27 (2H, s), 3.05 (6H, s), 1.37 (3H, t, *J* = 7.2 Hz).

A mixture of (6-bromo-2-methoxyquinolin-3-yl)boronic acid (4.13 g, 14.7 mmol), 4-(bromomethyl)-6-ethoxy-*N*,*N*-dimethylpyridin-2-amine (3.80 g, 14.7 mmol) and cesium carbonate (9.58 g, 29.4 mmol) in a mixture of toluene (40 mL) and DMF (20 mL) was purged with nitrogen. Pd(PPh_3_)_4_ (0.68 g) was added. The mixture was purged again with nitrogen and heated at 85 °C under nitrogen for 3 h. The mixture was partitioned between water and EtOAc and the aqueous mixture was extracted with EtOAc (2x).The extract was washed with water (2x), brine, dried and concentrated to afford the crude product as a brown oil. This was chromatographed using 3–10% Et_2_O in hexanes as eluent to give the product as a yellow solid (3.83 g), which was triturated in diethyl ether to afford yield 4-((6-bromo-2-methoxyquinolin-3-yl)methyl)-6-ethoxy-*N*,*N*-dimethylpyridin-2-amine (**AB-18**) (3.51 g, 57%) as a pale yellow solid. ^1^H NMR (CDCl_3_) *δ* 7.76 (1H, d, *J* = 2.2 Hz), 7.68 (1H, d, *J* = 8.8 Hz), 7.61 (1H, dd, *J* = 2.2, 8.8 Hz), 7.55 (1H, s), 5.91 (1H, s), 5.84 (1H, s), 4.30 (2H, q, *J* = 7.1 Hz), 4.07 (3H, s), 3.87 (2H, s), 3.03 (6H, s), 1.36 (3H, t, *J* = 7.1 Hz).

##### 4.1.1.19 4-((6-Bromo-2-methoxyquinolin-3-yl)methyl)-6-(ethylthio)-*N*,*N*-dimethylpyridin-2-amine (**AB-19**)

To a glass tube was charged methyl 2-chloro-6-(dimethylamino)isonicotinate (WO 2010/100475) (2.44 g, 11.40 mmol), *rac*-bis(diphenylphosphino)-1,1′-binaphthol (0.71 g, 1.140 mmol) and cesium carbonate (4.43 g, 13.60 mmol) under continuous nitrogen flow. Anhydrous toluene (30 mL) was added. The mixture was purged with nitrogen for 5 min Palladium acetate (0.26 g, 1.158 mmol) was added and the mixture was purged again with nitrogen. Ethanethiol (1.0 mL, 13.60 mmol) was added and the mixture was sealed in the tube and heated at 150 °C for 22 h. The mixture was filtered through Celite, washing with EtOAc. The filtrate was concentrated in the fume hood by heating the solution while purging with air. A crude orange solid was obtained. Flash chromatography of the product using 2–5% diethyl ether in hexanes provided the product methyl 2-(dimethylamino)-6-(ethylthio)isonicotinate (2.42 g, 88%) as a yellow crystalline solid. ^1^H NMR (CDCl_3_) *δ* 6.96 (1H, d, *J* = 1.0 Hz), 6.73 (1H, d, *J* = 1.0 Hz), 3.89 (3H, s), 3.15 (2H, q, *J* = 7.3 Hz), 3.12 (6H, s), 1.38 (3H, t, *J* = 7.3 Hz). Found: [M + H] = 241.5.

To a solution of methyl 2-(dimethylamino)-6-(ethylthio)isonicotinate (1.64 g, 6.824 mmol) in freshly distilled THF (66 mL) was added at 2 °C under nitrogen lithium aluminium hydride (0.31 g, 8.189 mmol) in 3 batches. The mixture was stirred at 2 °C for 15 min then at 20 °C for 1 h. The mixture was quenched cautiously with water at 2 °C until gas evolution ceased. 1 M NaOH (20 mL) was added. The mixture was stirred for 5 min, then decanted leaving the aluminium salts which were filtered through Celite. The aqueous mixture was partitioned between water and EtOAc. The aqueous phase was extracted with EtOAc (3x). The combined organic extract was washed with brine, dried (Na_2_SO_4_) and concentrated to give the crude product as a brown oil. The crude product was purified by flash chromatography using a 4:1 mixture of hexanes/EtOAc to afford the product (2-(dimethylamino)-6-(ethylthio)pyridin-4-yl)methanol (1.30 g, 90%) as a brown oil. ^1^H NMR (CDCl_3_) *δ* 6.43 (1H, s), 6.18 (1H, d, *J* = 0.8 Hz), 4.56 (2H, d, *J* = 5.0 Hz), 3.14 (2H, q, *J* = 7.4 Hz), 3.08 (6H, s), 1.67 (1H, br t, *J* = 5.8 Hz), 1.57 (3, s), 1.37 (3H, t, *J* = 7.3 Hz). Found: [M + H] = 213.5.

To a solution of (2-(dimethylamino)-6-(ethylthio)pyridin-4-yl)methanol (0.60 g, 2.84 mmol) in anhydrous DCM (10 mL) was added at 2 °C under nitrogen triethylamine (0.59 mL, 4.26 mmol) dropwise, followed by mesyl chloride (0.26 mL, 3.41 mmol). The mixture was stirred from 2 °C to 5 °C over 1 h. The mixture was quenched with sat. aq. NaHCO_3_ solution. The aqueous mixture was extracted with DCM (3x). The combined extract was washed with brine, dried (MgSO_4_) and concentrated to afford the crude product as a light brown oil. The crude intermediate was dissolved in acetone (20 mL). Lithium bromide (0.99 g, 11.36 mmol) was added and the suspension was stirred at 20 °C for 2.5 h. Flash chromatography using 98:2 hexanes/Et_2_O furnished the product 4-(bromomethyl)-6-(ethylthio)-*N*,*N*-dimethylpyridin-2-amine as a brown solid. The reaction was repeated on a 3.29 mmol scale and the products were combined. Total yield = 1.39 g, 82%. ^1^H NMR (CDCl_3_) *δ* 6.46 (1H, d, *J* = 1.2 Hz), 6.15 (1H, d, *J* = 0.8 Hz), 4.24 (2H, s), 3.13 (2H, q, *J* = 7.2 Hz), 3.08 (6H, s), 1.37 (3H, t, *J* = 7.2 Hz). Found: [M + H] = 275.5

A mixture of (6-bromo-2-methoxyquinolin-3-yl)boronic acid (1.42 g, 5.05 mmol), 4-(bromomethyl)-6-(ethylthio)-*N*,*N*-dimethylpyridin-2-amine (1.39 g, 5.05 mmol) and cesium carbonate (3.29 g, 10.09 mmol) in a mixture of toluene (14 mL) and DMF (7 mL) was purged with nitrogen. Pd(PPh_3_)_4_ (0.29 g) was added. The mixture was purged again with nitrogen and heated at 85 °C under nitrogen for 2.5 h. The mixture was partitioned between water and EtOAc. The aqueous mixture was extracted with EtOAc (2x). The extract was washed with water, brine, dried (MgSO4) and concentrated to afford the crude product as an orange oil which was chromatographed using 2–5% Et_2_O in hexanes as eluent to yield the product as a light yellow solid. Recrystallisation from DCM/MeOH provided 4-((6-bromo-2-methoxyquinolin-3-yl)methyl)-6-(ethylthio)-*N*,*N*-dimethylpyridin-2-amine (**AB-19**) (1.25 g, 57%) as a white solid. ^1^H NMR (CDCl_3_) *δ* 7.79 (1H, d, *J* = 2.4 Hz), 7.70 (1H, d, *J* = 8.8 Hz), 7.64 (1H, dd, *J* = 2.4, 9.2 Hz), 7.56 (1H, s), 6.34 (1H, s), 6.06 (1H, s), 4.09 (3H, s), 3.86 (2H, s), 6.15 (2H, q, *J* = 7.2 Hz), 3.07 (6H, s), 1.39 (3H, t, *J* = 7.2 Hz). Found [M + H] = 432.1

##### 4.1.1.20 6-Bromo-3-((3-fluoro-2-methoxypyridin-4-yl)methyl)-2-methoxyquinoline (**AB-20**)

To a solution of *n*-BuLi (26.1 mL, 52.09 mmol) in THF (70 mL, dist. Na) at −78 °C was added consecutively *N*,*N*-diisopropylamine (7.30 mL, 52.09 mmol) and 2,3-difluoropyridine (5.00 g, 43.41 mmol).The resultant mixture was stirred at −78 °C for 1 h, and then poured on crushed dry ice (excess). The reaction was allowed to warm to 20 °C for 1 h, and after evaporation of excess dry ice and THF, the residue was taken up into water (100 mL) and washed with EtOAc (2 × 50 mL). The aqueous layer was then acidified to pH 1 and extracted with EtOAc (2 × 100 mL). The combined organic extracts were dried and evaporated to afford 2,3-difluoroisonicotinic acid (2.39 g, 35%). ^1^H NMR (CDCl_3_) *δ* 15.3–13.2 (1H, br s), 8.14 (1H, dd, *J* = 1.2, 5.0 Hz), 7.70 (1H, dd, *J* = 4.8, 4.8 Hz). Found: [M−H] = 158.5.

Sodium (0.79 g, 33.09 mmol) was added portion wise to MeOH (60 mL) over 0.5 h. 2,3-difluoroisonicotinic acid (2.39 g, 15.04 mmol) was then added and the reaction refluxed for 2 h. The solution was cooled and the solvent evaporated. The residue was taken up into water (100 mL) and washed with EtOAc (2 × 50 mL). The aqueous layer was then acidified to pH 1 and extracted with EtOAc (2 × 100 mL). The combined organic extracts were dried and evaporated to afford the product 3-fluoro-2-methoxyisonicotinic acid (2.23 g, 87%). ^1^H NMR (DMSO‑*d*_6_) *δ* 14.6–12.8 (1H, br s), 8.05 (1H, d, *J* = 5.2 Hz), 7.28 (1H, dd, *J* = 4.7, 4.9 Hz), 3.97 (3H, s). Found: [M−H] = 170.5.

Borane − dimethylsulfide complex (2.47 mL, 26.07 mmol) and trimethyl borate (2.96 mL, 26.07 mmol) were added to a solution of 3-fluoro-2-methoxyisonicotinic acid (13.03 mmol) in THF (80 mL, dist. Na) at 0 °C, and the solution warmed to 20 °C and stirred overnight. The mixture was then cooled to 0 °C, and quenched with methanol (10 mL). The solvent was then evaporated and the residue was partitioned between EtOAc and water. The organic layer was then dried and evaporated to afford the product (3-fluoro-2-methoxypyridin-4-yl)methanol (1.89 g, 92%). ^1^H NMR (CDCl_3_) *δ* 7.92 (1H, d, *J* = 5.2 Hz), 7.02 (1H, dd, *J* = 4.5, 5.1 Hz), 4.81 (2H, s), 4.03 (3H, s). Found: [M + H] = 158.5.

To a solution of (3-fluoro-2-methoxypyridin-4-yl)methanol (1.89 g, 12.03 mmol) and triethylamine (2.52 mL, 18.05 mmol) in DCM (30 mL, anhydrous) at 20 °C was added mesyl chloride (1.12 mL, 14.44 mmol) dropwise. After 15 min, the reaction was diluted with DCM (20 mL) and the organic layer washed with sat. NaHCO_3_, dried and evaporated. The residue was dissolved in acetone (60 mL, anhydrous), lithium bromide (excess) added, and the mixture heated at reflux for 30 min. The solution was then cooled and the solvent evaporated, and the residue partitioned between EtOAc and water. The aqueous layer was extracted twice with EtOAc and the organic layer was dried and evaporated to give the product 4-(bromomethyl)-3-fluoro-2-methoxypyridine (2.20 g, 83%). ^1^H NMR (CDCl_3_) *δ* 7.90 (1H, d, *J* = 5.2 Hz), 6.90 (1H, dd, *J* = 4.8, 4.9 Hz), 4.43 (2H, s), 4.02 (3H, s). Found: [M + H] = 220.2

A mixture of (6-bromo-2-methoxyquinolin-3-yl)boronic acid (2.56 g, 9.09 mmol), 4-(bromomethyl)-3-fluoro-2-methoxypyridine (2.20 g, 10.00 mmol) and cesium carbonate (5.92 g, 18.18 mmol) in toluene (45 mL, anhydrous) and DMF (22.5 mL, anhydrous) was purged with nitrogen. Pd(PPh_3_)_4_ (0.42 g, 0.363 mmol) was then added, the mixture purged with nitrogen, then heated to 80 °C under nitrogen for 4 h. The reaction was partitioned between EtOAc and water and the organic fraction was dried and evaporated. Column chromatography (19:1 hexanes/EtOAc) gave the product 6-bromo-3-((3-fluoro-2-methoxypyridin-4-yl)methyl)-2-methoxyquinoline (**AB-20**) (1.76 g, 51%). ^1^H NMR (CDCl_3_) *δ* 7.82 (1H, d, *J* = 5.2 Hz), 7.79 (1H, d, *J* = 2.1 Hz), 7.69 (1H, d, *J* = 8.8 Hz), 7.6 4 (1H, dd, *J* = 2.2, 8.9 Hz), 7.60 (1H, s), 6.69 (1H, dd, *J* = 4.8, 5.0 Hz), 4.07 (3H, s), 4.06 (2H, s), 4.03 (3H, s). Found: [M + H] = 377.2

#### 4.1.2 Scheme 2. New C/D units

##### 4.1.2.1 1-(2,6-Dimethoxypyridin-4-yl)-3-(dimethylamino)propan-1-one (**IIIa**)

Oxalyl chloride (1.34 mL, 15.8 mmol) was added to a suspension of 2,6-dimethoxyisonicotinic acid (2.41 g, 13.2 mmol) in DCM (70 mL) and DMF (0.20 mL, 2.6 mmol) at 20 °C. The mixture was stirred for 1 h to give a colourless solution which was cooled to 0 °C. *N,O-*dimethylhydroxylamine hydrochloride (1.42 g, 14.6 mmol) and pyridine (3.51 mL, 28.9 mmol) were added sequentially and the mixture was stirred at 20 °C for 18 h, then partitioned between EtOAc and sat. aq. NaHCO_3_. Column chromatography with hexanes:EtOAc (2:1) gave *N*,2,6-trimethoxy-*N*-methylisonicotinamide (**II (Z = Me)**, 2.49 g, 83%). ^1^H NMR (CDCl_3_) *δ* 6.47 (s, 2H), 3.93 (s, 6H), 3.58 (br s, 3H), 3.32 (s, 3H). Found: [M + H] = 227.2.

Vinylmagnesium bromide (42.5 mL of a 1 M solution in THF, 42.5 mmol) was added to a solution of *N*,2,6-trimethoxy-*N*-methylisonicotinamide (**II (Z = Me)**, 4.81 g, 21.3 mmol) in dry THF (200 mL) at 0 °C. The brown solution was warmed to 20 °C for 1 h then dimethylamine (42.5 mL of a 2 M solution in THF, 85 mmol) and water (40 mL) were added. The solution was stirred at 20 °C for 1 h, then partitioned between EtOAc and water. The solution was dried and evaporated and column chromatography with DCM:MeOH (95:5) eluted impurities while DCM:MeOH (9:1) gave 1-(2,6-dimethoxypyridin-4-yl)-3-(dimethylamino)propan-1-one (**IIIa**) (2.90 g, 57%) as a brown oil. ^1^H NMR (CDCl_3_) *δ* 6.74 (s, 2H), 3.95 (s, 6H), 3.06 (t, *J* = 7.0 Hz, 2H), 2.72 (t, *J* = 7.0 Hz, 2H), 2.27 (s, 6H). Found: [M + H] = 239.1

##### 4.1.2.2 1-(2,6-Diethoxypyridin-4-yl)-3-(dimethylamino)propan-1-one (**IIIb**)

Oxalyl chloride (0.73 mL, 8.6 mmol) was added to a suspension of 2,6-diethoxyisonicotinic acid (1.52 g, 7.20 mmol) in DCM (50 mL, anhydrous) and DMF (0.20 mL, 2.6 mmol) at 20 °C. The mixture was stirred at 20 °C for 1 h to give a colourless solution, which was then cooled to 0 °C. *N,O-*dimethylhydroxylamine hydrochloride (0.77 g, 17.89 mmol) and pyridine (1.92 mL, 23.7 mmol) were added sequentially and the mixture was stirred at 20 °C for 18 h, then partitioned between EtOAc and sat. aq. NaHCO_3_. Column chromatography using 3:1 hexanes:EtOAc gave 2,6-diethoxy-*N*-methoxy-*N*-methylisonicotinamide (**II (Z = Et)**, 1.26 g, 69%). ^1^H NMR (CDCl_3_) *δ* 6.43 (s, 2H), 4.33 (q, *J* = 7.1 Hz, 2H), 3.59 (br s, 3H), 3.32 (s, 3H), 1.39 (t, *J* = 7.1 Hz, 3H). Found: [M + H] = 255.1

Vinylmagnesium bromide (14.6 mL of a 1 M solution in THF, 14.6 mmol) was added to a solution of 2,6-diethoxy-*N*-methoxy-*N*-methylisonicotinamide (**II (Z = Et)**, 1.23 g, 4.85 mmol) in dry THF (50 mL) at 0 °C. The brown solution was warmed to 20 °C for 1 h then a solution of 2 M dimethylamine in THF (14.6 mL, 29.2 mmol) and water (10 mL) were added. The solution was stirred at 20 °C for 1 h, then partitioned between EtOAc and water. The solution was dried and evaporated to give 1-(2,6-diethoxypyridin-4-yl)-3-(dimethylamino)propan-1-one (**IIIb)** (1.24 g, 96%) as a brown oil. ^1^H NMR (CDCl_3_) *δ* 6.71 (s, 2H), 4.34 (q, *J* = 7.1 Hz, 2H), 3.05 (t, *J* = 7.0 Hz, 2H), 2.72 (t, *J* = 7.0 Hz, 2H), 2.26 (s, 6H), 1.40 (t, *J* = 7.0 Hz, 3H). Found: [M + H] = 267.2

##### 4.1.2.3 1-(2,6-Dimethoxypyridin-4-yl)-3-(methoxy(methyl)amino)propan-1-one (**IV**)

Vinylmagnesium bromide (62.7 mL of a 1 M solution in THF, 62.7 mmol) was added to a solution of *N*,2,6-trimethoxy-*N*-methylisonicotinamide (**II (Z = Me)**, 6.75 g, 29.8 mmol) in dry THF (150 mL) at 0 °C. The yellow/orange solution was warmed to 20 °C for 1 h and water (30 mL) were added. The solution was stirred at 20 °C for 24 h, then partitioned between EtOAc and water. The solution was dried and evaporated and column chromatography with hexanes:EtOAc (9:1) gave 1-(2,6-dimethoxypyridin-4-yl)-3-(methoxy(methyl)amino)propan-1-one (**IV**) (5.50 g, 72%) as an oil. ^1^H NMR (CDCl_3_) *δ* 6.76 (s, 2H), 3.96 (s, 6H), 3.46 (s, 3H), 3.16 (t, *J* = 6.6 Hz, 2H), 3.04 (t, *J* = 6.5 Hz, 2H), 2.61 (s, 3H). Found: [M + H] = 255.6.

##### 4.1.2.4 1-(2,6-Dimethoxypyridin-4-yl)-3-morpholinopropan-1-one (**V**)

Vinylmagnesium bromide (18.6 mL of a 1 M solution in THF, 18.6 mmol) was added to a solution of *N*,2,6-trimethoxy-*N*-methylisonicotinamide (**II (Z = Me)**, 2.00 g, 8.80 mmol) in dry THF (30 mL) at 0 °C. The yellow/orange solution was warmed to20 ^o^C for 1 h then morpholine (3.23 mL, 37.1 mmol) then water (10 mL) were added. The solution was stirred at 20 °C for 1 h, the solvent removed in *vacuo*, and the resultant mixture then partitioned between EtOAc and water. The solution was dried and evaporated to afford 1-(2,6-dimethoxypyridin-4-yl)-3-morpholinopropan-1-one (**V**) (2.40 g, 97%) as an oil. ^1^H NMR (CDCl_3_) *δ* 6.73 (s, 2H), 3.95 (s, 6H), 3.70 (t, *J* = 4.4 Hz, 4H), 3.08 (t, *J* = 7.1 Hz, 2H), 2.79 (t, *J* = 7.4 Hz, 2H), 2.48 (t, *J* = 4.4 Hz, 4H). Found: [M + H] = 281.6.

##### 4.1.2.5 1-(2,6-Dimethoxypyridin-4-yl)-3-(1H-imidazol-1-yl)propan-1-one (**VI**)

Vinylmagnesium bromide (0.93 mL of a 1 N solution in THF, 0.93 mmol) was added to a solution of *N*,2,6-trimethoxy-*N*-methylisonicotinamide (**II (Z = Me)**,0.10 g, 0.44 mmol) in dry THF (3 mL) at 0 °C. The yellow/orange solution was warmed to 20 °C for 1 h. The solvent was removed in *vacuo* and the residue partitioned between EtOAc and water, and the pH adjusted to 3 with 1 M HCl. The combined organic extracts were dried and the solvent removed in vacuo. The crude residue was redissolved in THF (3 mL), cooled to 0 °C, and imidazole (0.18 g, 2.65 mmol) was added followed by water (1 mL). The solution was then stirred at 20 °C for 1 h, then partitioned between EtOAc and water. The solution was dried and evaporated and column chromatography with hexanes:EtOAc (1:2) gave 1-(2,6-dimethoxypyridin-4-yl)-3-(1*H*-imidazol-1-yl)propan-1-one (**VI**) (0.045 g, 39%) as an oil. ^1^H NMR (CDCl_3_) *δ* 7.54 (s, 1H), 7.04 (s, 1H), 6.95 (s, 1H), 6.68 (s, 2H), 4.41 (t, *J* = 6.4 Hz, 2H), 3.95 (s, 6H), 3.35 (t, *J* = 6.4 Hz, 2H). Found: [M + H] = 262.6

##### 4.1.2.6 1-(2,6-Dimethoxypyridin-4-yl)-3-(1H-1,2,4-triazol-1-yl)propan-1-one (**VII**)

Vinylmagnesium bromide (27.9 mL of a 1 N solution in THF, 27.9 mmol) was added to a solution of *N*,2,6-trimethoxy-*N*-methylisonicotinamide (**II (Z = Me)**, 3.00 g, 13.3 mmol) in dry THF (30 mL) at 0 °C. The yellow/orange solution was warmed to 20 °C for 1.5 h. The solvent was removed in *vacuo* and the residue partitioned between chloroform and water, and the pH adjusted to 1 with 1 M HCl. The combined organic extracts were dried and the solvent removed in *vacuo*. The crude residue was redissolved in chloroform (150 mL) and 1*H*-1,2,4-triazole (2.67 g, 39.8 mmol) and the resultant mixture stirred at 60 °C for 3 h. The solution cooled to 20 °C and partitioned between chloroform and water. The solution was dried and evaporated and column chromatography with DCM:MeOH (99:1) gave 1-(2,6-dimethoxypyridin-4-yl)-3-(1H-1,2,4-triazol-1-yl)propan-1-one (**VII**) (1.54 g, 44%) as an oil. ^1^H NMR (CDCl_3_) *δ* 8.20 (s, 1H), 7.91 (s, 1H), 6.70 (s, 2H), 4.61 (t, *J* = 6.2 Hz, 2H), 3.94 (s, 6H), 3.51 (t, *J* = 6.1 Hz, 2H). Found: [M + H] = 263.6.

#### 4.1.3 Example of the synthesis of the bromo analogues of Table 1

##### 4.1.3.1 1-(6-Bromo-2-methoxyquinolin-3-yl)-1-(2,5-dimethoxypyridin-3-yl)-2-(2,6-dimethoxypyridin-4-yl)-4-(dimethylamino)butan-2-ol (**27**)

*n*-BuLi (2.92 mL of a 2 N solution in cyclohexane, 5.83 mmol) was added at −40 °C under dry nitrogen to a solution of dry diisopropylamine (0.813 mL, 5.83 mmol) in dry THF (6 mL) and the solution was stirred at this temperature for 10 min, then cooled to −78 °C. A solution of 6-bromo-3-((2,5-dimethoxypyridin-3-yl)methyl)-2-methoxyquinoline (**AB-10**) (1.90 g, 4.86 mmol) in dry THF (6 mL) was added dropwise and the mixture was stirred at −78 °C for 90 min, to give a dark, wine-red coloured solution. A solution of 1-(2,6-dimethoxypyridin-4-yl)-3-(dimethylamino)propan-1-one (**IIIa**) (1.15 g, 4.86 mmol) in dry THF (7 mL) was added and the reaction mixture was stirred at this temperature for 4 h. HOAc (0.90 mL) was added and the reaction mixture was warmed to 20 °C. Water (100 mL) was added and the mixture was extracted with EtOAc (2x). The combined organic extract was washed with sat. aq. NaHCO_3_ solution, and brine, then dried (Na_2_SO_4_) and the solvent removed under reduced pressure. The residue was purified by flash column chromatography. Elution with 0–10% MeOH/DCM afforded isomer A of **27** (1.11 g, 36%) followed by isomer B of **27** (1.03 g, 34%) as white solids.

Isomer A. ^1^H NMR (CDCl_3_, 400 MHz) *δ* 8.12 (d, *J* = 3.3 Hz, 2H), 7.82 (d, *J* = 2.2 Hz, 1H), 7.68 (d, *J* = 8.9 Hz, 1H), 7.60 (dd, *J* = 8.9, 2.2 Hz, 1H), 7.48 (d, *J* = 3.0 Hz, 1H), 6.56 (br s, 2H), 5.31 (s, 1H), 4.19 (s, 3H), 3.88 (s, 6H), 3.74 (s, 3H), 3.63 (s, 3H), 2.30–2.04 (m, 1H), 2.02–1.96 (m, 1H), 1.97 (s, 6H), 1.83–1.68 (m, 2H). Found: [M + H] = 627.8.

Isomer B. ^1^H NMR (CDCl_3_, 400 MHz) *δ* 8.63 (s, 1H), 7.78 (d, *J* = 1.7 Hz, 1H), 7.68 (d, *J* = 3.0 Hz, 1H), 7.55–7.47 (m, 3H), 6.56 (br s, 2H), 5.32 (s, 1H), 4.04 (s, 3H), 3.84 (s, 3H), 3.83 (s, 6H), 3.71 (s, 3H), 2.40–2.32 (m, 1H), 2.08 (s, 6H), 2.03–1.98 (m, 1H), 1.87–1.79 (m, 1H), 1.76–1.70 (m, 1H). Found: [M + H] = 627.8.

The mixture was resolved into its four optical isomers using preparative supercritical fluid HPLC at BioDuro LLC (Beijing). The data in Table 1 are for the most active *R*,*S*-diastereomers. The other 6-bromo compounds in Table I were prepared and purified similarly.

#### 4.1.4 Example of cyanation reaction to give the cyano compounds of Table 1

##### 4.1.4.1 hydroxybutyl)-2-methoxyquinoline-6-carbonitrile (**28**)

A solution of compound **27** (Table 1) (0.61 g, 0.969 mmol) in DMF (6 mL, anhydrous) was purged with nitrogen and heated to 55 °C for 10 min. Tri(o-tolyl)phosphine (0.044 g, 0.145 mmol), zinc dust (0.006 g, 0.097 mmol) and tris(dibenzylideneacetone)dipalladium(0) (0.067 g, 0.073 mmol) were then added, and the reaction was again purged with nitrogen and heated for another 10 min at 55 °C. Zinc cyanide (0.063 g, 0.533 mmol) was then added and the reaction mixture was heated to 65 °C for 4 h. The reaction was diluted with water and extracted with EtOAc three times. The organic layer was washed with brine three times, dried and evaporated. Column chromatography with 1:1 hexane/EtOAc followed by 1:3 hexane/EtOAc afforded **28** (0.41 g, 74%); isomer A as a white solid. ^1^H NMR (CDCl_3_, 400 MHz) *δ* 8.23 (s, 1H), 8.10 (d, *J* = 3.0 Hz, 1H), 8.05 (d, *J* = 1.7 Hz, 1H), 7.86 (d, *J* = 8.7 Hz, 1H), 7.71 (dd, *J* = 8.6, 1.8 Hz, 1H), 7.49 (d, *J* = 3.0, 1H), 6.56 (br s, 2H), 5.31 (s, 1H), 4.23 (s, 3H), 3.89 (s, 6H), 3.74 (s, 3H), 3.63 (s, 3H), 2.31–2.24 (m, 1H), 2.02–1.96 (m, 1H), 1.97 (s, 6H), 1.78–1.68 (m, 2H). Found: [M + H] = 574.6.

Followed by isomer B, (7%), foamy white solid. ^1^H NMR (CDCl_3_, 400 MHz) *δ* 8.76 (s, 1H), 8.01 (d, *J* = 1.7 Hz, 1H), 7.70–7.60 (m, 3H), 7.48 (d, *J* = 3.0 Hz, 1H), 6.55 (br s, 2H), 5.30 (s, 1H), 4.04 (s, 3H), 3.88 (s, 3H), 3.83 (s, 6H), 3.72 (s, 3H), 2.32–2.25 (m, 1H), 2.05 (s, 6H), 2.04–2.00 (m, 1H), 1.88–1.80 (m, 1H), 1.71–1.64 (m, 1H). Found: [M + H] = 574.6.

The mixtures were resolved into their four optical isomers using preparative supercritical fluid HPLC at BioDuro LLC (Beijing). The data in Table 1 are for the most active *R*,*S*-diastereomers. The other 6-cyano compounds in Table I were prepared and purified similarly.

## Appendix A Supplementary data

### MOL files

The following ZIP file contains the MOL files of the most important compounds referred to in this article.

MOL files ZIP file containing the MOL file of the most important compounds in this article
