# Supplementary material for: 3,5-Dialkoxypyridine analogues of bedaquiline are potent antituberculosis agents with minimal inhibition of the hERG channel
Source: Bioorg Med Chem. 2019 Apr 1;27(7):1292–307. doi: 10.1016/j.bmc.2019.02.026 (PMC6467547; doi:10.1016/j.bmc.2019.02.026)
Supplement: Supplementary Data 1 [file mmc1.docx]

Supplementary Material for:

**3,5-Dialkoxypyridine analogs of bedaquiline are potent antitubercular agents with minimal inhibition of the hERG channel**

Hamish S. Sutherland, Amy S.T. Tong, Peter J. Choi, Adrian Blaser, Daniel Conole, Scott G. Franzblau^c^, Manisha U; Lotlikar, Christopher B. Cooper, Anna M. Upton, William A. Denny, Brian D. Palmer

**Table of Contents**

| **Topic** | **Pages** |
| --- | --- |
| Scheme 1: synthesis of new bedaquiline analogues | 1 |
| Table 1: list of new A/B units | 2 |
| Syntheses of new A/B units | 2-28 |
| Scheme 2: Synthesis of dialkoxypyridyl Mannich bases (C/D units | 29 |
| Table 2: list of new A/B units | 29-32 |
| Example synthesis of the compounds of Table 1 | 32-35 |

**Scheme 1: synthesis of new bedaquiline analogues**

Reagents and conditions: (i) LiTMP, THF, -75 ^o^C, 1.5 h then appropriate aldehyde B, -75 ^o^C, 4 h; (ii) MsCl, Et_3_N, DMF, then NaBH_4_; (iii) LDA, THF, -75 ^o^C, 1.5 h then the appropriate ketone C/D; (iv) Zn/Zn(CN)_2_, Pd_2_(dba)_3_/P(o-tol)_3_, DMF, 50 ^o^C.

**Table 1: New A/B units**

| **Z** | **No** |
| --- | --- |
| 2,3-(CH_2_)_3_- | AB-1 |
| 2,3-(CH_2_)_4_- | AB-2 |
| 2-F, 3-Me | AB-3 |
| 3-F, 4-OMe | AB-4 |
| 2,3-OCH_2_O- | AB-5 |
| 2,3-OCH=CH- | AB-6 |
| 3-aza, 2-OMe, 5-OiPr | AB-7 |
| 3-aza, 2,4,5-triOMe | AB-8 |
| 3-aza, 4-NEt_2_ | AB-9 |
| 4-aza, 2,5-diOMe | AB-10 |
| 4-aza, 2-OMe, 5-OiPr | AB-11 |
| 4-aza, 3-OMe, 5-NMe_2_ | AB-12 |
| 4-aza, 3-OEt, 5-NMe_2_ | AB-13 |
| 4-aza, 2,3,5-triOMe | AB-14 |
| 4-aza, 3-OEt, 5-OiPr | AB-15 |
| 4-aza, 3-OMe, 5-OcBu | AB-16 |
| 4-aza, 3,5-diSMe | AB-17 |
| 4-aza, 3,5-diSEt | AB-18 |
| 4-aza, 3-SEt, 5-NMe_2_ | AB-19 |
| 4-aza, 2-F, 3-OMe | AB-20 |

**6-Bromo-3-((2,3-dihydro-1*H*-inden-4-yl)methyl)-2-methoxyquinoline (AB-1)**

A solution of 2,3-dihydro-1*H*-indene-4-carboxylic acid (**S1**) (6.12 g, 28.4 mmol) in THF (100 mL, dist. Na) at 0 °C was treated with lithium aluminium hydride (8.09 mL, 85.3 mmol) in small portions. The reaction mixture was stirred at 0 °C for 30 min and then at r.t. for a further 24 h. The reaction mixture was washed with water (100 mL) and extracted with EtOAc (3 x 50 mL). The organic phase was dried with Na_2_SO_4_ and concentrated under reduced pressure to obtain 2,3-dihydro-1*H*-inden-4-yl)methanol (**S2**) as a yellow oil (2.45 g, 99%). ^1^H NMR (CDCl_3_) δ 7.22-7.12 (m, 3H), 4.67 (s, 2H), 2.93 (t, J = 7.6 Hz, 2H), 2.91 (t, J = 7.4 Hz, 2H), 2.09 (p, J = 7.6 Hz, 2H). Found: [M+H-18]=131.5.

To a solution of **S2** (4.33 g, 36.4 mmol) in DCM (50 mL) at 0 °C was added thionyl chloride (2.45 g, 16.5 mmol). The reaction mixture was stirred at r.t. for 24 h and solvent was removed under reduced pressure. The residue was diluted with DCM (100 mL) and quenched with ice-water (100 mL). The organic phase was washed with sat. aq. NaHCO_3_, dried with Na_2_SO_4_ and concentrated to give a yellow residue. Purification by flash column chromatography using hexanes:EtOAc (1:1) gave 4-(chloromethyl)-2,3-dihydro-1*H*-indene (**S3**) as a colourless oil (2.36 g, 86%). ^1^H NMR (CDCl_3_) δ 7.22-7.13 (m, 3H), 4.59 (s, 2H), 2.99 (t, J = 7.5 Hz, 2H), 2.94 (t, J = 7.5 Hz, 2H), 2.11 (p, J = 7.6 Hz, 2H). Found: [M+H]=167.5.

A mixture of (6-bromo-2-methoxyquinolin-3-yl)boronic acid (3.69 g, 12.9 mmol), **S3** (2.36 g, 14.2 mmol) and Cs_2_CO_3_ (9.67 g, 29.7 mmol) in toluene:DMF (60 mL, 2:1) was degassed under N_2_, then Pd(PPh_3_)_4_ (0.745 g, 0.645 mmol), was added and the mixture was heated at 90 °C for 3 h. The reaction mixture was cooled to r.t., filtered through a plug of Celite, water (150 mL) was added the mixture was extracted with EtOAc (3 x 100 mL). The combined organic layers were washed with brine (100 mL), dried over Na_2_SO_4_, filtered and concentrated under reduced pressure to obtain a yellow residue. Purification by flash column chromatography using hexanes:EtOAc (9:1) gave **AB-1** as a yellow oil (4.70 g, 99%). ^1^H NMR (CDCl_3_) δ 7.71 (d, J = 2.2 Hz, 1H), 7.68 (d, J = 8.9 Hz, 1H), 7.60 (dd, J = 8.9, 2.2 Hz, 1H), 7.33 (s, 1H), 7.18-7.11 (m, 2H), 6.95 (d, J = 7.2 Hz, 1H), 4.11 (s, 3H), 3.98 (s, 2H), 2.96 (t, J = 7.5 Hz, 2H), 2.79 (t, J = 7.4 Hz, 2H), 2.04 (p, J = 7.6 Hz, 2H). Found: [M+H]=368.5.

**6-Bromo-2-methoxy-3-((5,6,7,8-tetrahydronaphthalen-1-yl)methyl)quinolone (AB-2)**

A solution of 1-tetrahydronaphthoic acid (**S4**) (5.70 g, 32.3 mmol) in THF (100 mL, dist. Na) at 0 °C was treated with lithium aluminium hydride (2.46 g, 64.7 mmol) in small portions. The reaction mixture was stirred at 0 °C for 30 min and then stirred for further 24 h at r.t. The reaction mixture was washed with water (100 mL) and extracted with EtOAc (3 x 50 mL). The organic phase was dried with Na_2_SO_4_ and concentrated under reduced pressure to obtain (5,6,7,8-tetrahydronaphthalen-1-yl)methanol (**S5**) as colourless oil (5.23 g, 99%). ^1^H NMR (CDCl_3_) δ 7.18 (d, J = 7.4 Hz, 1H), 7.11 (d, J = 7.5 Hz, 1H), 7.05 (d, J = 7.6 Hz, 1H), 4.67 (s, 2H), 2.80 (t, J = 6.2 Hz, 2H), 2.76 (t, J = 6.4 Hz, 2H), 1.88-1.77 (m, 4H). Found: [M+H-18]=145.5.

To a solution of **S5** (5.24 g, 32.3 mmol) in DCM (200 mL) at 0 °C was added thionyl chloride (8.45 g, 71.1 mmol). The reaction mixture was stirred at r.t. for 24 h, then solvent was removed under reduced pressure. The residue was diluted with DCM (100 mL) and quenched with ice-water (100 mL). The organic phase was washed with sat. aq. NaHCO_3_, dried with Na_2_SO_4_ and concentrated to give 5-(chloromethyl)-1,2,3,4-tetrahydronaphthalene (**S6**) as a brown oil (3.25 g, 56%). ^1^H NMR (CDCl_3_) δ 7.16-7.05 (m, 3H), 4.59 (s, 2H), 2.86 (t, J = 6.3 Hz, 2H), 2.79 (t, J = 6.4 Hz, 2H), 1.88-1.77 (m, 4H). Found: [M+H]=181.6.

A mixture of (6-bromo-2-methoxyquinolin-3-yl)boronic acid (4.69 g, 16.4 mmol), **S6** (3.25 g, 18.0 mmol) and Cs_2_CO_3_ (12.29 g, 37.7 mmol) in toluene:DMF (60 mL, 2:1) was degassed under N_2_, then Pd(PPh_3_)_4_ (0.948 g, 0.82 mmol) was added and the mixture was heated at 90 °C for 3 h. The reaction mixture was cooled to r.t., filtered through a plug of Celite, water (150 mL) was added and the mixture was extracted with EtOAc (3 x 100 mL). The combined organic layers were washed with brine (100 mL), dried over Na_2_SO_4_, filtered and concentrated under reduced pressure to obtain a yellow residue. Purification by flash column chromatography using hexanes:EtOAc (9:1) gave 6-bromo-2-methoxy-3-((5,6,7,8-tetrahydronaphthalen-1-yl)methyl)quinolone (**AB-2**) as a white solid (4.64 g, 74%). ^1^H NMR (CDCl_3_) δ 7.71-7.69 (m, 2H), 7.60 (dd, J = 8.9, 2.2 Hz, 1H), 7.23 (s, 1H), 7.12-7.04 (m, 2H), 6.92 (d, J = 6.9 Hz, 1H), 4.11 (s, 3H), 3.96 (s, 2H), 2.83 (bs, 2H), 2.57 (bs, 2H), 1.76 (p, J = 3.5 Hz, 4H). Found: [M+H]=382.1.

**6-Bromo-3-(2-fluoro-3-methylbenzyl)-2-methoxyquinoline (AB-3)**

A mixture of (6-bromo-2-methoxyquinolin-3-yl)boronic acid (3.00 g, 10.5 mmol), 1-(bromomethyl)-2-fluoro-3-methylbenzene (**S7**) (4.24 g, 20.9 mmol) and Cs_2_CO_3_ (7.87 g, 24.2 mmol) in toluene:DMF (60 mL, 2:1) was degassed under N_2_, then Pd(PPh_3_)_4_ (0.607 g, 0.525 mmol) was added, and the mixture was heated at 90 °C for 2 h. The reaction mixture was cooled to r.t., filtered through a plug of Celite, water (150 mL) was added and the mixture was extracted with EtOAc (3 x 100 mL). The organic layer was washed with brine (100 mL), dried over Na_2_SO_4_, filtered and concentrated under reduced pressure to obtain a yellow residue. Purification by flash column chromatography using hexanes:EtOAc (9:1) gave **AB-3** as a white solid (2.87 g, 76%). ^1^H NMR (CDCl_3_) δ 7.75 (d, J = 2.2 Hz, 1H), 7.67 (d, J = 8.8 Hz, 1H), 7.60 (dd, J = 8.9, 2.2 Hz, 1H), 7.50 (s, 1H), 7.11-6.96 (m, 3H), 4.09 (s, 3H), 4.03 (s, 2H), 2.28 (d, J = 2.1 Hz, 3H). Found: [M+H]=360.6.

**6-Bromo-3-(3-fluoro-4-methoxybenzyl)-2-methoxyquinoline (AB-4)**

Borane−dimethylsulfide complex (2.79 mL, 29.40 mmol) and trimethyl borate (3.34 mL, 29.40 mmol) were added to a solution of 3-fluoro-4-methoxybenzoic acid (**S8**) (2.50 g, 14.69 mmol) in THF (80 mL, dist. Na) at 0 °C, and the solution warmed to r.t. and stirred overnight. The mixture was then cooled to 0 °C, and quenched with MeOH (10 mL). The solvent was then evaporated and the residue was partitioned between EtOAc and water. The organic layer was then dried and evaporated to afford (3-fluoro-4-methoxyphenyl)methanol (**S9**), ( 2.35 g, 100%. ^1^H NMR (CDCl_3_) δ 7.11 (1H, dd, J = 2.0, 11.9 Hz), 7.06 (1H, ddd, J = 0.9, 2.0, 9.1 Hz), 6.94 (1H, dd, J = 8.4, 8.4 Hz), 4.61 (2H, s), 3.89 (3H, s), 1.73 (1H, s). Found: [M-OH] =139.7.

To a solution of **S9** (2.35 g, 15.05 mmol) and triethylamine (3.15 mL, 22.58 mmol) in DCM (50 mL, anhydrous) at r.t. was added mesyl chloride (1.414 mL, 18.06 mmol) dropwise. After 15 min, the reaction was diluted with DCM (50 mL) and the organic layer washed with sat. aq. NaHCO_3_, dried and evaporated. The residue was dissolved in acetone (100 mL, anhydrous), lithium bromide (excess) added, and the mixture heated at reflux for 30 min. The solution was then cooled and the solvent evaporated, and the residue partitioned between EtOAc and water. The aqueous layer was extracted twice with EtOAc and the organic layer was dried and evaporated to afford 4-(bromomethyl)-2-fluoro-1-methoxybenzene (**S10**), (3.00 g, 91%. ^1^H NMR (CDCl_3_) δ 7.16-7.07 (2H, m), 6.90 (1H, dd, J = 8.3, 8.4 Hz), 4.45 (2H, s), 3.89 (3H, s).

A mixture of (6-bromo-2-methoxyquinolin-3-yl)boronic acid (3.51 g, 12.45 mmol), **S10** (3.00 g, 13.69 mmol) and cesium carbonate (8.93 g, 27.40 mmol) in toluene (60 mL, anhydrous) and DMF (30 mL, anhydrous) was purged with nitrogen. Pd(PPh_3_)_4_ (0.58 g, 0.50 mmol) was then added, the mixture purged with nitrogen then heated to 80 °C under nitrogen for 5 hours. The reaction was partitioned between EtOAc and water and the organic fraction was dried and evaporated. Column chromatography (19:1 hexanes/EtOAc) gave **6**-bromo-3-(3-fluoro-4-methoxybenzyl)-2-methoxyquinoline (**AB-4**), (2.10 g, 41%. ^1^H NMR (CDCl_3_) δ 7.76 (1H, d, J = 2.2 Hz), 7.69 (1H, d, J = 12.3 Hz), 7.62 (1H, dd, J = 2.2, 8.9 Hz), 7.49 (1H, s), 7.00-6.86 (3H, m), 4.07 (3H, s), 3.95 (2H, s), 3.88 (3H, s). Found: [M+H]=376.0

**3-(benzo[d][1,3]dioxol-4-ylmethyl)-6-bromo-2-methoxyquinoline (AB-5)**

A mixture of **(**6-bromo-2-methoxyquinolin-3-yl)boronic acid (1.5 g, 5.32 mmol), 4-(bromomethyl)benzo[d][1,3]dioxole (**S11**) (1.2 g, 5.59 mmol) and 2M Na_2_CO_3_ (5 mL) in DME (25 mL) was purged with nitrogen. Pd(PPh_3_)_4_ (0.31 g, 0.27 mmol) was added, the mixture was purged with nitrogen then heated to 95 °C under nitrogen for 4 h. The reaction was partitioned between EtOAc and water and the organic fraction was dried and evaporated. Column chromatography with hexanes:EtOAc (100:0 to 95:5) gave 3-(benzo[d][1,3]dioxol-4-ylmethyl)-6-bromo-2-methoxyquinoline (**AB-5**) (1.52 g, 76%). ^1^H NMR (CDCl_3_) δ 7.77 (d, J = 2.2 Hz, 1H), 7.69 (d, J = 8.9 Hz, 1H), 7.61 (dd, J = 8.9, 2.2 Hz, 1H), 7.53 (s, 1H) , 6.82-6.73 (m, 2H), 6.70 (dd, J = 7.4, 1.7 Hz, 1H), 5.94 (s, 2H), 4.09 (s, 3H), 3.98 (s, 2H). Found: [M+H]=373.1

**3-(Benzofuran-7-ylmethyl)-6-bromo-2-methoxyquinoline (AB-6)**

A solution of methyl benzofuran-7-carboxylate (**S12**) (3.59 g, 20.4 mmol) in Et_2_O (100 mL, dist. Na) at 0 °C was treated with LiAlH_4_ (1.54 g, 40.6 mmol) then stirred at r.t. for 3 h and quenched with ice. The mixture was partitioned between Et_2_O and sat. aq. sodium potassium tartrate and then filtered through Celite. The aqueous layer was extracted with Et_2_O and the organic phases were combined and dried. Column chromatography (0-5% EtOAc:DCM) gave benzofuran-7-ylmethanol (**S13**) (2.60 g, 86%). ^1^H NMR (CDCl_3_) δ 7.65 (d, J = 2.2 Hz, 1H), 7.56 (dd, J = 7.7, 1.2 Hz, 1H), 7.31 (dd, J = 7.3, 0.6 Hz, 1H), 7.23 (t, J = 7.5 Hz, 1H), 6.80 (d, J = 2.2 Hz, 1H), 5.02 (d, J = 6.2 Hz, 2H), 1.93 (t, J = 6.2 Hz, 1H).

A solution of **S13** (4.72 g, 31.8 mmol) in DCM (100 mL, anhydrous) at 0 °C was treated sequentially with triethylamine (8.9 mL, 63.9 mmol) then mesyl chloride (3.70 mL, 47.8 mmol), the mixture was stirred at 0 °C for 1 h then partitioned between DCM and water. The organic fraction was dried and evaporated and the residue was dissolved in acetone (200 mL), LiBr (27.6 g, 318 mmol) was added and the mixture was refluxed for 0.5 h and then evaporated. The residue was partitioned between DCM and water; the organic fraction was dried and evaporated. Column chromatography (DCM) gave 7-(bromomethyl)benzofuran (**S14**) (6.08 g, 90%). ^1^H NMR (CDCl_3_) δ 7.69 (d, J = 2.2 Hz, 1H), 7.57 (dd, J = 7.7, 1.2 Hz, 1H), 7.32 (dd, J = 7.4, 0.7 Hz, 1H), 7.22 (t, J = 7.6 Hz, 1H), 6.80 (d, J = 2.2 Hz, 1H), 4.81 (s, 2H).

A mixture of **(**6-bromo-2-methoxyquinolin-3-yl)boronic acid (8.00 g, 28.4 mmol), **S14** (5.99 g, 28.4 mmol) and Cs_2_CO_3_ (18.5 g, 56.7 mmol) in toluene (100 mL) and DMF (50 mL) was purged with nitrogen. Pd(PPh_3_)_4_ (0.66 g, 0.57 mmol) was added, the mixture was purged with nitrogen then heated to 80 °C under nitrogen for 3 h. The reaction was partitioned between EtOAc and water and the organic fraction was dried and evaporated. Column chromatography with 3:1 hexanes:DCM eluted impurities, then elution with 1:1 hexanes:DCM then DCM gave 3-(benzofuran-7-ylmethyl)-6-bromo-2-methoxyquinoline (**AB-6**) (6.95 g, 67%). ^1^H NMR (CDCl_3_) δ 7.71 (d, J = 2.2 Hz, 1H), 7.68 (d, J = 8.8 Hz, 1H), 7.58 -7.62 (m, 2H), 7.50-7.54 (m, 2H) , 7.20 (t, J = 7.4 Hz, 1H), 7.13 (dd, J = 7.4, 0.6 Hz, 1H), 6.79 (d, J = 2.2 Hz, 1H), 4.32 (s, 2H), 4.10 (s, 3H). Found: [M+H]=368.8

**6-Bromo-3-((5-isopropoxy-2-methoxypyridin-3-yl)methyl)-2-methoxyquinoline (AB-7)**

A mixture of 5-hydroxy-2-methoxynicotinaldehyde (**S15**) (Organic & Biomolecular Chemistry, 6(8), 1364-1376; 2008) (1.00 g, 6.53 mmol) and potassium carbonate (1.35 g, 9.80 mmol) in DMF (30 mL, anhydrous) was heated at 50 °C for 10 min. Isopropyl iodide (0.78 mL, 7.84 mmol) was then added and the mixture stirred at this temperature for 2 hours. The resultant solution was diluted with EtOAc and washed with brine three times. The organic layer was dried and evaporated to afford 5-isopropoxy-2-methoxynicotinaldehyde (**S16**) (0.90 g, 71%. ^1^H NMR (CDCl_3_) δ 10.34 (1H, s), 8.07 (1H, d, J = 3.2 Hz), 7.66 (1H, d, J = 3.2 Hz), 4.48 (1H, sp, J = 6.1 Hz), 4.03 (3H, s), 1.33 (6H, d, J = 4.8 Hz).

A mixture of **S16** (0.90 g, 4.61 mmol) and sodium borohydride (0.35 g, 9.22 mmol) in MeOH (15 mL, anhydrous) was stirred at r.t. for 1 hour. The solvent was then removed and the residue partitioned between EtOAc and water. The organic layer was dried and evaporated. Column chromatography with 9:1 hexanes/EtOAc afforded (5-isopropoxy-2-methoxypyridin-3-yl)methanol (**S17**)**,** (0.68 g, 75%. ^1^H NMR (CDCl_3_) δ 7.72 (1H, s), 7.25 (1H, d, J = 3 Hz), 4.61 (2H, s), 4.42 (1H, sp), 3.93 (3H, s), 2.92-2.19 (1H, br s), 1.32 (6H, d, J = 6.1 Hz).

To a solution of **S17** (0.68 g, 3.45 mmol) and triethylamine (0.72 mL, 5.18 mmol) in DCM (10 mL, anhydrous) at r.t. was added mesyl chloride (0.32 mL, 4.14 mmol) dropwise. After 15 min, the reaction was diluted with DCM (10 mL) and the organic layer washed with sat. NaHCO_3_, dried and evaporated. The residue was redissolved in acetone (20 mL, anhydrous), lithium bromide (excess) added, and the mixture heated at reflux for 30 min. The solution was then cooled and the solvent evaporated, and the residue partitioned between EtOAc and water. The aqueous layer was extracted twice with EtOAc and the organic layer was dried and evaporated to afford 3-(bromomethyl)-5-isopropoxy-2-methoxypyridine (**S18**) (0.70 g, 78%. ^1^H NMR (CDCl_3_) δ 7.76 (1H, dd, J = 2.6, 3.4 Hz), 7.25 (1H, dd, J = 2.4, 2.4 Hz), 4.5 (2H, d, J = 2.2 Hz), 4.42 (1H, sp, J = 2.6, 6.0 Hz), 3.96 (3H, d, J = 2.9 Hz), 1.32 (6H, dd, J = 3.0, 6.1 Hz).

A mixture of (6-bromo-2-methoxyquinolin-3-yl)boronic acid (0.80 g, 2.82 mmol), **S18** (0.70 g, 2.69 mmol) and cesium carbonate (1.75 g, 5.38 mmol) in toluene (10 mL, anhydrous) and DMF (5 mL, anhydrous) was purged with nitrogen. Pd(PPh_3_)_4_ (0.12 g, 0.11 mmol) was then added, and the mixture then heated to 80 °C under nitrogen for 4 hours. The reaction was partitioned between EtOAc and water and the organic fraction was dried and evaporated. Column chromatography (19:1 hexanes/EtOAc) gave 6-bromo-3-((5-isopropoxy-2-methoxypyridin-3-yl)methyl)-2-methoxyquinoline (**AB-7**) (0.57 g, 48%). ^1^H NMR (CDCl_3_) δ 7.77 (1H, d, J = 2.2 Hz), 7.71 (1H, d, J = 2.9 Hz), 7.69 (1H, d, J = 8.9 Hz), 7.62 (1H, dd, J = 2.2, 8.9 Hz), 7.56 (1H, s), 7.02 (1H, d, J = 2.9 Hz), 4.38 (1H, sp, J = 6.0 Hz), 4.08 (3H, s), 3.92 (2H, s), 3.90 (3H, s), 1.29 (6H, d, J = 6.1 Hz).

**6-Bromo-2-methoxy-3-((2,5,6-trimethoxypyridin-3-yl)methyl)quinolone (AB-8)**

To a mixture of 2,6-dimethoxypyridin-3-ol (**S19**) (8.05 g, 51.9 mmol) and imidazole (7.42 g, 108.99 mmol) in DMF (130 mL) at r.t. was added triisopropylsilyl chloride (13.33 mL, 62.23 mmol), and the resultant mixture stirred at r.t. for 2 hours. The solution was then partitioned between EtOAc and water, and the aqueous layer extracted three times. The combined organic layers were washed with brine three times, dried and evaporated. Column chromatography with 19:1 hexanes/EtOAc afforded the product 2,6-dimethoxy-3-((triisopropylsilyl)oxy)pyridine (**S20**) (15.59 g, 96%. ^1^H NMR (CDCl_3_) δ 7.07 (1H, d, J = 8.2 Hz), 6.13 (1H, d, J = 8.2 Hz), 3.92 (3H, s), 3.86 (3H, s), 1.27-1.18 (3H, m), 1.08 (18H, d, J = 7.1 Hz). Found: [M+H]=312.8.

To a solution of **S20** (8.00 g, 25.69 mmol) and *N*,*N*-diisopropylamine (0.18 mL, 1.28 mmol) in THF (100 mL, dist. Na) at -40 °C under nitrogen was added *n*-BuLi (15.41 mL, 30.83 mmol) dropwise. The resultant solution was stirred at -40 °C for 5 min, and then warmed to 0 °C and stirred at this temperature for a further 3 hours. The solution was then again cooled to -40 °C, and formylpiperidine (4.28 mL, 38.54 mmol) was added dropwise, and the mixture stirred at r.t. for another 1 hour. Acetic acid (8 mL) was added and the solvent was removed in vacuo. The resultant mixture was partitioned between EtOAc and water, and the organic fraction dried and evaporated. Column chromatography with 49:1 hexanes/EtOAc afforded the product 2,6-dimethoxy-5-((triisopropylsilyl)oxy)nicotinaldehyde (**S21**) (7.55 g, 87%. ^1^H NMR (CDCl_3_) δ 10.17 (1H, s), 7.51 (1H, s), 4.02 (3H, s), 4.01 (3H, s), 1.30-1.19 (3H, m), 1.08 (18H, d, J = 7.3 Hz). Found: [M-CHO]^+^= 312.8.

Tetrabutylammonium fluoride in THF (1N, 33.36 mL, 33.36 mmol) was added to a solution of **S21** (7.55 g, 22.24 mmol) in THF (35 mL, dist. Na) at 0°C. The reaction was then warmed to r.t. and stirred for 4 hours. The solvent was removed and the residue partitioned between EtOAc and water. The aqueous layer was extracted with EtOAc three times, and the organic layer dried and evaporated. Column chromatography with DCM followed by 3:1 DCM/EtOAc afforded the product 5-hydroxy-2,6-dimethoxynicotinaldehyde (**S22**) (3.15 g, 77%. ^1^H NMR (CDCl_3_) δ 10.20 (1H, s), 7.59 (1H, s), 5.15-4.80 (1H, br s), 4.10 (3H, s), 4.01 (3H, s).

A mixture of **S22** (3.15 g, 17.20 mmol) and potassium carbonate (3.57 g, 25.80 mmol) in DMF (80 mL, anhydrous) was heated at 50 °C for 10 min. Methyl iodide (1.29 mL, 20.64 mmol) was then added and the mixture stirred at this temperature for 2 hours. The resultant solution was diluted with EtOAc and washed with brine three times. The organic layer was dried and evaporated to afford the product 2,5,6-trimethoxynicotinaldehyde (**S23**) (3.39 g, 100%. ^1^H NMR (CDCl_3_) δ 10.21 (1H, s), 7.53 (1H, s), 4.10 (3H, s), 4.02 (3H, s), 3.87 (3H, s).

A solution of *N*,*N*,*N*,*N*-tetramethylpiperidine (3.59 mL, 21.03 mmol) in THF (40 mL, dist. Na) was cooled to -40 °C, *n*-BuLi (10.52 mL, 21.03 mmol) was added and the solution was stirred at -40 °C for 15 min, then cooled to -78 °C. A solution of 6-bromo-2-methoxyquinoline (17.53 mmol) in THF (40 mL, dist. Na) was added dropwise, the orange solution was stirred at -78 °C for 1.5 hours, then a solution of **S23** (3.42 g, 17.53 mmol) in THF (40 mL, dist. Na) was added. The mixture was stirred at -78 °C for 2 hours, then acetic acid (2.5 mL) was added and the solution was allowed to warm to r.t.. The solvent was removed and the residue partitioned between EtOAc and water, and the organic fraction was dried and evaporated. Column chromatography with 9:1 hexanes/EtOAc followed by 4:1 hexanes/EtOAc gave the product (6-bromo-2-methoxyquinolin-3-yl)(2,5,6-trimethoxypyridin-3-yl)methanol (**S24**) as a white solid. Yield = 5.50 g, 72%. ^1^H NMR (CDCl_3_) δ 7.85 (1H, d, J = 2.1 Hz), 7.79 (1H, s), 7.70 (1H, d, J = 8.9 Hz), 7.65 (1H, dd, J = 2.1, 8.9 Hz), 7.15 (1H, s), 6.14 (1H, d, J = 5.2 Hz), 4.08 (3H, s), 4.02 (3H, s), 3.90 (3H, s), 3.78 (3H, s), 3.48 (1H, d, J = 5.4 Hz). Found: [M+H]=436.1

Trifluoroacetic acid (11.30 mL, 148.2 mmol) and triethylsilane (17.76 mL, 111.2 mmol) were added sequentially to a solution of **S24** (5.35 g, 12.35 mmol) in DCM (125 mL) and the solution was stirred for 1 hour at r.t., then ice water was added. The solution was partitioned between sat. aq. NaHCO_3_ and DCM and the aqueous fraction was extracted with DCM. The organic fractions were combined, dried and evaporated. Column chromatography with 9:1 hexanes/EtOAc gave 6-bromo-2-methoxy-3-((2,5,6-trimethoxypyridin-3-yl)methyl)quinolone (**AB-8**) as a white solid (4.45 g, 86%. ^1^H NMR (CDCl_3_) δ 7.76 (1H, d, J = 2.2 Hz), 7.68 (1H, d, J = 8.9 Hz), 7.60 (1H, dd, J = 2.2, 8.4 Hz), 7.49 (1H, s), 7.05 (1H, s), 4.09 (3H, s), 4.01 (3H, s), 3.89 (5H, s), 3.79 (3H, s). Found: [M+H]=419.0

**5-((6-bromo-2-methoxyquinolin-3-yl)methyl)-N,N-diethylpyridin-2-amine (AB-9)**

To a solution of freshly distilled N,N,N,N,-tetramethylpiperidine (1.60 mL, 9.56 mmol) in freshly distilled THF (13 mL) was added at -30 °C under nitrogen , *n*-BuLi (4.40 mL, 8.76 mmol) dropwise. The mixture was maintained at about -30 ^o^C for 15 minutes, then cooled to -78 °C. A solution of 6-bromo-2-methoxyquinoline (**S25**) (1.90 g, 7.96 mmol) in dry THF (15 mL) was added dropwise at -78 °C. The resultant organic mixture was stirred at the same temperature for 75 minutes. A solution of 6-(diethylamino)nicotinaldehyde (**S26**) (Ohgiya, Tadaaki et. al. PCT Int. Appl., 2013137371, 19Sep 2013) (1.42 g, 7.96 mmol) in dry THF (6 mL) was added dropwise at -78 °C, the reaction mixture remained orange brown, stirred at -78 °C for 2.5 hours. The mixture was quenched with acetic acid (0.68 mL) at -65 °C. Water was added, the aqueous mixture was extracted with ethyl acetate (2x), and the combined extract was washed with brine, dried (MgSO_4_) and concentrated in vacuo to give the crude product as a yellow solid. Flash chromatography of the crude product using 10-100% ethyl acetate in hexane as eluent afforded product (6-bromo-2-methoxyquinolin-3-yl)(6-(diethylamino)pyridin-3-yl)methanol **S27** as an off-white solid. Yield = 1.90 g, 57%). ^1^H NMR (CDCl_3_) δ 8.15 (d, J = 2.4 Hz, 1H), 8.01 (s, 1H), 7.88 (d, J = 2.1 Hz, 1H), 7.70-7.64 (m, 2H), 7.39 (dd, J = 8.9, 2.5 Hz, 1H), 6.43 (d, J = 8.9 Hz, 1H), 5.94 (d, J = 3.7 Hz, 1H), 4.04 (s, 3H), 3.53-3.47 (m, 4H), 2.65 (d, J = 3.9 Hz, 1H), 1.17 (t, J = 7.0 Hz, 6H).

To a sparingly soluble solution of **S27** (1.90 g, 4.57 mmol) in freshly distilled THF (19 mL) was added at 2 °C under nitrogen sodium borohydride (0.86 g, 23.0 mmol) in 3 portions over 10 minutes. The mixture was stirred at 2-4 °C for 1 hour. The mixture was cooled to 2 °C again, aluminium chloride (1.83 g, 13.7 mmol) was added in 4 batches over 15 minutes. The mixture was stirred at 2 °C for 10 minutes, then refluxed for 2 hours.

The mixture was then quenched with water cautiously at 2 °C, until gas evolution ceased.

The white slurry was filtered through celite. The milky white filtrate was diluted in water, and the organic phase was collected. The aqueous phase was extracted with ethyl acetate (3x). The organic extract was washed with brine, dried (MgSO_4_) and concentrated in vacuo to furnish the crude product as a brownish residue. Flash chromatography of the crude product using 10% ethyl acetate in hexane as eluent afforded product 5-((6-bromo-2-methoxyquinolin-3-yl)methyl)-N,N-diethylpyridin-2-amine (**AB-9**) as a white solid. Yield = 1.39 g, 76%. ^1^H NMR (CDCl_3_) δ 8.06 (d, J = 2.1 Hz, 1H), 7.76 (d, J = 2.2 Hz, 1H), 7.67 (d, J = 8.8 Hz, 1H), 7.60 (dd, J = 8.8, 2.2 Hz, 1H), 7.52 (s, 1H), 7.28 (dd, J = 8.7, 2.4 Hz, 1H), 6.42 (dd, J = 8.7, 0.3 Hz, 1H), 4.09 (s, 3H), 3.85 (s, 2H), 3.50 (q, J = 7.0 Hz, 4H), 1.18 (t, J = 7.0 Hz, 6H).

**6-Bromo-3-((2,5-dimethoxypyridin-4-yl)methyl)-2-methoxyquinoline (AB-10)**

To a solution of 6-methoxypyridin-3-ol (**S28**) (3.20 g, 25.57 mmol) in E (50 mL, anhydrous) at 0 °C was added sodium hydride (60% in mineral dispersion, 1.23 g, 30.69 mmol) in portions. The mixture was warmed to r.t. and stirred for 1 h. Chloromethyl ethyl ether (2.73 ml, 29.41 mmol) was then added, and the resultant mixture stirred at r.t. for a further 2 h. The reaction was diluted with water and extracted with EtOAc (x3). The organic layer was washed with brine (x3), dried and evaporated. Column chromatography with 9:1 X4:EtOAc afforded 5-(ethoxymethoxy)-2-methoxypyridine (**S29**). Yield = 4.09 g, 87%. ^1^H NMR (CDCl_3_) δ 7.95 (d, J = 3.0 Hz, 1H), 7.34-7.31 (m, 1H), 6.67 (d, J = 9.0 Hz, 1H), 5.13 (s, 2H), 3.88 (s, 3H), 3.72 (q, J = 7.1 Hz, 2H), 1.22 (t, J = 7.0 Hz, 3H). Found: [M+H]=184.4.

To a solution of 5-(ethoxymethoxy)-2-methoxypyridine (**S29**) (6.20 g, 33.84 mmol) and diisopropylamine (0.24 ml, 1.69 mmol) in THF (100 mL, dist. Na) at -40 °C under nitrogen was added *n*-BuLi (25.4 mL, 50.76 mmol) dropwise. The resultant solution was stirred at -40 °C for 5 min, and then warmed to 0 °C and stirred at this temperature for a further 3 h. The solution was then again cooled to -40 °C, and *n*-formylpiperidine (6.76 ml, 60.91 mmol) was added dropwise, and the mixture stirred at r.t. for another 1 h. Acetic acid (15 mL) was added and the solvent was removed in vacuo. The resultant mixture was partitioned between EtOAc and water, and the organic fraction dried and evaporated. Column chromatography with 9:1 X4:EtOAc afforded 5-(ethoxymethoxy)-2-methoxyisonicotinaldehyde (**S30**). Yield = 4.00 g, 56%. ^1^H NMR (CDCl_3_) δ 10.43 (s, 1H), 8.27 (s, 1H), 7.07 (d, J = 0.4 Hz, 1H), 5.30 (s, 2H), 3.92 (s, 3H), 3.78 (q, J = 7.1 Hz, 2H), 1.26 (t, J = 7.1 Hz, 3H).

A solution of 5-(ethoxymethoxy)-2-methoxyisonicotinaldehyde (**S30**) (4.00 g, 18.85 mmol) and 3M HCl (60 mL) in THF (40 mL, dist. Na) was heated at 40 °C for 3 h. The solution was then cooled, diluted with water, and the pH adjusted to 7 using potassium carbonate. The aqueous layer was then extracted with EtOAc three times, and the organic layer dried and evaporated. Column chromatography with 9:1 X4:EtOAc afforded 5-hydroxy-2-methoxyisonicotinaldehyde (**S31**). Yield = 2.50 g, 87%. ^1^H NMR (CDCl_3_) δ 9.97 (d, J = 0.7 Hz, 1H), 9.46 (s, 1H), 8.08 (s, 1H), 6.93 (d, J = 0.6 Hz, 1H), 3.94 (s, 3H).

A mixture of 5-hydroxy-2-methoxyisonicotinaldehyde (**S31**) (2.50 g, 16.33 mmol) and potassium carbonate (3.39 g, 24.45 mmol) in DMF (80 mL, anhydrous) was heated at 50 °C for 10 min. Methyl iodide (1.22 ml, 19.59 mmol) was then added and the mixture stirred at this temperature for 2 h. The resultant solution was diluted with EtOAc and washed with brine three times. The organic layer was dried and evaporated to afford 2,5-dimethoxyisonicotinaldehyde (**S32**). Yield = 2.25 g, 82%. ^1^H NMR (CDCl_3_) δ 10.4 (s, 1H), 8.01 (s, 1H), 7.07 (s, 1H), 3.97 (s, 3H), 3.91 (s, 3H). Found [M+MeOH]= 200.4

A mixture of 2,5-dimethoxyisonicotinaldehyde (**S32**) (2.25 g, 13.46 mmol) and sodium borohydride (1.02 g, 26.92 mmol) in MeOH (50mL, anhydrous) was stirred at r.t. for 1 h. The solvent was then removed and the residue partitioned between EtOAc and water. The organic layer was dried and evaporated to afford (2,5-dimethoxypyridin-4-yl)methanol (**S33**). Yield = 2.17 g, 95%. ^1^H NMR (CDCl_3_) δ 7.70 (s, 1H), 6.77 (s, 1H), 4.66 (d, J = 6.0 Hz, 2H), 3.89 (s, 3H), 3.87 (s, 3H), 2.23 (t, J = 6.4 Hz, 1H).

To a solution of (2,5-dimethoxypyridin-4-yl)methanol (**S33**) (2.05 g, 12.12 mmol) and triehylamine (2.53 ml, 18.18 mmol) in DCM (35 mL, anhydrous) at r.t. was added methanesulfonyl chloride (1.13 ml, 14.50 mmol) dropwise. After 15 min, the reaction was diluted with DCM (20 mL) and the organic layer washed with sat. sodium hydrogen carbonate, dried and evaporated. The residue was redissolved in acetone (70 mL, anhydrous), Lithium bromide (10 g, excess) added, and the mixture heated at reflux for 30 min. The solution was then cooled and the solvent evaporated, and the residue partitioned between EtOAc and water. The aqueous layer was extracted twice with EtOAC and the organic layer was dried and evaporated to afford 4-(bromomethyl)-2,5-dimethoxypyridine (**S34**). Yield = 2.54 g, 90%. ^1^H NMR (CDCl_3_) δ 7.75 (s, 1H), 6.75 (s, 1H), 4.40 (s, 2H), 3.91 (s, 3H), 3.88 (s, 3H).

A mixture of (6-bromo-2-methoxyquinolin-3-yl)boronic acid (2.80 g, 9.95 mmol), 4-(bromomethyl)-2,5-dimethoxypyridine (**S34**) (2.54 g, 10.94 mmol) and cesium carbonate (6.50 g, 19.9 mmol) in toluene (50 mL, anhydrous) and DMF (25 mL, anhydrous) was purged with nitrogen. Pd(PPh_3_)_4_ (0.046 g, 0.40 mmol) was then added, the mixture purged with nitrogen then heated to 80 °C under nitrogen for 4 h. The reaction was partitioned between EtOAc and water and the organic fraction was dried and evaporated. Column chromatography (19:1 x4:EtOAc) gave 6-bromo-3-((2,5-dimethoxypyridin-4-yl)methyl)-2-methoxyquinoline (**AB-10**). Yield = 2.20 g, 51%. ^1^H NMR (CDCl_3_) δ 7.77 (d, J = 2.2 Hz, 1H), 7.75 (s, 1H), 7.69 (d, J = 8.9 Hz, 1H), 7.62 (dd, J = 8.9, 2.2 Hz, 1H), 7.53 (s, 1H), 6.48 (s, 1H), 4.07 (s, 3H), 3.97 (s, 2H), 3.86 (s, 3H), 3.83 (s, 3H). Found: [M+H]=389.7.

**6-bromo-3-((2-isopropoxy-5-methoxypyridin-4-yl)methyl)-2-methoxyquinoline (AB-11)**

Sodium metal (2.04 g, 85.0 mmol) was added to a solution of isopropanol (150 mL) and the mixture stirred at reflux for 3 h. The solution was then cooled to room temperature and 5-bromo-2-fluoropyridine (**S35**) (10.0 g, 56.67 mmol) was added, and the reaction heated at 80 °C for 0.5 h. The solvent was then removed and the residue partitioned between EtOAc and water, and the organic extract was dried and evaporated to afford 5-bromo-2-isopropoxypyridine (**S36**). Yield = 10.9 g, 89%. ^1^H NMR (CDCl_3_) δ 8.16 (d, J = 2.5 Hz, 1H), 7.60 (dd, J = 8.8, 2.6 Hz, 1H), 6.58 (d, J = 8.8 Hz, 1H), 5.23 (sep, J = 6.2 Hz, 1H), 1.34 (s, 3H), 1.32 (s, 3H). Found: [M+H]=215.5.

To a solution of 5-bromo-2-isopropoxypyridine (**S36**) (9.85 g, 45.6 mmol) in THF (200 mL, dist. Na) at -78 °C was added *n*-BuLi (2.0 M in cyclohexane, 36.48 ml, 72.96 mmol) dropwise over 10 min. The reaction was stirred for 20 min and then trimethyl borate (13.24 ml, 72.96 mmol) was added dropwise over 5 min. The resulting mixture was stirred for 2 h at -78 °C, then peracetic acid solution (32 wt. % in dilute acetic acid, 25.69 ml, 72.96 mmol) was added. After 10 min at -78 °C, the reaction was warmed to 0 °C and stirred for 1 h. The reaction was then quenched with aqueous sodium bisulfite and stirred for 15 min at 0 °C. The solvent was then concentrated, sodium bicarbonate added and the aqueous layer extracted with EtOAc. The combined organic extracts were dried and evaporated. Column chromatography with 4:1 x4:EtOAc afforded 6-isopropoxypyridin-3-ol (**S37**). Yield = 5.40 g, 65%. ^1^H NMR (CDCl_3_) δ 7.76 (dd, J = 3.1, 0.4 Hz, 1H), 7.17 (dd, 8.9, 3.1 Hz, 1H), 6.61 (d, J = 8.9 Hz, 1H), 6.16 (br s, 1H), 5.09 (sep, J = 6.1 Hz, 1H), 1.34 (s, 3H), 1.32 (s, 3H).

To a solution of 6-isopropoxypyridin-3-ol (**S37**) (6.00 g, 32.97 mmol) in DMF (80 mL, anhydrous) at 0 °C was added sodium hydride (60% in mineral dispersion, 1.57 g, 39.54 mmol) in portions. The mixture was warmed to r.t. and stirred for 1 h. Chloromethyl ethyl ether (3.50 ml, 37.71 mmol) was then added, and the resultant mixture stirred at r.t. for a further 2 h. The reaction was diluted with brine (100 mL) and extracted with EtOAc (x3) times. The organic layer was washed with brine (x3), dried and evaporated. Column chromatography with 19:1 x4:EtOAc afforded 5-(ethoxymethoxy)-2-isopropoxypyridine (**S38**). Yield = 6.50 g, 82%. ^1^H NMR (CDCl_3_) δ 7.94 (d, J = 3.0 Hz, 1H), 7.33-7.30 (m, 1H), 6.61 (d, J = 8.9 Hz, 1H), 5.20 (sep, J = 6.2 Hz, 1H), 5.14 (s, 2H), 3.73 (q, J = 7.0 Hz, 2H), 1.33 (s, 3H), 1.32 (s, 3H), 1.23 (t, J = 7.1 Hz, 3H). Found: [M+H]=211.5.

To a solution of 5-(ethoxymethoxy)-2-isopropoxypyridine (**S38**) (6.50 g, 26.97 mmol) and diisopropykamine (0.19 ml, 1.35 mmol) in THF (80 mL, dist. Na) at -40 °C under nitrogen was added *n*-BuLi (2.0 M in cyclohexane, 16.18 ml, 32.36 mmol) dropwise. The resultant solution was stirred at -40 °C for 5 min, and then warmed to 0 °C and stirred at this temperature for a further 3 h. The solution was then again cooled to -40 °C, and *n*-formylpiperidine (4.49 ml, 40.46 mmol) was added dropwise, and the mixture stirred at r.t. for another 1 h. Acetic acid (12 mL) was added and the solvent was removed in vacuo. The resultant mixture was partitioned between EtOAc and water, and the organic fraction dried and evaporated. Column chromatography with 19:1 x4:EtOAc afforded 5-(ethoxymethoxy)-2-isopropoxyisonicotinaldehyde (**S39**). Yield = 5.51 g, 76%. ^1^H NMR (CDCl_3_) δ 10.41 (s, 1H), 8.25 (s, 1H), 7.01 (d, J = 0.5 Hz, 1H), 5.29 (s, 2H), 5.23-5.16 (m, 1H), 3.79 (q, J = 7.1 Hz, 2H), 1.34 (s, 3H), 1.32 (s, 3H), 1.26 (t, J = 7.1 Hz, 3H).

A solution of 5-(ethoxymethoxy)-2-isopropoxyisonicotinaldehyde (**S39**) (5.51 g, 20.48 mmol) and 3M HCl (65 mL) in THF (45 mL, dist. Na) was heated at 40 °C for 1.5 h. The solution was then cooled, diluted with water, and the pH adjusted to 7 using sodium hydrogen carbonate. The aqueous layer was then extracted with EtOAc (x3), and the organic layer dried and evaporated to afford 5-hydroxy-2-isopropoxyisonicotinaldehyde (**S40**). Yield = 3.71 g, 100%. ^1^H NMR (CDCl_3_) δ 9.96 (s, 1H), 8.06 (s, 1H), 6.87 (s, 1H), 5.21 (sep, J = 6.2 Hz, 1H), 1.34 (s, 3H), 1.32 (s, 3H).

A mixture of 5-hydroxy-2-isopropoxyisonicotinaldehyde (**S40**) (3.80 g, 20.99 mmol) and potassium carbonate (4.35 g, 31.49 mmol) in DMF (100 mL, anhydrous) was heated at 50 °C for 10 min. Methyl iodide (1.57 ml, 25.19 mmol) was then added and the mixture stirred at this temperature for 2 h. The resultant solution was diluted with EtOAc and washed with brine (x3). The organic layer was dried and evaporated. Column chromatography with 19:1 x4:EtOAc afforded 2-isopropoxy-5-methoxyisonicotinaldehyde (**S41**). Yield = 2.60 g, 63%. ^1^H NMR (CDCl_3_) δ 10.42 (s, 1H), 7.99 (s, 1H), 7.02 (d, J = 0.5 Hz, 1H), 5.17 (sep, J = 6.2 Hz, 1H), 1.34 (s, 3H), 1.32 (s, 3H).

A mixture of 2-isopropoxy-5-methoxyisonicotinaldehyde (**S41**) (2.60 g, 13.32 mmol) and sodium borohydride (1.01 g, 26.65 mmol) in methanol (50 mL, anhydrous) was stirred at r.t. for 1 h. The solvent was then removed and the residue partitioned between EtOAc and water. The organic layer was dried and evaporated to afford (2-isopropoxy-5-methoxypyridin-4-yl)methanol (**S42**). Yield = 2.59 g, 99%. ^1^H NMR (CDCl_3_) δ 7.68 (s, 1H), 6.71 (s, 1H), 5.17 (sep, J = 6.2 Hz, 1H), 4.64 (d, J = 5.7 Hz, 2H), 3.85 (s, 3H), 2.40 (t, J = 6.1 Hz, 1H), 1.33 (s, 3H), 1.31 (s, 3H).

To a solution of (2-isopropoxy-5-methoxypyridin-4-yl)methanol (**S42**) (2.59g, 13.14 mmol) and triethylamine (2.74 ml, 19.71 mmol) in DCM (40 mL, anhydrous) at r.t. was added methanesulfonyl chloride (1.23 ml, 15.77 mmol) dropwise. After 15 min, the reaction was diluted with DCM (40 mL) and the organic layer washed with sat. sodium hydrogen carbonate, dried and evaporated. The residue was redissolved in acetone (80 mL, anhydrous), Lithium bromide (10 g, excess) added, and the mixture heated at reflux for 30 min. The solution was then cooled and the solvent evaporated, and the residue partitioned between EtOAc and water. The aqueous layer was extracted with EtOAC (x2) and the organic layer was dried and evaporated to afford 4-(bromomethyl)-2-isopropoxy-5-methoxypyridine (**S43**). Yield = 3.42 g, 100%. ^1^H NMR (CDCl_3_) δ 7.73 (s, 1H), 6.69 (s, 1H), 5.17 (sep, J = 6.2 Hz, 1H), 4.38 (s, 2H), 3.90 (s, 3H),1.33 (s, 3H), 1.31 (s, 3H).

A mixture of (6-bromo-2-methoxyquinolin-3-yl)boronic acid (3.92 g, 13.92 mmmol), 4-(bromomethyl)-2-isopropoxy-5-methoxypyridine (**S43**) (3.45 g, 13.26 mmol) and cesium carbonate (8.64 g, 26.52 mmol) in toluene (50 mL, anhydrous) and DMF (25 mL, anhydrous) was purged with nitrogen. Pd(PPh_3_)_4_ (0.61 g, 0.53 mmol) was then added, the mixture purged with nitrogen then heated to 80 °C under nitrogen for 4 h. The reaction was partitioned between EtOAc and water and the organic fraction was dried and evaporated. Column chromatography (19:1 x4:EtOAc) gave 6-bromo-3-((2-isopropoxy-5-methoxypyridin-4-yl)methyl)-2-methoxyquinoline (**AB-11**). Yield = 2.85 g, 49%. ^1^H NMR (CDCl_3_) δ 7.77 (d, J = 2.2 Hz, 1H), 7.72 (s, 1H), 7.70 (d, J = 8.9 Hz, 1H), 7.62 (dd, J = 8.9, 2.2 Hz, 1H), 7.54 (s, 1H), 6.38 (s, 1H), 5.17 (sep, J = 6.2 Hz, 1H), 4.07 (s, 3H), 3.96 (s, 2H), 3.83 (s, 3H), 1.31 (s, 3H), 1.29 (s, 3H).

**4-((6-Bromo-2-methoxyquinolin-3-yl)methyl)-6-methoxy-N,N-dimethylpyridin-2-amine (B-12)**

To a solution of (2-(dimethylamino)-6-methoxypyridin-4-yl)methanol (**S44**(3.30 g, 18.1 mmol) in anhydrous DCM (54 mL) was added at 2 °C under nitrogen triethylamine (3.8 mL, 27.2 mmol) dropwise, followed by mesyl chloride (1.7 mL, 21.7 mmol). The mixture was stirred from 2 °C for 10 min, then at r.t. for 2 hours. The mixture was quenched with sat. aq. NaHCO_3_ solution. The aqueous mixture was extracted with DCM (2x) and the combined extract was washed with brine, dried (MgSO_4_) and concentrated to afford the crude product as a beige solid. The crude intermediate was dissolved in acetone (60 mL). Lithium bromide (1.53 g, 36.2 mmol) was added and the suspension was refluxed for 2 hours. Flash chromatography using a mixture of 3-5% Et_2_O in hexanes as eluent gave 4-(bromomethyl)-6-methoxy-N,N-dimethylpyridin-2-amine (**S45**) as a mobile yellow oil, 3.78 g, 85%. ^1^H NMR (CDCl_3_) δ 6.02 (1H, d, J = 0.8 Hz), 6.00 (1H, d, J = 0.4 Hz), 4.27 (2H, s), 3.88 (3H, s), 3.06 (6H, s).

A mixture of (6-bromo-2-methoxyquinolin-3-yl)boronic acid (4.34 g, 15.4 mmol), **S45** (3.77 g, 115.4 mmol) and cesium carbonate (10.03 g, 30.8 mmol) in a mixture of toluene (40 mL) and DMF (20 mL) was purged with nitrogen. Pd(PPh_3_)_4_ (0.890 g, 0.774 mmol) was added and the mixture was purged again with nitrogen and heated at 85 °C under nitrogen for 3.5 hours. The mixture was partitioned between water and EtOAc and the mixture was extracted with EtOAc (2x). The extract was washed with water (2x), brine, dried (MgSO_4_) and concentrated to afford the crude product as a brown solid which was chromatographed using 3-10% Et_2_O in hexanes as eluent to yield the product 4-((6-bromo-2-methoxyquinolin-3-yl)methyl)-6-methoxy-N,N-dimethylpyridin-2-amine (**AB-12**) as a light yellow solid, 3.69 g, 60%. ^1^H NMR (CDCl_3_) δ 7.76 (1H, d, J = 2 Hz), 7.68 (1H, d, J = 8.8 Hz), 7.61 (1H, dd, J = 2, 8.8 Hz), 7.55 (1H, s), 5.92 (1H, s), 5.86 (1H, s), 4.07 (3H, s), 3.87 (5H, s), 3.04 (6H, s). Found: [M+H]=402.0

**4-((6-Bromo-2-methoxyquinolin-3-yl)methyl)-6-ethoxy-N,N-dimethylpyridin-2-amine (AB-13)**

To a glass tube was charged ethyl 2-chloro-6-ethoxyisonicotinate (**S46**; (WO 2010/080864) (1.00 g, 4.37 mmol), diphenylphosphino-1,1'-binaphthol (0.44 g, 0.70 mmol) and cesium carbonate (1.99 g, 6.12 mmol) under continuous nitrogen flow. Anhydrous toluene (24 mL) was added. The mixture was purged with nitrogen 5 min. Palladium acetate (0.079 g, 0.35 mmol) was added, the mixture was purged again with nitrogen. Dimethylamine in THF (2N, 2.6 mL, 5.246 mmol) was added and the mixture was sealed in the tube and heated at 80 °C overnight. The mixture was filtered through Celite, washing with EtOAc and the filtrate was concentrated in vacuo to yield the crude product as a dark red liquid. Flash chromatography using 2-4% Et_2_O in hexanes gave ethyl 2-(dimethylamino)-6-ethoxyisonicotinate (**S47**) as a light yellow oil. Yield = 0.85 g, 82%. ^1^H NMR (CDCl_3_) δ 6.60 (1H, d, J = 0.8 Hz), 6.51 (1H, d, J = 0.8 Hz), 4.35 (2H, q, J = 7.0 Hz), 4.33 (2H, q, J = 7.1 Hz), 3.09 (6H, s), 1.38 (3H, t, J = 7.2 Hz), 1.37 (3H, t, J = 7.2 Hz).

To a solution of **S47** (4.40 g, 18.50 mmol) in freshly distilled THF (90 mL) was added at -78 °C under nitrogen lithium aluminium hydride (0.91 g, 24.0 mmol) in 3 batches. The mixture was stirred at -78 °C for 15 min then at r.t. for 1 hour. The mixture was quenched cautiously with water at 2 °C until gas evolution ceased. 1M NaOH (32 mL) was added and the mixture was stirred for 1 hour, then the aqueous mixture was diluted with water and extracted with EtOAc (3x). The combined organic extract was washed with brine, dried (Na_2_SO_4_) and concentrated to give the product, which was purified via flash chromatography eluting with mixtures of 6:1 then 4:1 hexanes/EtOAc to give the alcohol intermediate as a light yellow oil. Yield = 3.27 g, 90%. The material was used directly in the next step without further characterisation.

To a solution of the alcohol intermediate (3.27 g, 16.7 mmol) in anhydrous DCM (50 mL) was added at 2 °C under nitrogen triethylamine (3.5 mL, 25.0 mmol) dropwise, followed by mesyl chloride (1.6 mL, 20.0 mmol). The mixture was stirred from 2 °C for 10 min, then at r.t. for 0.5 hour. The mixture was quenched with sat. aq. NaHCO_3_ solution. The aqueous mixture was extracted with DCM (3x) and the combined extract was washed with brine, dried and concentrated to afford the crude product as a brown oil. This was diluted in acetone (60 mL), lithium bromide (1.42 g) was added. And the suspension was refluxed for 2 hours. Flash chromatography using a mixture of 2-3% Et_2_O in hexanes as eluent gave 4-(bromomethyl)-6-ethoxy-N,N-dimethylpyridin-2-amine (**S48**) as a mobile yellow oil. Yield = 3.84 g, 89%. ^1^H NMR (CDCl_3_) δ 6.011 (1H, s), 5.99 (1H, s), 4.30 (2H, q, J = 6.8 Hz), 4.27 (2H, s), 3.05 (6H, s), 1.37 (3H, t, J = 7.2 Hz).

A mixture of (6-bromo-2-methoxyquinolin-3-yl)boronic acid (4.13 g, 14.7 mmol), **S48** (3.80 g, 14.7 mmol) and cesium carbonate (9.58 g, 29.4 mmol) in a mixture of toluene (40 mL) and DMF (20 mL) was purged with nitrogen. Pd(PPh_3_)_4_ (0.68 g) was added. The mixture was purged again with nitrogen and heated at 85 °C under nitrogen for 3 hours. The mixture was partitioned between water and EtOAc and the aqueous mixture was extracted with EtOAc (2x).The extract was washed with water (2x), brine, dried and concentrated to afford the crude product as a brown oil. This was chromatographed using 3-10% Et_2_O in hexanes as eluent to yield 4-((6-bromo-2-methoxyquinolin-3-yl)methyl)-6-ethoxy-N,N-dimethylpyridin-2-amine (**AB-13**) as a yellow solid (3.83 g), which was triturated in diethyl ether to afford the clean product as a pale yellow solid. Yield = 3.51 g, 57%. ^1^H NMR (CDCl_3_) δ 7.76 (1H, d, J = 2.2 Hz), 7.68 (1H, d, J = 8.8 Hz), 7.61 (1H, dd, J = 2.2, 8.8 Hz), 7.55 (1H, s), 5.91 (1H, s), 5.84 (1H, s), 4.30 (2H, q, J = 7.1 Hz), 4.07 (3H, s), 3.87 (2H, s), 3.03 (6H, s), 1.36 (3H, t, J = 7.1 Hz).

**6-Bromo-2-methoxy-3-((2,3,5-trimethoxypyridin-4-yl)methyl)quinolone (AB-14)**

To a solution of 2,6-dimethoxypyridine (10 g, 71.84 mmol) and *N*,*N*-diisopropylamine (0.50 mL, 3.59 mmol) in THF (200 mL, dist. Na) at -40°C under nitrogen was added *n*-BuLi (43.10 mL, 86.21 mmol) dropwise. The resultant solution was stirred at -40°C for 5 min, and then warmed to 0 °C and stirred at this temperature for a further 3 hours. The solution was then again cooled to -40 °C, and triisopropylborate (24.87 mL, 107.76 mmol) was added dropwise, and the mixture stirred at r.t. for another 1 hour. Water (50 mL) was added and the solvent was removed in vacuo. To the residue was added 1M NaOH (100 mL) and the aqueous layer washed with EtOAc (2 x 100 mL). The aqueous layer was then acidified to pH 3 and a solid precipitated. This solid was filtered and dried to afford 2,6-dimethoxypyridin-3-yl)boronic acid (**S49**), 8.10 g, 61%. ^1^H NMR (DMSO-d_6_) δ 7.87 (1H, d, J = 7.9 Hz), 6.36 (1H, d, J = 7.9 Hz), 3.90 (3H, s), 3.87 (3H, s).

To a solution of **S49** (8.00 g, 43.49 mmol) in THF (150 mL, dist. Na) at 0 °C was added dropwise 32% peracetic acid in acetic acid (21.53 mL, 86.98 mmol) over 10 min. The resultant solution was stirred at r.t. for 2 h. A 10% solution of sodium sulfite (75 mL) was then added and the mixture stirred at r.t. for 0.5 hour. The solvent was evaporated and the residue partitioned between EtOAc and water. The aqueous layer was extracted twice and the organic layer dried and evaporated. Column chromatography with 9:1 hexanes/EtOAc afforded 2,6-dimethoxypyridin-3-ol (**S50**), 6.05 g, 90%. ^1^H NMR (CDCl_3_) δ 7.12 (1H, d, J = 8.3 Hz), 6.21 (1H, d, J = 8.2 Hz), 4.90 (1H, s), 7.00 (3H, s), 3.86 (3H, s). Found: [M+H]=156.7.

To a solution of **S50** (6.45 g, 40.97 mmol) in DMF (70 mL, anhydrous) at 0 °C was added 60% sodium hydride in mineral oil (41.97 g, 9.16 mmol) in portions. The mixture was warmed to r.t. and stirred for 1 hour. 1-Chloro-2-methoxyethane (4.37 mL, 47.11 mmol) was then added, and the resultant mixture stirred at r.t. for a further 2 hours. The reaction was diluted with brine (100 mL) and extracted with EtOAc three times. The organic layer was washed with brine three times, dried and evaporated. Column chromatography with 19:1 hexanes/EtOAc afforded 3-(ethoxymethoxy)-2,6-dimethoxypyridine (**S51**), 8.14 g, 93%. ^1^H NMR (CDCl_3_) δ 7.41-7.33 (1H, m), 6.26-6.17 (1H, m), 5.15 (2H, d, J = 1.9 Hz), 3.98 (3H, d, J = 1.8 Hz), 3.87 (3H, d, J = 2.0 Hz), 3.77 (2H, dq, J = 1.8, 7.1 Hz), 1.22 (3H, dt, J = 2.9, 7.0 Hz).

To a solution of **S51** (4.00 g, 18.78 mmol) and *N*,*N*-diisopropylamine (0.13 mL, 0.94 mmol) in THF (60 mL, dist. Na) at -40 °C under nitrogen was added *n*-BuLi (2.0 M in cyclohexane, 14.09 mL, 28.17 mmol) dropwise. The resultant solution was stirred at -40 °C for 5 min, and then warmed to 0 °C and stirred at this temperature for a further 3 hours. The solution was then again cooled to -40 °C, and 1-formylpiperidine (3.75 mL, 33.80 mmol) was added dropwise, and the mixture stirred at r.t. for another 1 hour. Acetic acid (7.5 mL) was added and the solvent was removed in vacuo. The resultant mixture was partitioned between EtOAc and water, and the organic fraction dried and evaporated. Column chromatography with 19:1 hexanes/EtOAc afforded 3-(ethoxymethoxy)-2,6-dimethoxyisonicotinaldehyde (**S52**)**,** 2.30 g, 51%. ^1^H NMR (CDCl_3_) δ 10.39 (1H, s), 6.61 (1H, s), 6.19 (2H, s), 4.02 (3H, s), 3.88 (3H, s), 3.78 (2H, q, J = 10.1 Hz), 1.21 (3H, t, J = 7.1 Hz).

A solution of **S52** (2.30 g, 9.54 mmol) and 3M hydrochloric acid (60 mL) in THF (30 mL, dist. Na) was heated at 40 °C for 1.5 hours. The solution was then cooled, diluted with water, and the pH adjusted to 7 using NaHCO_3_. The aqueous layer was then extracted with EtOAc three times, and the organic layer dried and evaporated. Column chromatography with 19:1 hexanes/EtOAc afforded 3-hydroxy-2,6-dimethoxyisonicotinaldehyde (**S53**), (1.36 g, 78%. ^1^H NMR (CDCl_3_) δ 9.96 (1H, s), 9.61 (1H, s), 6.46 (1H, s), 4.06 (3H, s), 3.91 (3H, s).

A mixture of **S53** (1.35 g, 7.38 mmol) and potassium carbonate (1.53 g, 11.07 mmol) in DMF (40 mL, anhydrous) was heated at 50 °C for 10 min. Methyl iodide (0.56 mL, 8.86 mmol) was then added and the mixture stirred at this temperature for 2 hours. The resultant solution was diluted with EtOAc and washed with brine three times. The organic layer was dried and evaporated to afford the product 2,3,6-trimethoxyisonicotinaldehyde (**S54**), (1.40 g, 96%. ^1^H NMR (CDCl_3_) δ 10.40 (1H, s), 6.58 (1H, s), 4.04 (3H, s), 3.93 (3H, s), 3.91 (3H, s).

A mixture of **S54** (1.40 g, 7.11 mmol) and sodium borohydride (0.54 g, 14.21 mmol) in MeOH (30 mL, anhydrous) was stirred at r.t. for 1 hour. The solvent was then removed and the residue partitioned between EtOAc and water. The organic layer was dried and evaporated to afford the product (2,3,6-trimethoxypyridin-4-yl)methanol (**S55**)**,** (1.35 g, 95%. ^1^H NMR (CDCl_3_) δ 6.30 (1H, s), 4.68 (2H, d, J = 5.6 Hz), 3.99 (3H, s), 3.88 (3H, s), 3.79 (3H, s), 2.21 (1H, t, J = 5.9 Hz).

To a solution of **S55** (1.35 g, 6.78 mmol) and triethylamine (1.42 mL, 10.78 mmol) in DCM (20 mL, anhydrous) at r.t. was added mesyl chloride (0.63 mL, 8.14 mmol) dropwise. After 15 min, the reaction was diluted with DCM (20 mL) and the organic layer washed with sat. aq. NaHCO_3_, dried and evaporated. The residue was dissolved in acetone (40 mL, anhydrous), lithium bromide (excess) added, and the mixture heated at reflux for 30 min. The solution was then cooled and the solvent evaporated, and the residue partitioned between EtOAc and water. The aqueous layer was extracted twice with EtOAc and the organic layer was dried and evaporated to give the product 4-(bromomethyl)-2,3,6-trimethoxypyridine (**S56**) (1.69 g, 95%. ^1^H NMR (CDCl_3_) δ 6.27 (1H, s), 4.40 (2H, s), 3.98 (3H, s), 3.87 (3H, s), 3.87 (3H, s). Found: [M+H]=262.5

A mixture of (6-bromo-2-methoxyquinolin-3-yl)boronic acid (1.89 g, 6.69 mmol), **S56** (1.67 g, 6.37 mmol) and cesium carbonate (4.15 g, 12.74 mmol) in toluene (40 mL, anhydrous) and DMF (20 mL, anhydrous) was purged with nitrogen. Pd(PPh_3_)_4_ (0.29 g, 0.26 mmol) was then added, the mixture purged with nitrogen then heated to 80 °C under nitrogen for 4 hours. The reaction was partitioned between EtOAc and water and the organic fraction was dried and evaporated. Column chromatography (19:1 hexanes/EtOAc) gave the product 6-bromo-2-methoxy-3-((2,3,5-trimethoxypyridin-4-yl)methyl)quinoline (**AB-14**), 1.44 g, 54%. ^1^H NMR (CDCl_3_)  7.76 (1H, d, J = 2.2 Hz), 7.68 (1H, d, J = 8.9 Hz), 7.61 (1H, dd, J = 2.2, 8.8 Hz), 7.54 (1H, s), 6.04 (1H, s), 4.07 (3H, s), 4.00 (3H, s), 4.39 (2H, s), 3.85 (3H, s), 3.72 (3H, s). Found: [M+H]=420.0

**6-Bromo-3-((2-ethoxy-6-isopropoxypyridin-4-yl)methyl)-2-methoxyquinoline (AB-15)**

Potassium carbonate (8.65 g, 125 mmol) and 2-iodopropane (12.8 mL, 128 mmol) were added to a solution of ethyl 2-ethoxy-6-hydroxyisonicotinate (**S57**) (10.82 g, 51.2 mmol) in anhydrous DMF (125 mL) and the mixture was stirred at room temperature for 48 h. 2-Iodopropane (12.8 mL, 128 mmol) and potassium carbonate (8.65 g, 125 mmol) were added and the mixture was stirred for a further 24 h and then partitioned between DCM and water. The organic fraction was dried and evaporated, chromatography using DCM as an eluent gave ethyl 2-ethoxy-6-isopropoxyisonicotinate (**S58**) (11.557 g, 89%) as a colourless oil. ^1^H NMR (CDCl_3_) δ 6.81 (s, 2H), 5.23 (sp, *J* = 6.2 Hz, 1H), 4.36 (t, *J* = 7.2 Hz, 2H), 4.32 (t, *J* = 7.1 Hz, 2H), 1.33-1.42 (m, 12H). Found: [M+H]=254.2.

A solution of LiOH (3.25 g, 136 mmol) in water (60 mL) was added to a solution of **S58** (11.424 g, 45.1 mmol) in THF (60 mL) and MeOH (60 mL), the solution was stirred at room temperature for 60 h then evaporated. The residue was dissolved in water (200 mL) and the solution was adjusted to pH 6 with 2 M HCl. The oily solid was extracted with EtOAc, the organic fractions were washed with water, dried and evaporated to give 2-ethoxy-6-isopropoxyisonicotinic acid (**S59**) (10.034 g, 99%) as a white solid. M.p. >300 °C. ^1^H NMR (DMSO-d^6^) δ 13.48 (bs, 1H), 6.66 (d, *J* = 1.0 Hz, 1H), 6.64 (d, *J* = 1.0 Hz, 1H), 5.17 (sp, *J* = 6.2 Hz, 1H), 4.50 (q, *J* = 7.0 Hz, 2H), 1.28-1.34 (m, 9H). No diagnostic peak in the mass spectrum.

Trimethylborate (3.03 mL, 26.7 mmol) and then borane-dimethylsulfide (2.53 mL, 26.7 mmol) were added to a solution of **S59** (3.00 g, 13.3 mmol) in THF (50 mL, dist. Na) at 0 °C and the mixture was stirred at r.t. for 18 hr. The solution was cooled to 0 °C and methanol was cautiously added to quench the reaction. Removal of the solvent gave a solid, this was partitioned between EtOAc and water, and the organic fraction was dried and evaporated. Column chromatography (3:1 hexanes:EtOAc) gave (2-ethoxy-6-isopropoxypyridin-4-yl)methanol (**S60**) (2.72 g, 97%) as a colourless oil. ^1^H NMR (CDCl_3_) δ 6.24 (s, 1H), 6.23 (s, 1H), 5.21 (sp, *J* = 6.2 Hz, 1H), 4.60 (d, *J* = 6.2 Hz, 2H), 4.30 (q, *J* = 7.0 Hz, 2H), 1.70 (t, *J* = 6.2 Hz, 1H), 1.38 (t, *J* =7.0 Hz, 3H), 1.34 (d, *J* = 6.2 Hz, 6H). Found: [M+H]=212.2.

A solution of **S60** (2.60 g, 12.3 mmol) in DCM (50 mL, anhydrous) at 0 °C was treated sequentially with triethylamine (3.43 mL, 24.6 mmol) then mesyl chloride (1.43 mL, 18.5 mmol), the mixture was stirred at 0 °C for 1 h then partitioned between DCM and water. The organic fraction was dried and evaporated and the residue was dissolved in acetone (100 mL), LiBr (10.7 g, 123 mmol) was added and the mixture was refluxed for 1 h and then evaporated. The residue was partitioned between DCM and water; the organic fraction was dried and evaporated. Column chromatography (DCM) gave 4-(bromomethyl)-2-ethoxy-6-isopropoxypyridine (**S61**) (3.04 g, 100%) as a colourless oil. ^1^H NMR (CDCl_3_) δ 6.26 (s, 1H), 6.25 (s, 1H), 5.20 (sp, *J* = 6.2 Hz, 1H), 4.26-4.33 (m, 4H), 1.38 (t, *J* = 7.0 Hz, 3H), 1.34 (d, *J* = 6.2 Hz, 6H). Found: [M+H]=274.1.

A mixture of (6-bromo-2-methoxyquinolin-3-yl)boronic acid (3.27 g, 11.6 mmol), (**S61**) (3.18 g, 11.6 mmol) and Cs_2_CO_3_ (7.56 g, 23.2 mmol) in toluene (66 mL) and DMF (33 mL) was purged with nitrogen. Pd(PPh_3_)_4_ (0.27 g, 0.23 mmol) was added, the mixture was purged with nitrogen then heated to 80 °C under nitrogen for 3 h. The reaction was partitioned between EtOAc and water and the organic fraction was dried and evaporated. Column chromatography with 3:1 hexanes:DCM eluted impurities, then elution with DCM gave 6-bromo-3-((2-ethoxy-6-isopropoxypyridin-4-yl)methyl)-2-methoxyquinoline (**AB-15**) (3.42 g, 68%) as white solid. M.p. 78-80 °C. ^1^H NMR (CDCl_3_) δ 7.77 (d, *J* = 2.1 Hz, 1H), 7.68 (d, *J* = 8.8 Hz, 1H), 7.62 (dd, *J* = 8.8, 2.2 Hz, 1H), 7.58 (s, 1H), 6.11 (s, 1H), 6.10 (s, 1H), 5.20 (sp, *J* = 6.2 Hz, 1H), 4.28 (q, *J* = 7.1 Hz, 2H), 4.06 (s, 3H), 3.89 (s, 2H), 1.36 (t, *J* = 7.1 Hz, 3H), 1.32 (d, *J* = 6.2 Hz, 6H). Found: [M+H]=431.1.

**6-Bromo-3-((2-cyclobutoxy-6-methoxypyridin-4-yl)methyl)-2-methoxyquinoline (AB-16)**

A solution of methyl 2-hydroxy-6-methoxyisonicotinate (**S62**) (3.00 g, 16.4 mmol) in DMF (50 mL, anhydrous) was treated with K_2_CO_3_ (4.52 g, 32.7 mmol) and bromocyclobutane (2.00 mL, 25.0 mmol). The mixture was stirred at room temperature for 48 h, partitioned between EtOAc and water and the aqueous layer was extracted with EtOAc. The combined organic fractions were washed with water, dried and evaporated. Column chromatography (DCM) gave methyl 2-cyclobutoxy-6-methoxyisonicotinate (**S63**) (2.215 g, 57%) as a colourless oil. ^1^H NMR (CDCl_3_) δ 6.84 (d, *J* = 1.0 Hz, 1H), 6.79 (d, *J* = 1.0 Hz, 1H), 5.08 (pd, *J* = 7.4, 0.8 Hz, 1H), 3.91 (s, 3H), 3.90 (s, 3H), 2.42-2.52 (m, 2H), 2.12-2.24 (m, 2H), 1.80-1.90 (m, 1H), 1.62-1.75 (m, 1H). Found: [M + H] = 238.2.

A solution of LiOH (0.71 g, 29.6 mmol) in water (20 mL) was added to a solution of **S63** (2.205 g, 9.29 mmol) in MeOH (20mL) and THF (20 mL); the solution was stirred at room temperature for 18 h and then evaporated. The residue was dissolved in water (80 mL) and acidified to pH 3 with 2 M HCl. The resulting precipitate was filtered and dried to give 2-cyclobutoxy-6-methoxyisonicotinic acid (**S64**) (2.02 g, 97%) as a white solid. M.p. 170-171 °C. ^1^H NMR (DMSO-d^6^) δ 13.56 (bs, 1H), 6.74 (d, *J* = 1.0 Hz, 1H), 6.67 (d, *J* = 1.0 Hz, 1H), 5.07 (pd, *J* = 7.1, 0.7 Hz, 1H), 3.85 (s, 3H), 2.37-2.46 (m, 2H), 2.14-2.22 (m, 2H), 1.74-1.83 (m, 1H), 1.59-1.72 (m, 1H). Found: [M + H] = 224.2.

Trimethyl borate (1.03 mL, 9.0 mmol) and borane dimethyl sulphide complex were added sequentially to a solution of **S64** (1.01 g, 4.52 mmol) in anhydrous THF (20 mL) at 0 °C. The solution was stirred at room temperature for 18 h and then quenched with MeOH. Removal of the solvent gave an oil, chromatography (3:1 hexanes:EtOAc) of the crude product gave (2-cyclobutoxy-6-methoxypyridin-4-yl)methanol (**S65**) (0.91 g, 96%) as a colourless oil. ^1^H NMR (CDCl_3_) δ 6.29 (d, *J* = 0.9 Hz, 1H), 6.23 (d, *J* = 0.9 Hz, 1H), 5.06 (pd, *J* = 7.2, 0.9 Hz, 1H), 4.62 (d, *J* = 6.2 Hz, 2H), 3.88 (s, 3H), 2.41-2.49 (m, 2H), 2.11-2.22 (m, 2H), 1.79-1.89 (m, 1H), 1.63-1.75 (m, 2H). Found: [M + H] = 210.2

A solution of **S65** 0.842 g, 4.04 mmol) in DCM (25 mL) at 0 °C was treated sequentially with Et_3_N (2.25 mL, 16.1 mmol) and mesyl chloride (0.47 mL, 6.1 mmol), the solution was stirred for 1 h at 0 °C and then partitioned with water, the organic fraction was dried and evaporated. The residue was dissolved in acetone (50 mL), LiBr (3.50 g, 40.3 mmol) was added and the mixture was refluxed for 0.5 h and then evaporated. The residue was partitioned between DCM and water and the organic fraction was dried and evaporated. Column chromatography (DCM) gave 4-(bromomethyl)-2-cyclobutoxy-6-methoxypyridine (**S66**) (0.994 g, 90%) as a colourless oil. ^1^H NMR (CDCl_3_) δ 6.30 (d, *J* = 1.0 Hz, 1H), 6.25 (d, *J* = 1.0 Hz, 1H), 5.07 (pd, *J* = 7.1, 0.9 Hz, 1H), 4.27 (s, 2H), 3.88 (s, 3H), 2.41-2.49 (m, 2H), 2.11-2.22 (m, 2H), 1.79-1.89 (m, 1H), 1.61-1.75 (m, 1H). Found: [M + H] = 272, 274.

A mixture of (6-bromo-2-methoxyquinolin-3-yl)boronic acid (1.010 g, 3.58 mmol), **S66** (0.975 g, 3.58 mmol) and Cs_2_CO_3_ (2.33 g, 7.2 mmol) in toluene/DMF (2:1, 50 mL) was purged with nitrogen. Pd(PPh_3_)_4_ (0.083 g, 0.072 mmol) was added and the mixture was heated to 80 °C for 3h under an atmosphere of nitrogen. The mixture was partitioned between EtOAc and water, the organic fraction was dried and evaporated. Column chromatography using a gradient of 3:1 hexanes:DCM to DCM gave **AB-16** (1.195 g, 78%) as a white solid. M.p. 101-102 ^o^C. ^1^H NMR (CDCl_3_) δ 7.77 (d, *J* = 2.2 Hz, 1H), 7.69 (d, *J* = 8.9 Hz, 1H), 7.62 (dd, *J* = 8.9, 2.2 Hz, 1H), 7.52 (s, 1H), 6.16 (d, *J* = 0.3 Hz, 1H), 6.10 (d, *J* = 0.3 Hz, 1H), 5.06 (pd, *J* = 7.9, 0.9 Hz, 1H), 4.06 (s, 3H), 3.90 (s, 2H), 3.87 (s, 3H), 2.38-2.48 (m, 2H), 2.09-2.21 (m, 2H), 1.77-1.87 (m, 1H), 1.62-1.72 (m, 1H). Found: [M + H] = 429.1, 431.1.

**3-((2,6-Bis(methylthio)pyridin-4-yl)methyl)-6-bromo-2-methoxyquinoline (AB-17)**

2,6-Dichloroisonicotinic acid (**S67**) (4.00 g, 20.8 mmol) in DMF (40 ml) was added sodium thiomethoxide (4.38 g, 65.5 mmol) at 0 °C in batches. Mixture was stirred at 150 °C for 18 h. Water (40 ml) was added to the resultant solution and pH was adjusted to 3 using 2M HCl solution. Extracted with EtOAc (3x), dried with MgSO_4_, filtered and the solvent was evaporated to give 2,6-bis(methylthio)isonicotinic acid (**S68**) as an orange solid. Yield = 4.01 g, 90%. MP = 95 – 97 °C. The solid was recrystallized from methanol and was used without further purification for the next step.  ^1^H NMR (CDCl_3_) δ 7.44 (s, 2H), 2.61 (s, 6H). Found: [M+H]=216.5.

Borane dimethyl sulfide complex (8.09 ml, 85.3 mmol) and trimethyl borate (9.68 ml, 85.3 mmol) were added to a solution of **S68** (6.12 g, 28.4 mmol) in THF (100 mL, dist. Na) at 0 °C. The solution was then warmed to room temperature and was stirred for 21 h at r.t. The reaction mixture was cooled to 0 °C, MeOH (20 ml) was added to quench the reaction and the solvent was evaporated in the fume hood. The residue was partitioned between EtOAc and water, extracted with EtOAc (3x), dried with MgSO_4_, filtered and the solvent was evaporated. Column chromatography (4:1 hexanes:EtOAc) gave (2,6-bis(methylthio)pyridin-4-yl)methanol (**S69**) as a white solid. Yield = 4.56 g, 80%. MP = 88 – 90 °C. ^1^H NMR (CDCl_3_) δ 6.87 (s, 2H), 4.61 (s, 2H), 2.58 (s, 6H). Found: [M+H]=202.5.

Phosphorus tribromide (2.97 ml, 31.4 mmol) was added to a solution of **S69** (5.26 g, 26.1 mmol) in DCM (300 mL) at 0 °C. The reaction mixture was stirred at r.t. for 18 h then solvent was evaporated. The residue was diluted with DCM (100 mL) and quenched with ice, the organic layer was washed with sat. aq. NaHCO_3_, dried with MgSO_4_, filtered and the solvent was evaporated. Column chromatography (6:1 hexanes:EtOAc) gave 4-(bromomethyl)-2,6-bis(methylthio)pyridine (**S70**) as a white solid. Yield = 3.44 g, 50%. MP = 100 – 102 °C. ^1^H NMR (CDCl_3_) δ 6.86 (s, 2H), 4.23 (s, 2H), 2.58 (s, 6H). Found: [M+H]=266.4.

A mixture of **S70** (3.44 g, 15.0 mmol), (6-bromo-2-methoxyquinolin-3-yl)boronic acid (4.31 g, 15.0 mmol), and Cs_2_CO_3_ (11.24 g, 34.5 mmol) in toluene-DMF (60 ml, 2:1) was degassed under N_2_, then added Pd(PPh_3_)_4_ (0.867 g, 0.750 mmol), and heated at 90 °C for 2 h. Reaction mixture was cooled to r.t., filtered through a plug of celite, added water and extracted with EtOAc (x4). Organic layer washed with brine, dried with Na_2_SO_4_, filtered and the solvent was evaporated to give a yellow residue. Purification by flash column chromatography with silica using hexane:EtOAc (9:1) to gave 3-((2,6-bis(methylthio)pyridin-4-yl)methyl)-6-bromo-2-methoxyquinoline (**AB-17**) as a white solid. Yield = 2.90 g, 46%. MP = 129 – 131 °C. ^1^H NMR (CDCl_3_) δ 7.80 (d, J = 2.1 Hz, 1H), 7.70 (d, J = 8.9 Hz, 1H), 7.65 (dd, J = 8.9, 2.2 Hz), 7.57 (s, 1H), 6.72 (s, 2H), 4.05 (s, 3H), 3.87 (s, 2H), 2.57 (s, 6H). Found: [M+H]=421.8.

**3-((2,6-Bis(ethylthio)pyridin-4-yl)methyl)-6-bromo-2-methoxyquinoline (AB-18)**

Sodium hydride (60% w/w, 1.44 g, 36.1 mmol) in DMF (25 ml) at 0 °C was added ethanethiol (2.70 ml, 36.1 mmol) dropwise. The mixture foamed up as hydrogen gas was released. The mixture was stirred at 0 °C for 15 minutes, then a solution of 2,6-dichloroisonicotinic acid (**S67**) (2.05 g, 11.6 mmol) in anhydrous DMF (9 mL) was added dropwise at 0 °C. The mixture was then stirred at 150 °C for 17 hours. The mixture was diluted in water, dissolving the white salts. The aqueous mixture was purged with air to remove most of the stench. 2M hydrochloric acid was added until pH reached ~3, when white solids crashed out. The solids were collected by filtration, washed with ice-cold water, dried to yield 2,6-bis(ethylthio)isonicotinic acid (**S71**) as light yellow solid which was used directly in the next step. Yield = 2.82 g, 100%. ^1^H NMR (CDCl_3_) δ 7.40 (s, 2H), 3.19 (q, J = 7.3 Hz, 4H), 1.39 (t, J = 7.3 Hz, 6H). Found: [M+H]=244.5.

Borane dimethyl sulfide complex (3.30 ml, 34.8 mmol) and trimethyl borate (3.90 ml, 34.8 mmol) were added to a solution of **S71** (2.82 g, 11.6 mmol) in THF (100 mL, dist. Na) at 0 °C. The solution was then warmed to room temperature and was stirred for 18 h at r.t. The reaction mixture was cooled to 0 °C, MeOH (20 ml) was added to quench the reaction and the solvent was evaporated in the fume hood. The residue was partitioned between EtOAc and water, extracted with EtOAc (3x), dried with MgSO_4_, filtered and the solvent was evaporated. Column chromatography (4:1 hexanes:EtOAc) gave (2,6-bis(ethylthio)pyridin-4-yl)methanol (**S72**) as a white solid. Yield = 2.50 g, 94%. ^1^H NMR (CDCl_3_) δ 6.87 (s, 2H), 4.61 (s, 2H), 2.58 (s, 6H). Found: [M+H]=202.5.

To a solution of **S72** (2.48 g, 10.8 mmol) in anhydrous DCM (42 mL) was added triethylamine (2.20 mL, 16.2 mmol) dropwise, followed by methanesulfonyl chloride (1.10 mL, 14.2 mmol). The mixture was stirred at 0 °C for 10 minutes, then allowed to stir at room temperature for 1 hour. The mixture was quenched with saturated sodium bicarbonate solution. The aqueous mixture was extracted with dichloromethane (2x). The combined extract was washed with brine, dried with MgSO_4_ and concentrated to afford the crude product as a yellow oil. The crude intermediate was diluted in acetone (84 mL). Lithium chloride (1.85 g, 43.64 mmol) was added. The suspension was stirred at room temperature overnight. The mixture was concentrated in vacuo and adsorbed onto silica. Flash chromatography using a mixture of 98:2 hexane/ethyl acetate gave 4-(chloromethyl)-2,6-bis(ethylthio)pyridine (**S73**) as a light yellow oil. Yield = 2.26 g, 84%. ^1^H NMR (CDCl_3_) δ 6.87 (s, 2H), 4.61 (s, 2H), 2.58 (s, 6H). Found: [M+H]=202.5.

A mixture of (6-bromo-2-methoxyquinolin-3-yl)boronic acid (2.56 g, 9.10 mmol), **S73** (2.25 g, 9.10 mmol) and Cs_2_CO_3_ (5.93 g, 18.19 mmol) in toluene-DMF (60 ml, 2:1) was degassed under N_2_, then added Pd(PPh_3_)_4_ (0.52 g, 0.45 mmol), and heated at 90 °C for 1.5 h. Reaction mixture was cooled to r.t., filtered through a plug of celite, added water and extracted with EtOAc (x4). Organic layer washed with brine, dried with Na_2_SO_4_, filtered and the solvent was evaporated to give a yellow residue. Purification by flash column chromatography with silica using hexane:EtOAc (9:1) gave 3-((2,6-bis(ethylthio)pyridin-4-yl)methyl)-6-bromo-2-methoxyquinoline (**AB-18**) as a white solid. Yield = 1.80 g, 47%. ^1^H NMR (CDCl_3_) δ 7.80 (d, J = 2.1 Hz, 1H), 7.70 (d, J = 8.9 Hz, 1H), 7.65 (dd, J = 8.9, 2.2 Hz), 7.57 (s, 1H), 6.72 (s, 2H), 4.05 (s, 3H), 3.87 (s, 2H), 2.57 (s, 6H). Found: [M+H]=421.8.

**4-((6-Bromo-2-methoxyquinolin-3-yl)methyl)-6-(ethylthio)-N,N-dimethylpyridin-2-amine (AB-19)**

To a glass tube was charged methyl 2-chloro-6-(dimethylamino)isonicotinate (**S74**) (WO 2010/100475) (2.44 g, 11.40 mmol), rac-bis(diphenylphosphino)-1,1'-binaphthol (0.71 g, 1.140 mmol) and cesium carbonate (4.43 g, 13.60 mmol) under continuous nitrogen flow. Anhydrous toluene (30 mL) was added. The mixture was purged with nitrogen for 5 min Palladium acetate (0.26 g, 1.158 mmol) was added and the mixture was purged again with nitrogen. Ethanethiol (1.0 mL, 13.60 mmol) was added and the mixture was sealed in the tube and heated at 150 °C for 22 hours. The mixture was filtered through Celite, washing with EtOAc. The filtrate was concentrated in the fume hood by heating the solution while purging with air. A crude orange solid was obtained. Flash chromatography of the product using 2-5% diethyl ether in hexanes provided the product methyl 2-(dimethylamino)-6-(ethylthio)isonicotinate (**S75**) as a yellow crystalline solid. Yield = 2.42 g, 88%. %. ^1^H NMR (CDCl_3_) δ 6.96 (1H, d, J = 1.0 Hz), 6.73 (1H, d, J = 1.0 Hz), 3.89 (3H, s), 3.15 (2H, q, 7.3 Hz), 3.12 (6H, s), 1.38 (3H, t, J = 7.3 Hz). Found: [M+H]=241.5.

To a solution of **S75** (1.64 g, 6.824 mmol) in freshly distilled THF (66 mL) was added at 2 °C under nitrogen lithium aluminium hydride (0.31 g, 8.189 mmol) in 3 batches. The mixture was stirred at 2 °C for 15 min then at r.t. for 1 hour. The mixture was quenched cautiously with water at 2 °C until gas evolution ceased. 1M NaOH (20 mL) was added. The mixture was stirred for 5 min, then decanted leaving the aluminium salts which were filtered through Celite. The aqueous mixture was partitioned between water and EtOAc. The aqueous phase was extracted with EtOAc (3x). The combined organic extract was washed with brine, dried (Na_2_SO_4_) and concentrated to give the crude product as a brown oil. The crude product was purified by flash chromatography using a 4:1 mixture of hexanes/EtOAc to afford the product (2-(dimethylamino)-6-(ethylthio)pyridin-4-yl)methanol (S76) as a brown oil. Yield = 1.30 g, 90%. ^1^H NMR (CDCl_3_) δ 6.43 (1H, s), 6.18 (1H, d, J = 0.8 Hz), 4.56 (2H, d, J = 5.0 Hz), 3.14 (2H, q, 7.4 Hz), 3.08 (6H, s), 1.67 (1H, br t, J = 5.8 Hz), 1.57 (3, s), 1.37 (3H, t, J = 7.3 Hz). Found: [M+H]=213.5.

To a solution of **S76** (0.60 g, 2.84 mmol) in anhydrous DCM (10 mL) was added at 2 ^o^C under nitrogen triethylamine (0.59 mL, 4.26 mmol) dropwise, followed by mesyl chloride (0.26 mL, 3.41 mmol). The mixture was stirred from 2 °C to 5 °C over 1 hour. The mixture was quenched with sat. aq. NaHCO_3_ solution. The aqueous mixture was extracted with DCM (3x). The combined extract was washed with brine, dried (MgSO_4_) and concentrated to afford the crude product as a light brown oil. The crude intermediate was dissolved in acetone (20 mL). Lithium bromide (0.99 g, 11.36 mmol) was added and the suspension was stirred at r.t. for 2.5 hours. Flash chromatography using 98:2 hexanes/Et_2_O furnished the product 4-(bromomethyl)-6-(ethylthio)-N,N-dimethylpyridin-2-amine (**S77**) as a brown solid. The reaction was repeated on a 3.29 mmol scale and the products were combined. Total yield = 1.39 g, 82%. ^1^H NMR (CDCl_3_) δ 6.46 (1H, d, J = 1.2 Hz), 6.15 (1H, d, J = 0.8 Hz), 4.24 (2H, s), 3.13 (2H, q, J = 7.2 Hz), 3.08 (6H, s), 1.37 (3H, t, J = 7.2 Hz). Found: [M+H]=275.5

A mixture of (6-bromo-2-methoxyquinolin-3-yl)boronic acid (1.42 g, 5.05 mmol), (**S77**) (1.39 g, 5.05 mmol) and cesium carbonate (3.29 g, 10.09 mmol) in a mixture of toluene (14 mL) and DMF (7 mL) was purged with nitrogen. Pd(PPh_3_)_4_ (0.29 g) was added. The mixture was purged again with nitrogen and heated at 85 °C under nitrogen for 2.5 hours. The mixture was partitioned between water and EtOAc. The aqueous mixture was extracted with EtOAc (2x). The extract was washed with water, brine, dried (MgSO4) and concentrated to afford the crude product as an orange oil which was chromatographed using 2-5% Et_2_O in hexanes as eluent to yield the product 4-((6-bromo-2-methoxyquinolin-3-yl)methyl)-6-(ethylthio)-N,N-dimethylpyridin-2-amine (**AB-19**) as a light yellow solid. Recrystallisation from DCM/MeOH provided a white solid. Yield = 1.25 g, 57%. ^1^H NMR (CDCl_3_) δ 7.79 (1H, d, J = 2.4 Hz), 7.70 (1H, d, J = 8.8 Hz), 7.64 (1H, dd, J = 2.4, 9.2 Hz), 7.56 (1H, s), 6.34 (1H, s), 6.06 (1H, s), 4.09 (3H, s), 3.86 (2H, s), 6.15 (2H, q, J = 7.2 Hz), 3.07 (6H, s), 1.39 (3H, t, J = 7.2 Hz). Found [M+H]=432.1

**6-Bromo-3-((3-fluoro-2-methoxypyridin-4-yl)methyl)-2-methoxyquinoline (AB-20)**

To a solution of n-BuLi (26.1 mL, 52.09 mmol) in THF (70 mL, dist. Na) at -78°C was added consecutively *N*,*N*-diisopropylamine (7.30 mL, 52.09 mmol) and 2,3-difluoropyridine (**S78**) (5.00 g, 43.41 mmol).The resultant mixture was stirred at -78°C for 1 hour, and then poured on crushed dry ice (excess). The reaction was allowed to warm to r.t. for 1 hour, and after evaporation of excess dry ice and THF, the residue was taken up into water (100 mL) and washed with EtOAc (2 x 50 mL). The aqueous layer was then acidified to pH 1 and extracted with EtOAc (2 x 100 mL). The combined organic extracts were dried and evaporated to afford 2,3-difluoroisonicotinic acid (**S79**) (2.39 g, 35%). ^1^H NMR (CDCl_3_) δ 15.3-13.2 (1H, br s), 8.14 (1H, dd, J = 1.2, 5.0 Hz), 7.70 (1H, dd, J = 4.8, 4.8 Hz). Found: [M-H]=158.5.

Sodium (0.79 g, 33.09 mmol) was added portion wise to MeOH (60 mL) over 0.5 h. **S79** (2.39 g, 15.04 mmol) was then added and the reaction refluxed for 2 hours. The solution was cooled and the solvent evaporated. The residue was taken up into water (100 mL) and washed with EtOAc (2 x 50 mL). The aqueous layer was then acidified to pH 1 and extracted with EtOAc (2 x 100 mL). The combined organic extracts were dried and evaporated to afford the product 3-fluoro-2-methoxyisonicotinic acid (**S80**), 2.23 g, 87%. ^1^H NMR (DMSO-d_6_) δ 14.6-12.8 (1H, br s), 8.05 (1H, d, J = 5.2 Hz), 7.28 (1H, dd, J = 4.7, 4.9 Hz), 3.97 (3H, s). Found: [M-H]=170.5.

Borane−dimethylsulfide complex (2.47 mL, 26.07 mmol) and trimethyl borate (2.96 mL, 26.07 mmol) were added to a solution of **S80** (13.03 mmol) in THF (80 mL, dist. Na) at 0°C, and the solution warmed to r.t. and stirred overnight. The mixture was then cooled to 0°C, and quenched with methanol (10 mL). The solvent was then evaporated and the residue was partitioned between EtOAc and water. The organic layer was then dried and evaporated to afford the product (3-fluoro-2-methoxypyridin-4-yl)methanol (**S81**), 1.89 g, 92%. ^1^H NMR (CDCl_3_) δ 7.92 (1H, d, J = 5.2 Hz), 7.02 (1H, dd, J = 4.5, 5.1 Hz), 4.81 (2H, s), 4.03 (3H, s). Found: [M+H]=158.5.

To a solution of **S81** (1.89 g, 12.03 mmol) and triethylamine (2.52 mL, 18.05 mmol) in DCM (30 mL, anhydrous) at r.t. was added mesyl chloride (1.12 mL, 14.44 mmol) dropwise. After 15 min, the reaction was diluted with DCM (20 mL) and the organic layer washed with sat. NaHCO_3_, dried and evaporated. The residue was dissolved in acetone (60 mL, anhydrous), lithium bromide (excess) added, and the mixture heated at reflux for 30 min. The solution was then cooled and the solvent evaporated, and the residue partitioned between EtOAc and water. The aqueous layer was extracted twice with EtOAc and the organic layer was dried and evaporated to give the product 4-(bromomethyl)-3-fluoro-2-methoxypyridine (**S82**), 2.20 g, 83% ^1^H NMR (CDCl_3_) δ 7.90 (1H, d, J = 5.2 Hz), 6.90 (1H, dd, J = 4.8, 4.9 Hz), 4.43 (2H, s), 4.02 (3H, s). Found: [M+H]=220.2

A mixture of (6-bromo-2-methoxyquinolin-3-yl)boronic acid (2.56 g, 9.09 mmol), **S82** (2.20 g, 10.00 mmol) and cesium carbonate (5.92 g, 18.18 mmol) in toluene (45 mL, anhydrous) and DMF (22.5 mL, anhydrous) was purged with nitrogen. Pd(PPh_3_)_4_ (0.42 g, 0.363 mmol) was then added, the mixture purged with nitrogen, then heated to 80 °C under nitrogen for 4 hours. The reaction was partitioned between EtOAc and water and the organic fraction was dried and evaporated. Column chromatography (19:1 hexanes/EtOAc) gave the product 6-bromo-3-((3-fluoro-2-methoxypyridin-4-yl)methyl)-2-methoxyquinoline (**AB-20**)**,** 1.76 g, 51%. ^1^H NMR (CDCl_3_) δ 7.82 (1H, d, J = 5.2 Hz), 7.79 (1H, d, J = 2.1 Hz), 7.69 (1H, d, J = 8.8 Hz), 7.6 4 (1H, dd, J = 2.2, 8.9 Hz), 7.60 (1H, s), 6.69 (1H, dd, J = 4.8, 5.0 Hz), 4.07 (3H, s), 4.06 (2H, s), 4.03 (3H, s). Found: [M+H]=377.2

**Scheme 2: Synthesis of dialkoxypyridyl Mannich bases (C/D units (III-VII)**

**Table 2: list and yields of new C/D units**

| **X** | **Y** | **No** |
| --- | --- | --- |
| Me | NMe_2_ | IIIa |
| Et | NMe_2_ | IIIb |
| Me | N(OMe)Me) (IV) | IV |
| Me | Nmorpholide (V) | V |
| Me | Nimidazole (VI) | VI |
| Me | Ntriazole VII) | VII |

**1-(2,6-Dimethoxypyridin-4-yl)-3-(dimethylamino)propan-1-one (IIIa)**

Oxalyl chloride (1.34 mL, 15.8 mmol) was added to a suspension of 2,6-dimethoxyisonicotinic acid (**4**) (2.41 g, 13.2 mmol) in DCM (70 mL) and DMF (0.20 mL, 2.6 mmol) at r.t.. The mixture was stirred for 1 h to give a colourless solution which was cooled to 0 °C. *N,O-*dimethylhydroxylamine hydrochloride (1.42 g, 14.6 mmol) and pyridine (3.51 mL, 28.9 mmol) were added sequentially and the mixture was stirred at r.t. for 18 h, then partitioned between EtOAc and sat. aq. NaHCO_3_. Column chromatography with hexanes:EtOAc (2:1) gave N,2,6-trimethoxy-N-methylisonicotinamide (**5**) (2.49 g, 83%). ^1^H NMR (CDCl_3_) δ 6.47 (s, 2H), 3.93 (s, 6H), 3.58 (br s, 3H), 3.32 (s, 3H). Found: [M+H]=227.2.

Vinylmagnesium bromide (32 mL of a 1N solution in THF, 32 mmol) was added to a solution of **5** (2.45 g, 10.8 mmol) in dry THF (100 mL) at 0 °C. The brown solution was warmed to r.t. for 1 h then dimethylamine (32 mL of a 2N solution in THF, 64 mmol) and water (30 mL) were added. The solution was stirred at r.t. for 1 h, then partitioned between EtOAc and water. The solution was dried and evaporated and column chromatography with DCM:MeOH (95:5) eluted impurities while DCM:MeOH (9:1) gave 1-(2,6-dimethoxypyridin-4-yl)-3-(dimethylamino)propan-1-one (**IIIa**) as an oil (0.81 g, 31%). ^1^H NMR (CDCl_3_) δ 6.74 (s, 2H), 3.95 (s, 6H), 3.06 (t, J = 7.0 Hz, 2H), 2.72 (t, J = 7.0 Hz, 2H), 2.27 (s, 6H). Found: [M+H]=239.1

**1-(2,6-Diethoxypyridin-4-yl)-3-(dimethylamino)propan-1-one (IIIb)**

Oxalyl chloride (0.73 mL, 8.6 mmol) was added to a suspension of 2,6-dichloroisonicotinic acid **(4**) (1.52 g, 7.20 mmol) in DCM (50 mL, anhydrous) and DMF (0.20 mL, 2.6 mmol) at r.t.. The mixture was stirred at r.t. for 1 h to give a colourless solution, which was then cooled to 0 °C. *N,O-*dimethylhydroxylamine hydrochloride (0.77 g, 17.89 mmol) and pyridine (1.92 mL, 23.7 mmol) were added sequentially and the mixture was stirred at r.t. for 18 h, then partitioned between EtOAc and sat. aq. NaHCO_3_. Column chromatography using 3:1 hexanes:EtOAc gave **5** (1.26 g, 69%). ^1^H NMR (CDCl_3_) δ 6.43 (s, 2H), 4.33 (q, J = 7.1 Hz, 2H), 3.59 (br s, 3H), 3.32 (s, 3H), 1.39 (t, J = 7.1 Hz, 3H). Found: [M+H]=255.1

Vinylmagnesium bromide (14.6 mL of a 1N solution in THF, 14.6 mmol) was added to a solution of **5** (1.23 g, 4.85 mmol) in dry THF (50 mL) at 0 °C. The brown solution was warmed to r.t. for 1 h then a solution of 2N dimethylamine in THF (14.6 mL, 29.2 mmol) and water (10 mL) were added. The solution was stirred at r.t. for 1 h, then partitioned between EtOAc and water. The solution was dried and evaporated to give **IIIb** as a brown oil (1.24 g, 96%). ^1^H NMR (CDCl_3_) δ 6.71 (s, 2H), 4.34 (q, J = 7.1 Hz, 2H), 3.05 (t, J = 7.0 Hz, 2H), 2.72 (t, J = 7.0 Hz, 2H), 2.26 (s, 6H), 1.40 (t, J = 7.0 Hz, 3H). Found: [M+H]=267.2

**1-(2,6-Dimethoxypyridin-4-yl)-3-(methoxy(methyl)amino)propan-1-one (IV)**

Vinylmagnesium bromide (62.7 mL of a 1N solution in THF, 62.7 mmol) was added to a solution of **5** (see above) (6.75 g, 29.8 mmol) in dry THF (150 mL) at 0 °C. The yellow/orange solution was warmed to r.t. for 1 h and water (30 mL) were added. The solution was stirred at r.t. for 24 h, then partitioned between EtOAc and water. The solution was dried and evaporated and column chromatography with hexanes:EtOAc (9:1) gave 1-(2,6-dimethoxypyridin-4-yl)-3-(methoxy(methyl)amino)propan-1-one (**IV**) as an oil (5.50 g, 72%). ^1^H NMR (CDCl_3_) δ 6.76 (s, 2H), 3.96 (s, 6H), 3.46 (s, 3H), 3.16 (t, J = 6.6 Hz, 2H), 3.04 (t, J = 6.5 Hz, 2H), 2.61 (s, 3H). Found: [M+H]= 255.6.

**1-(2,6-Dimethoxypyridin-4-yl)-3-morpholinopropan-1-one (V)**

Vinylmagnesium bromide (18.6 mL of a 1N solution in THF, 18.6 mmol) was added to a solution of **5** (see above) (2.00 g, 8.80 mmol) in dry THF (30 mL) at 0 °C. The yellow/orange solution was warmed to r.t. for 1 h then morpholine (3.23 mL, 37.1 mmol) then water (10 mL) were added. The solution was stirred at r.t. for 1 h, the solvent removed in vacuo, and the resultant mixture then partitioned between EtOAc and water. The solution was dried and evaporated to afford 1-(2,6-dimethoxypyridin-4-yl)-3-morpholinopropan-1-one (**V**) as an oil (2.40 g, 97%). ^1^H NMR (CDCl_3_) δ 6.73 (s, 2H), 3.95 (s, 6H), 3.70 (t, J = 4.4 Hz, 4H), 3.08 (t, J = 7.1 Hz, 2H), 2.79 (t, J = 7.4 Hz, 2H), 2.48 (t, J = 4.4 Hz, 4H). Found: [M+H]= 281.6.

**1-(2,6-Dimethoxypyridin-4-yl)-3-(1H-imidazol-1-yl)propan-1-one (VI)**

Vinylmagnesium bromide (0.93 mL of a 1N solution in THF, 0.93 mmol) was added to a solution of **5** (see above) (0.10 g, 0.44 mmol) in dry THF (3 mL) at 0 °C. The yellow/orange solution was warmed to r.t. for 1 h. The solvent was removed *in vacuo* and the residue partitioned between EtOAc and water, and the pH adjusted to 3 with 1M HCl. The combined organic extracts were dried and the solvent removed *in vacuo*. The crude residue was redissolved in THF (3 mL), cooled to 0 ᵒC, and imidazole (0.18 g, 2.65 mmol) was added followed by water (1 mL). The solution was then stirred at r.t. for 1 h, then partitioned between EtOAc and water. The solution was dried and evaporated and column chromatography with hexanes:EtOAc (1:2) gave 1-(2,6-dimethoxypyridin-4-yl)-3-(1H-imidazol-1-yl)propan-1-one (**VI**) as an oil (0.045 g, 39%). ^1^H NMR (CDCl_3_) δ 7.54 (s, 1H), 7.04 (s, 1H), 6.95 (s, 1H), 6.68 (s, 2H), 4.41 (t, J = 6.4 Hz, 2H), 3.95 (s, 6H), 3.35 (t, J = 6.4 Hz, 2H). Found: [M+H]= 262.6

**1-(2,6-Dimethoxypyridin-4-yl)-3-(1H-1,2,4-triazol-1-yl)propan-1-one (VII)**

Vinylmagnesium bromide (27.9 mL of a 1N solution in THF, 27.9 mmol) was added to a solution of **5** (see above) (3.00 g, 13.3 mmol) in dry THF (30 mL) at 0 °C. The yellow/orange solution was warmed to r.t. for 1.5 h. The solvent was removed *in vacuo* and the residue partitioned between chloroform and water, and the pH adjusted to 1 with 1M HCl. The combined organic extracts were dried and the solvent removed *in vacuo*. The crude residue was redissolved in chloroform (150 mL) and 1*H*-1,2,4-triazole (2.67 g, 39.8 mmol) and the resultant mixture stirred at 60 ᵒC for 3 h. The solution cooled to r.t. and partitioned between chloroform and water. The solution was dried and evaporated and column chromatography with DCM:MeOH (99:1) gave 1-(2,6-dimethoxypyridin-4-yl)-3-(1H-1,2,4-triazol-1-yl)propan-1-one (**VII**) as an oil (1.54 g, 44%). ^1^H NMR (CDCl_3_) δ 8.20 (s, 1H), 7.91 (s, 1H), 6.70 (s, 2H), 4.61 (t, J = 6.2 Hz, 2H), 3.94 (s, 6H), 3.51 (t, J = 6.1 Hz, 2H). Found: [M+H]= 263.6.

**Example synthesis of the compounds of Table 1**

Reagents and conditions: (i) LDA, THF, -75 ^o^C, 1.5 h then the appropriate ketone C/D; (ii) Zn/Zn(CN)_2_, Pd_2_(dba)_3_/P(o-tol)_3_, DMF, 50 ^o^C.

**1-(6-Bromo-2-methoxyquinolin-3-yl)-1-(2,5-dimethoxypyridin-3-yl)-2-(2,6-dimethoxypyridin-4-yl)-4-(dimethylamino)butan-2-ol (18).** n-BuLi (2.92 mL of a 2N solution in cyclohexane, 5.83 mmol) was added at -40 ^o^C under dry nitrogen to a solution of dry diisopropylamine (0.813 mL, 5.83 mmol) in dry THF (6 mL) and the solution was stirred at this temperature for 10 min, then cooled to -78 ^o^C. A solution of 6-bromo-3-((2,5-dimethoxypyridin-3-yl)methyl)-2-methoxyquinoline (**AB-10**) (1.90 g, 4.86 mmol) in dry THF (6 mL) was added dropwise and the mixture was stirred at -78 °C for 90 min, to give a dark, wine-red coloured solution. A solution of 1-(2,6-dimethoxypyridin-4-yl)-3-(dimethylamino)propan-1-one (**IIIa**) (1.15 g, 4.86 mmol) in dry THF (7 mL) was added and the reaction mixture was stirred at this temperature for 4 h. HOAc (0.90 mL) was added and the reaction mixture was warmed to r.t. Water (100 mL) was added and the mixture was extracted with EtOAc (2x). The combined organic extract was washed with sat. aq. NaHCO_3_ solution, and brine, then dried (Na_2_SO_4_) and the solvent removed under reduced pressure. The residue was purified by flash column chromatography. Elution with 0-10% MeOH/DCM afforded isomer A of **18** (1.11 g, 36%) followed by isomer B of **18** (1.03 g, 34%) as white solids.

Isomer A, white solid. ^1^H NMR (CDCl_3_, 400 MHz) δ 8.12 (d, J = 3.3 Hz, 2H), 7.82 (d, J = 2.2 Hz, 1H), 7.68 (d, J = 8.9 Hz, 1H), 7.60 (dd, J = 8.9, 2.2 Hz, 1H), 7.48 (d, J = 3.0 Hz, 1H), 6.56 (br s, 2H), 5.31 (s, 1H), 4.19 (s, 3H), 3.88 (s, 6H), 3.74 (s, 3H), 3.63 (s, 3H), 2.30-2.04 (m, 1H), 2.02-1.96 (m, 1H), 1.97 (s, 6H), 1.83-1.68 (m, 2H). Found: [M+H]= 627.8.

Isomer B, white solid. ^1^H NMR (CDCl_3_, 400 MHz) δ 8.63 (s, 1H), 7.78 (d, J = 1.7 Hz, 1H), 7.68 (d, J = 3.0 Hz, 1H), 7.55-7.47 (m, 3H), 6.56 (br s, 2H), 5.32 (s, 1H), 4.04 (s, 3H), 3.84 (s, 3H), 3.83 (s, 6H), 3.71 (s, 3H), 2.40-2.32 (m, 1H), 2.08 (s, 6H), 2.03-1.98 (m, 1H), 1.87-1.79 (m, 1H), 1.76-1.70 (m, 1H). Found: [M+H]= 627.8.

The mixture was resolved into its four optical isomers using preparative supercritical fluid HPLC at BioDuro LLC (Beijing). The data in Table 1 are for the most active *R*,*S*-diastereomers. The other 6-bromo compounds in Table I were prepared and purified similarly.

**Example of cyanation reaction (ii): 3-(1-(2,5-Dimethoxypyridin-3-yl)-2-(2,6-dimethoxypyridin-4-yl)-4-(dimethylamino)-2-hydroxybutyl)-2-methoxyquinoline-6-carbonitrile (19).**  A solution of compound **18** (Table 1) (0.61 g, 0.969 mmol) in DMF (6 mL, anhydrous) was purged with nitrogen and heated to 55 °C for 10 min Tri(o-tolyl)phosphine (0.044 g, 0.145 mmol), zinc dust (0.006 g, 0.097 mmol) and tris(dibenzylideneacetone)dipalladium(0) (0.067 g, 0.073 mmol) were then added, and the reaction was again purged with nitrogen and heated for another 10 min at 55 °C. Zinc cyanide (0.063 g, 0.533 mmol) was then added and the reaction mixture was heated to 65 °C for 4 hours. The reaction was diluted with water and extracted with EtOAc three times. The organic layer was washed with brine three times, dried and evaporated. Column chromatography with 1:1 hexane/EtOAc followed by 1:3 hexane/EtOAc afforded **19** (0.41 g, 74%) as white solid.

Isomer A, white solid. ^1^H NMR (CDCl_3_, 400 MHz) δ 8.23 (s, 1H), 8.10 (d, J = 3.0 Hz, 1H), 8.05 (d, J = 1.7 Hz, 1H), 7.86 (d, J = 8.7 Hz, 1H), 7.71 (dd, J = 8.6, 1.8 Hz, 1H), 7.49 (d, J = 3.0, 1H), 6.56 (br s, 2H), 5.31 (s, 1H), 4.23 (s, 3H), 3.89 (s, 6H), 3.74 (s, 3H), 3.63 (s, 3H), 2.31-2.24 (m, 1H), 2.02-1.96 (m, 1H), 1.97 (s, 6H), 1.78-1.68 (m, 2H). Found: [M+H]= 574.6.

Isomer B: A solution of compound **18** (Table 1) (0.61 g, 0.969 mmol) in DMF (6 mL, anhydrous) was purged with nitrogen and heated to 55 °C for 10 min Tri(o-tolyl)phosphine (0.044 g, 0.145 mmol), zinc dust (0.006 g, 0.097 mmol) and tris(dibenzylideneacetone)dipalladium(0) (0.067 g, 0.073 mmol) were then added, and the reaction was again purged with nitrogen and heated for another 10 min at 55 °C. Zinc cyanide (0.063 g, 0.533 mmol) was then added and the reaction mixture was heated to 65 °C for 2 hours. The reaction was diluted with water and extracted with EtOAc three times. The organic layer was washed with brine three times, dried and evaporated. Column chromatography with 1:1 hexane/EtOAc followed by 1:3 hexane/EtOAc afforded **19** (0.54 g, 97%) as a foamy solid. Found: [M+H]=573.8

Isomer B, white solid. ^1^H NMR (CDCl_3_, 400 MHz) δ 8.76 (s, 1H), 8.01 (d, J = 1.7 Hz, 1H), 7.70-7.60 (m, 3H), 7.48 (d, J = 3.0 Hz, 1H), 6.55 (br s, 2H), 5.30 (s, 1H), 4.04 (s, 3H), 3.88 (s, 3H), 3.83 (s, 6H), 3.72 (s, 3H), 2.32-2.25 (m, 1H), 2.05 (s, 6H), 2.04-2.00 (m, 1H), 1.88-1.80 (m, 1H), 1.71-1.64 (m, 1H). Found: [M+H]= 574.6.

The mixture was resolved into its four optical isomers using preparative supercritical fluid HPLC at BioDuro LLC (Beijing). The data in Table 1 are for the most active *R*,*S*-diastereomers. The other 6-cyano compounds in Table I were prepared and purified similarly.
